# Dual-seq transcriptomics reveals the battle for iron during *Pseudomonas aeruginosa* acute murine pneumonia

**DOI:** 10.1038/srep39172

**Published:** 2016-12-16

**Authors:** F. Heath Damron, Amanda G. Oglesby-Sherrouse, Angela Wilks, Mariette Barbier

**Affiliations:** 1 Department of Microbiology, Immunology and Cell Biology, West Virginia University School of Medicine, Morgantown WV 26505, USA; 2 Department of Pharmaceutical Sciences, School of Pharmacy, University of Maryland, Baltimore, Maryland, USA; 3 Department of Microbiology and Immunology, School of Medicine, University of Maryland, Baltimore, Maryland, USA

## Abstract

Determining bacterial gene expression during infection is fundamental to understand pathogenesis. In this study, we used dual RNA-seq to simultaneously measure *P. aeruginosa* and the murine host’s gene expression and response to respiratory infection. Bacterial genes encoding products involved in metabolism and virulence were differentially expressed during infection and the type III and VI secretion systems were highly expressed *in vivo*. Strikingly, heme acquisition, ferric-enterobactin transport, and pyoverdine biosynthesis genes were found to be significantly up-regulated during infection. In the mouse, we profiled the acute immune response to *P. aeruginosa* and identified the pro-inflammatory cytokines involved in acute response to the bacterium in the lung. Additionally, we also identified numerous host iron sequestration systems upregulated during infection. Overall, this work sheds light on how *P. aeruginosa* triggers a pro-inflammatory response and competes for iron with the host during infection, as iron is one of the central elements for which both pathogen and host fight during acute pneumonia.

*Pseudomonas aeruginosa* is an opportunistic Gram-negative respiratory pathogen of major clinical significance causing acute and chronic respiratory infections as well as bloodstream, urinary tract, burn wound, and surgical site infections^1^. According to the Center for Disease Control (CDC), 51,000 healthcare-associated *P. aeruginosa* infections occur in the United States each year^2^. This bacterium is highly versatile, able to survive in harsh environments, and forms robust biofilms that are difficult to eradicate on medical devices. *P. aeruginosa* is inherently highly resistant to antibiotics, and effective antimicrobial treatment of infections caused by this bacterium is often challenging. Multidrug-resistant *Pseudomonas* cause 13% of all *P. aeruginosa* infections and were given a threat level of “serious” in the CDC Antibiotic Resistance Threat report in 2013^2^. Overall, *P. aeruginosa* carries one of the highest case fatality rates of all Gram-negative infections^3^. The success of this microorganism is associated with its highly adaptive genome, ability to survive and thrive in diverse environments and the expression of a wide variety of virulence factors^4^,^5^,^6^. It is therefore important to develop new therapeutics for the treatment of *P. aeruginosa* infections.

To accomplish this objective, it is crucial to better understand the simultaneous pathogen and host transcriptional responses during infection. Several studies have attempted to define the interaction dynamics between the pathogen and the host by studying gene and protein expression of either one or the other^7^,^8^,^9^,^10^,^11^. These studies have identified *P. aeruginosa* genes and virulence factors involved in bacterial attachment during colonization, nutrient acquisition, biofilm formation, or resistance to the immune system to facilitate persistence. Numerous studies have also identified how the host responds to infection^12^,^13^,^14^ and shown that pathogen-host interaction drives the selection and co-evolution of the two organisms^15^,^16^,^17^. More recently, Westermann *et al*. performed the first dual-RNA sequencing during tissue culture infection, demonstrating that dual-RNA sequencing is possible and a great tool to study pathogen-host interactions^18^.

However, none of these studies have characterized simultaneously the bacterial and host molecular factors involved in acute lung infection. It has been hypothesized that sequencing the RNA of both the host and the pathogen during *in vivo* infection would be feasible^19^,^20^,^21^ and was recently accomplished during *Toxoplasma gondii* infections^22^. Advances in DNA and protein sequencing during the past decade have now made dual analysis of RNA and protein expression possible, even in complex tissues. To characterize the armamentarium used in the battle between *P. aeruginosa* and the host during acute lung infection, we developed a methodology using dual RNA-seq to identify the transcriptional response of the pathogen and the host during infection.

## Results

### Dual genome-wide gene expression analysis during infection

In this study, we hypothesized that *in vivo* conditions influence gene expression in *P. aeruginosa* during infection and that the bacterium elicits transcriptional changes in the host. To test this hypothesis, we used a murine model of acute pneumonia to characterize the differences in *P. aeruginosa* gene expression during respiratory infection compared to *in vitro* growth. Using dual RNA-seq, we also gained insights into the effects of the bacterium on the host transcriptome. *P. aeruginosa* PAO1 was grown on *Pseudomonas* isolation agar (PIA) at 37 °C and 2 × 10^8^ colony forming units (CFU) were used to infect outbred CD-1 mice by intranasal administration. The *P. aeruginosa* PAO1 grown on PIA was used to purify RNA for *in vitro* growth control. Mice were infected with PAO1, or PBS as control. After 16 h, mice were euthanized, lungs were removed and total RNA was purified. rRNA was depleted, and three libraries were prepared and sequenced using the Illumina MiSeq platform. Read data were mapped to *P. aeruginosa* and *Mus musculus* genomes independently with CLC Genomics.

A total of 87 million 2 × 75 bp reads were obtained. In samples from *in vitro* culture of PAO1 on PIA, 83% of the reads mapped to the pathogen, with a total coverage of 99.1% of the open reading frames of *P. aeruginosa* PAO1. In infected mice, 11% of the reads mapped to *P. aeruginosa*, covering 98.2% of the transcriptome, and 64% of the reads mapped to the mouse transcriptome. A total of 78% of the reads mapped to the non-infected mouse control. These data indicate that the methodology used in this study provides quality RNA in sufficient quantity to study the transcriptomes of both the pathogen and the host in the same sample.

### *P. aeruginosa* gene expression changes during infection

#### Global changes in P. aeruginosa gene expression

EDGE statistical analysis was used to measure differences in gene expression. A total of 1,887 *P. aeruginosa* genes were found to be up-regulated and 1,325 down-regulated *in vivo* compared to *in vitro* growth on PIA with a *p* value < 0.001 (Table S1). To define the systems differentially regulated in each experimental condition, data were analyzed using a wide range of metagenomics analysis tools including GO term analysis of the biological processes, hypergeometric tests on annotations, PseudoCAP, KEGG pathways and STRING analysis^23^,^24^,^25^,^26^.

**Figure 1:**
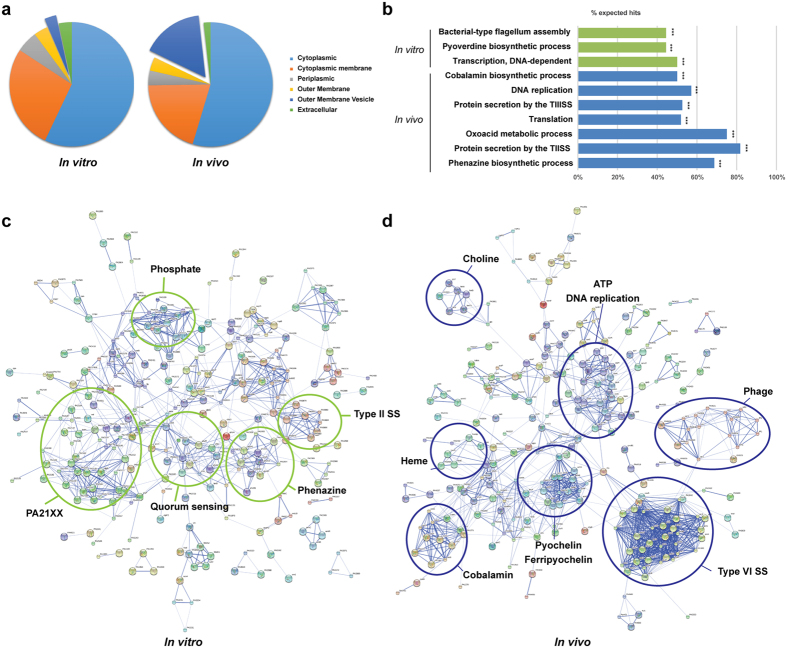
Characterization of *P. aeruginosa* gene expression *in vitro* and *in vivo*. (**a**) Primary cellular localization of the products of the genes of *P. aeruginosa* PAO1 differentially regulated *in vivo* and *in vitro*. The percentage of the total number of genes whose expression was significantly up-regulated *in vivo* compared to *in vitro* are represented on the left; the percentage of genes whose expression was significantly up-regulated *in vitro* compared to *in vivo* are represented on the right. Genes were classified according to their known and predicted cellular localization. (**b**) Hypergeometric test enrichment analysis on annotation of *P. aeruginosa* PAO1 genes differentially regulated *in vitro* and *in vivo*. Data are represented as the percentage of genes (hits) in each class which expression was significantly up-regulated *in vitro* (green) or *in vivo* (blue). The subsets in which the number of hits was significant are denoted with asterisks (***p < 0.001). STRING analysis representing the 300 most highly upregulated *P. aeruginosa* PAO1 genes expressed *in vitro* (**c**) or *in vivo* (**d**). Gene products are represented with circles and known associations between each gene or gene products are represented with a connecting line. Nodes of genes participating in similar functions are circled in green (expressed *in vitro*) or in blue (expressed *in vivo*).

Cellular localization analysis of the gene products expressed by *P. aeruginosa* indicates that the proportion of differentially regulated genes whose products are localized in the cytoplasm, cytoplasmic membrane, periplasm, outer-membrane, and extracellular compartment did not significantly change *in vitro* and during infection (Fig. 1a). However, genes whose products are associated with outer-membrane vesicles (OMV) were significantly up-regulated during infection (29 genes associated with OMV formation or OMV-localized products *in vitro* against 163 genes *in vivo*, Table S2). OMVs are produced under stress conditions^27^ and various authors suggested that OMVs are involved in resistance to the host immune system by absorbing antimicrobial compounds and complement^28^, providing a decoy membrane in the environment^29^ and delivering virulence factors to host cells during infection^30^. Our data seem to indicate that OMV-associated proteins are also important during acute respiratory infection.

#### Changes in gene expression associated with bacterial metabolism

STRING and metabolic analyses indicate that DNA replication, arginine deiminase pathway, gluconeogenesis, cobalamin and choline metabolism, putrescine transport, and polysaccharide biosynthesis processes were significantly up-regulated during infection (Fig. 1b,d). These results suggest, as other have previously hypothesized^11^, that various amino-acids such as arginine, lysine and proline are available *in vivo*, and that *P. aeruginosa* also synthesizes many essential amino-acids during infection. Genes involved in phenylalanine and cellular amide catabolism, as well as cysteine and unsaturated fatty acid biosynthesis were significantly up-regulated *in vitro*. Expression of genes from the region PA21XX^31^, whose products are involved in carbohydrate metabolism, and alginate production, and of other genes involved in stress response such as hypochlorite, oxidative stress, and arsenic-containing substances, were significantly increased *in vitro*, potentially as a result of the stringent growth conditions encountered in PIA (Fig. 1b,c). Pyocyanin expression was also found to be up-regulated *in vitro*, likely as a result of the high magnesium chloride and potassium sulfate concentrations in PIA^32^. Overall these data suggest that *P. aeruginosa* adapts to changes in nutrient availability during infection, primarily as a result of its wide array of metabolic genes.

#### Non-coding RNA expression during infection

*P. aeruginosa* encodes within its genome numerous non-coding RNAs (ncRNA) involved in gene translation such as ribosomal RNAs and transfer RNAs. Other ncRNAs are involved in post-transcriptional regulation such as *crcZ, nalA, phrS, prrF1, prrF2, rsmY*, and *rsmZ*^33^,^34^,^35^. The expression of *crcZ, prrF1, rsmY and rsmZ* was detected both *in vitro* and during infection (RPKM values between 9 and 97,710) (Table S1). The largest number of transcripts detected for a ncRNA in both datasets belonged to *crcZ.* This ncRNA is involved in carbon catabolite repression^33^,^36^ and was significantly down-regulated during infection (15 fold). The expression of the gene encoding for the RNA chaperone Hfq (−1.4 fold) was also found to be significantly down-regulated during infection (Table S1). Altogether, these data suggest that decreased expression of *hfq* and *crcZ* might play a role in carbon utilization changes observed during infection. *rsmZ* was the smallest ncRNA detected (116 bp), suggesting that the method used in this study for RNA extraction and sequencing allows for detection of small ncRNA expression during infection. However, since the RNA extraction method used in this study is optimal for fragments over the size of 200 bp, specialized purification procedures designed for RNA fragments of smaller sizes would likely enhance the characterization of small ncRNAs in this type of samples.

### Expression of virulence factors during infection

**Figure 2:**
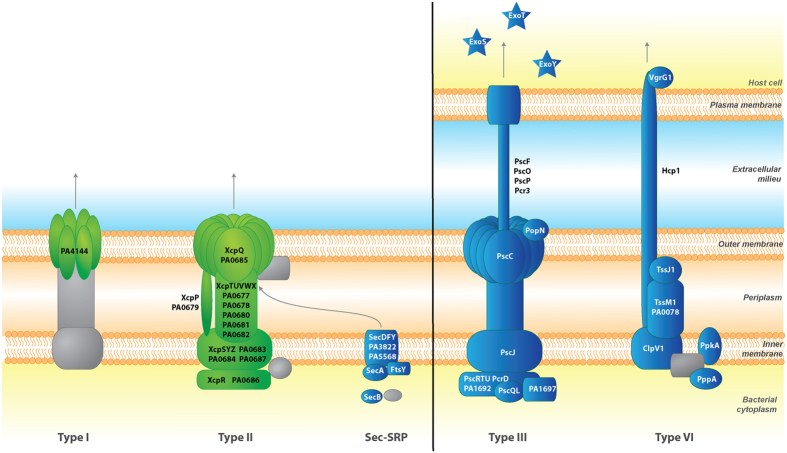
Differential expression of bacterial secretion systems *in vitro* and *in vivo*. *P. aeruginosa* secretion systems expressed *in vitro* and *in vivo* were identified using the Kyoto Encyclopedia of Genes and Genomes (KEGG)^24^. Proteins encoded by genes up-regulated *in vitro* are indicated in green while proteins encoded by genes up-regulated *in vivo* are shown in blue.

*P. aeruginosa* encodes a wide variety of virulence factors for host colonization and to establish infections. As expected, we observed changes in *P. aeruginosa* virulence gene expression during acute murine pneumonia. The expression of genes encoding *P. aeruginosa* type III (T3SS) and type VI (T6SS) secretion systems which translocate effectors across the bacterial cell envelope and the eukaryotic cell membrane was significantly increased *in vivo* (Fig. 2, Table S1). The expression of genes encoding the T3SS secreted effectors ExoS, ExoT, and ExoY was also up-regulated during infection. These two systems are involved in eukaryotic cell targeting and *P. aeruginosa* virulence *in vivo*^37^,^38^. It is important to note that T3SS and T6SS are usually known to be inversely controlled. Genes encoding the type I and type II secretion systems were more predominantly expressed *in vitro* (Fig. 2). Other genes encoding virulence factors such as the potent exotoxin A (*toxA*, 5.3 fold) or flagellar biosynthesis (1.62 to 3.55 fold) were up-regulated during infection. Unlike what was observed in burn wound infections^11^, the expression of the majority of the genes involved in LPS biosynthesis (PA3146-PA3159; 1.55 to 3.51 fold) were also up-regulated during infection. However, multiple genes associated with virulence, which would have been predicted to be expressed during infection, were found to be down-regulated *in vivo*. For instance, the genes encoding the LasR and RhlR systems involved in quorum sensing (QS) and regulation of numerous virulence factors were significantly down-regulated *in vivo* compared to *in vitro* conditions. Not surprisingly, 82 QS-regulated genes identified by Wagner *et al*.^39^ were also down-regulated, representing 46% of the QS regulon. This set includes genes involved in the biosynthesis of virulence factors such as alkaline protease (−1.67 to −4.70 fold), elastase (*lasB*, −154. fold) and protease IV (*piv*, −2.04 fold) (Table S1). This result might be due to differences in cell density between *in vitro* plate and *in vivo* growth conditions. Other non-QS regulated virulence factors were also down-regulated such as phospholipase C (*plcB*, −14.98 fold), alginate biosynthesis enzymes (−2.47 to −7.8 fold), and phenazines (−7.81 to −107.97 fold) (Table S1). These genes have been shown to be important for *P. aeruginosa* virulence in various models including keratitis^40^, burn wound infections^41^, or chronic respiratory infections^42^,^43^. Decreased expression of these virulence factors, together with the changes in T3SS and T6SS expression, could be associated with the down regulation during infection of the two-component system GacA/GacS (−2.0 and −1.5 fold respectively) which controls both acute and chronic virulence determinants such as biofilm formation, T6SS, or exopolysaccharide production^44^,^45^,^46^. The gene encoding RetS, which controls the planktonic lifestyle and coordinates the production of toxins and the T3SS through the small RNAs *rsmZ* and *rsmY*^47^, was significantly upregulated *in vivo.* Interestingly, the expression of the gene encoding for the lost adherence sensor LadS was not significantly changed during infection. Additionally, increases in expression of both T3SS and T6SS during infection could also be associated with population heterogeneity and changes in expression of other regulatory element, such as the up-regulation of the contact-dependent PpkA (3.13 fold) regulation of T6SS^44^. The results obtained here suggest that some virulence factors might not be required for acute pulmonary infections, but might be expressed in later stages, for instance, in association with biofilms in chronic lung infections^44^,^48^.

### *M. musculus* host response during infection

**Figure 3:**
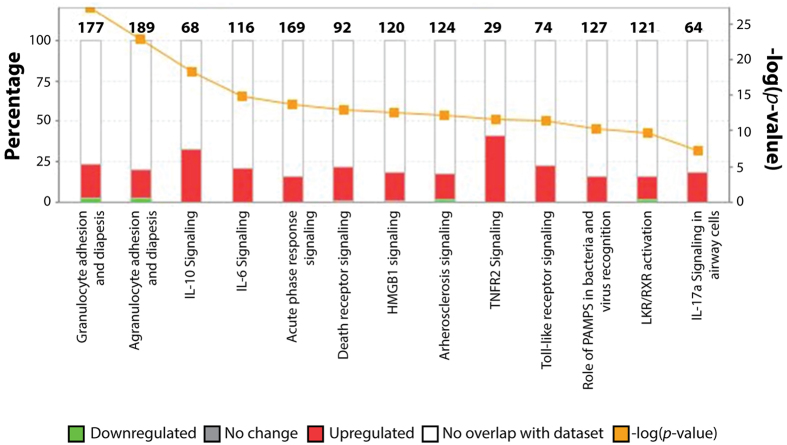
Comparison between host proteomes and transcriptomes in response to *P. aeruginosa* infection. Ingenuity Pathway Analysis (IPA) of canonical pathways significantly up-regulated during *P. aeruginosa* infection detected by transcriptome analysis. Murine genes significantly up- or down-regulated during infection were analyzed using IPA and classified according to their known and predicted function.

During infection, various host factors come into play to control and eliminate bacterial pathogens. To better understand the expression of these factors, EDGE statistical analysis was used to measure differences in gene expression in the lung of non-infected mice and mice infected with *P. aeruginosa*. The expression of 551 *M. musculus* genes increased and the expression of 151 decreased during infection compared to non-infected animals with a *p* value < 0.001. Meta-analysis of the data using hyper-geometric tests indicated that 80% of the genes with increased expression during infection were associated with pro-inflammatory response, chemotaxis, response to antigens, stress response, and regulation of cell proliferation. Granulocyte colony-stimulating factor (G-CSF or CSF3) was the most up-regulated eukaryotic gene during infection with a fold change of 706.23 (*p* = 8.93 × 10^−104^). The expression of genes encoding other cytokines were also significantly increased during infection, including the pro-inflammatory cytokines IL1, IL8, tumor necrosis factor alfa (TNF-α), interferon gamma (IFN-γ), and CSF2. CXC-motif chemokines (CXCL1, 2, 5, 9, 10 and 16), CC-motif chemokines (CCL2, 3, 4, 5, 7, 9, 11, 17 and 20), and the chemokine receptors IL1R2, IL4R, IL17R, and IL18RAP. The genes encoding FAS and TNFR2 were also highly expressed during infection, together with genes whose products are involved in TNF and NFκB signaling (Fig. 3, Table S3). These changes in chemokine expression were likely due to LPS and flagellin recognition by the Toll-like receptors (TLR) 2 and 4^49^,^50^,^51^,^52^,^53^. The genes encoding CD14 (42.55 fold) and MyD88 (5.54 fold), two proteins involved in signaling trough TLR4, had increased expression levels during infection (Fig. 3, Table S3). The expression of the gene encoding TLR2, associated with susceptibility to *P. aeruginosa* infections^54^, was also found to be up-regulated during infection (10.85 fold). These data are in agreement with findings from numerous studies highlighting the importance of Toll-like receptors (TLR) in the recognition of *P. aeruginosa* during infection^55^,^56^,^57^,^58^,^59^. Interestingly, while T3SS effectors, such as ExoS have been shown to inhibit the expression of IL-1β^60^, the expression of this chemokine was up-regulated *in vivo* (13.69 fold). Altogether, chemokine expression is responsible for leukocyte differentiation, activation and recruitment, the production and release of pro-inflammatory cytokines, and play a major role in host defense against *P. aeruginosa*^9^. These chemokines can also be sensed by *P. aeruginosa* proteins such as OprF and affect gene expression in response to proinflammatory signals^61^.

### Battle for iron during infection

#### P. aeruginosa iron acquisition during infection

Iron is a vital nutrient involved in a wide range of biological processes and enzymatic functions. In low iron conditions, *P. aeruginosa* uses siderophores such as pyoverdine and pyochelin to chelate free extracellular Fe^3+^ 62,63,64. Pyoverdine binds Fe^3+^ to form ferripyoverdine. The *fpvA* gene encodes the ferripyoverdine TonB-dependent receptor (TBDR) FpvA. This receptor is bound to the anti-σ factor FpvR that sequesters the σ factors PvdS and FpvI to the bacterial membrane. Binding of ferripyoverdine to FpvA triggers the proteolysis of FpvR, releasing PvdS and FpvI. These σ factors then associate with the RNA polymerase and drive the expression of *fpvA* and the genes responsible for pyoverdine biosynthesis^65^,^66^. In our RNA-seq analysis, the expression of *fpvA* was increased *in vivo* (7.41 fold), while the expression levels of *fpvI, fpvR* and *pvdS* were unchanged (Table S1). This result suggests that *in vivo*, ferripyoverdine binding to FpvA triggers the release of PvdS and FpvI. As a result, pyoverdine expression levels are significantly up-regulated during infection (*pvdADEFGHJLNOPQRT*), supporting established *in vitro* models^65^,^66^. The gene expression of the alternative ferripyoverdine receptor, encoding FpvB, was also up-regulated *in vivo* (4.86 fold) (Table S1). Previous studies have shown the importance of the expression of this system *in vivo*; pyoverdine and FpvA mutants show decreased virulence compared to the parental strain in establishing murine pulmonary infections^67^,^68^. The expression of genes used to produce pyochelin, which has a lower affinity for iron than pyoverdine^63^, were also up-regulated *in vivo*. The *pchABCD* operon was expressed 40 to 50 fold more *in vivo* than *in vitro* and the genes *pchREFG* were expressed over 20 fold more *in vivo. pchA* was found amongst the genes most highly expressed *in vivo* (Table S1).

**Figure 4:**
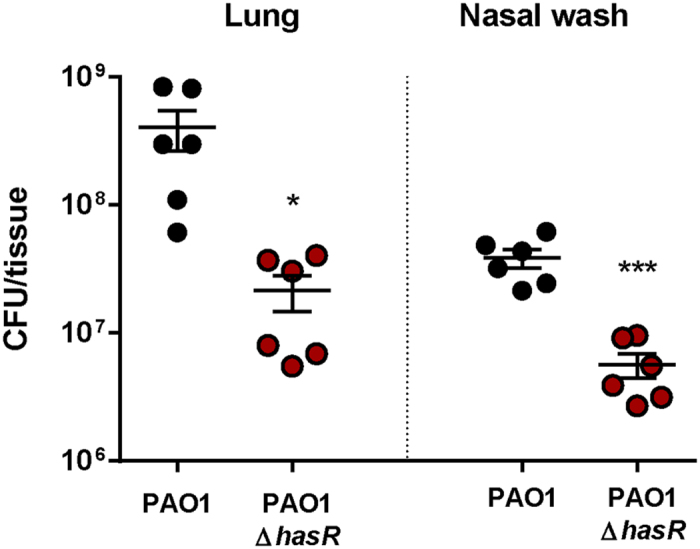
Role of HasR on bacterial burden in mice during acute infection. Mice were infected intranasally with 10^8^ CFU of PAO1Δ*hasR* and the parental PAO1 strain. Bacterial burden were determined in the nares and lung 16 hours post infection. Lung were resuspended in 1 ml PBS and nasal washes were performed with 1 ml of PBS. Data are represented as number of CFU detected in the total volume of sample. Experiments were performed with 6 mice per group and four technical replicates. Data were analyzed using a two-tailed student *t-test* and the software package Graphpad Prism. Statistically significant differences are denoted with asterisks (*p < 0.05; ***p < 0.0001).

**Figure 5:**
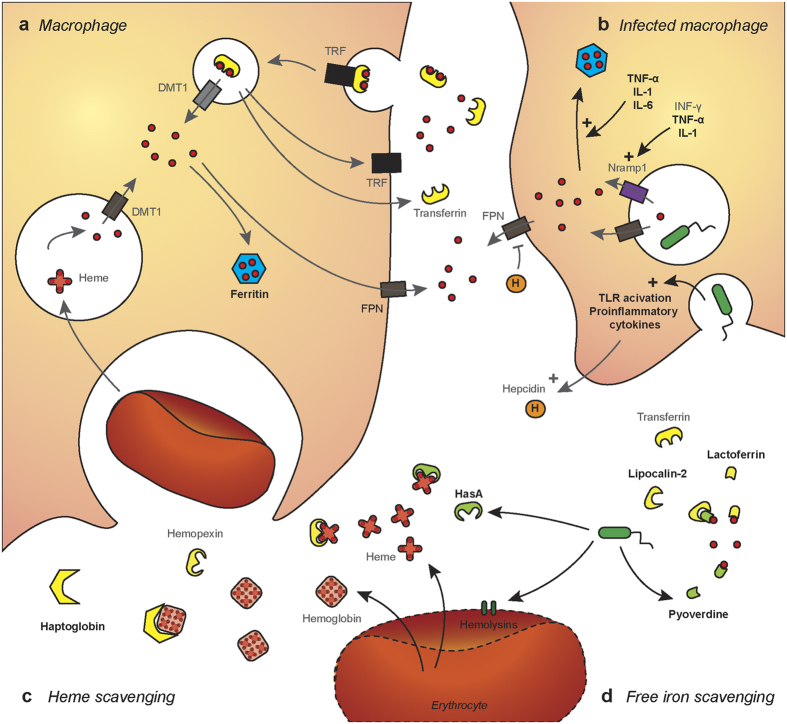
Iron scavenging and regulation *in vivo*. (**a**) Macrophages acquire iron via transferrin receptor (TRF)-mediated endocytosis of transferrin or via recycling of senescent erythrocytes. Macrophages degrade heme using heme oxygenase to generate iron, biliverdin and carbon monoxide. Iron is then transferred to the cytoplasm by DMT1 and either stored using ferritin or released using ferroportin (FPN). (**b**) During infection, bacterial recognition by pattern recognition receptors triggers activation of Toll-like receptors (TLR) and secretion of pro-inflammatory cytokines. As a result, cell release hepcidin to control iron release by FPN. Proinflammatory cyctokines are also involved in the regulation of Nramp1, which together with FPN deprives internalized bacteria of iron. TNF-α, IL-1 and IL-6 also stimulate the storage of iron using ferritin. (**c**) During infection, bacteria can lyse erythrocytes using various hemolysins and toxins to release heme and hemoglobin. The host re-captures these iron-rich proteins using haptoglobin and hemopexin, while *P. aeruginosa* secretes hemophores such as HasA for heme scavenging. (**d**) The host uses lactoferrin and transferrin to limit iron availability during infection. *P. aeruginosa* secretes siderophores with a high iron affinity such as pyoverdine to circumvent iron sequestration. In response, the host releases siderocalin/lipocalin-2 to neutralize bacterial siderophores. Elements that were found to be up-regulated during *P. aeruginosa* infection in the lung are indicated in bold black letters. Elements that were unchanged are shown in grey.

*P. aeruginosa* is also able to obtain heme from hemoproteins using the Has and Phu sytems^69^,^70^. In the Has system, the heme is extracted by the hemophore HasAp, which delivers the iron through the TBDR HasR. In the Phu system, the TBDR PhuR directly extracts the heme. In both pathways, heme is then transported to the cytoplasm by an ABC transporter, bound to a heme chaperone, PhuS, before being delivered to the heme oxygenase HemO^71^. In this RNA-seq study, *hasAp* was the most highly expressed gene *in vivo*, with differences in expression of 317.43 fold compared to *in vitro* settings. The genes *hasD* (228.00 fold), *hasE* (177.53 fold), *hasR* (72.16 fold), *phuR* (27.42 fold), *phuS* (15.00 fold), and *hemO* (6.39 fold) were also highly expressed *in vivo* (Table S1). HemO expression was shown to be necessary to drive the metabolic flow of heme into the cell, with PhuS participating in heme sensing and acting as a control valve for heme intake and degradation^72^. Overall, the Has heme acquisition system was the most highly expressed set of genes *in vivo*, suggesting the importance of iron acquisition from heme during infection. To validate these findings and determine the contribution of the Has system to *P. aeruginosa* pathogenesis, 6 weeks old female CD1 mice were infected intranasally with 10^8^ CFU of PAO1Δ*hasR* or the parental strain PAO1. Sixteen hours post infection, mice were euthanized by pentobarbital injection and the bacterial load in the respiratory tract was determined. In this model of acute respiratory infection, the bacterial load of PAO1Δ*hasR* was 18.89 fold lower in the lung, and 6.8 fold lower in the nasal washes than the parental strain (Fig. 4). These data indicate that a mutant lacking the heme receptor HasR was strongly attenuated during infection. Overall, both RNA-seq and animal infection data suggests, as other have hypothesized in the past^73^, that pyoverdine and the Has/Phu systems are involved in acute respiratory infections and play a major role in *P. aeruginosa* pathogenesis (Fig. 5).

#### Iron sequestration by the host during infection

One of the first lines of defense against bacterial infection is the sequestration of nutrients such as iron to inhibit bacterial growth. During acute infection, the host decreases iron release into the circulation via hepcidin, increases intracellular iron storage using ferritin, decreases extracellular non-heme iron levels via ceruloplasmin and lactoferrin, and decreases extracellular-heme iron levels using hemopexin and haptoglobin. Vertebrates also produce lipocalin-2, or neutrophil gelatinase-associated lipocalin (NGAL), which is secreted by neutrophils in response to infection, and captures bacterial siderophores. In this study, we observed that RNA levels of genes encoding for the expression of ferritin, ceruloplasmin, lactoferrin and haptoglobin are increased 4 to 20 fold during *P. aeruginosa* acute pneumonia (Table 1). However, no changes were observed in ferroportin, hepcidin, hemopexin, transferrin, or transferrin receptor expression. Additionally, the expression of norepinephrine, which increases iron availability, was decreased. These results seem to indicate that during *P. aeruginosa* infection, the host attempts to sequester iron from the pathogen to limit growth and dissemination (Fig. 5).

## Discussion

In this study, we determined the changes in *P. aeruginosa* and host gene expression in the context of an acute murine pneumonia. As expected, we observed that *P. aeruginosa* expressed numerous virulence factors during infection, including T3SS and T6SS. *P. aeruginosa* was found to up-regulate outer membrane vesicle formation and modify its metabolism in response to infection. In the past, we observed that arginine metabolism responded to changes of temperature corresponding to the transition from the environment to the host^7^. In this dataset, we were able to confirm that arginine metabolism gene expression is also increased during infection, suggesting that *P. aeruginosa* adapts its metabolism in response to nutrient availability such as arginine. Overall, there is an important overlap between the data obtained here and gene expression changes observed in acute and chronic burn wounds infections^11^ (T3SS, T6SS, iron acquisition, amino-acid metabolism). This overlap suggests, as others have hypothesized, that while a core set of virulence genes are required to cause infection, *P. aeruginosa* adaptation to the lung requires the coordinated expression of genes involved in both virulence and metabolism^11^,^74^.

Infection with *P. aeruginosa* triggered a strong innate immune response and a cytokine storm mediated by TLR and MyD88 signaling. These data are in agreement with various studies showing that *P. aeruginosa* and outer-membrane vesicles are strong agonists of TLR pathogen-associated molecular pattern receptors^75^,^76^,^77^. Additionally, we observed that the expression of numerous host proteins involved nutritional immunity^78^ were increased during infection. In particular, proteins participating in iron sequestration were up-regulated during infection, including ferritin, ceruloplasmin, lactoferrin, and haptoglobin. Iron is an essential nutrient required by both mammalian hosts and pathogens, and the race for iron is undoubtedly crucial during infection. In the mammalian host, iron is not freely available and is bound to heme molecules found in hemoproteins or chelated by extracellular proteins such as lactoferrin and transferrin^79^. During infection, *P. aeruginosa* sequesters free iron using siderophores and scavenges iron from heme using the Has and Phu heme acquisition systems. One of the most striking observation in this study was the expression levels of the *P. aeruginosa* Has heme acquisition system during infection (over 300 fold up-regulated compared to *in vitro* conditions for *hasAp*). Pyoverdine and pyochelin siderophores, together with the Phu heme acquisition system were also up-regulated, suggesting that bacteria are starved for iron during infection. Up-regulation of both heme and siderophores systems *in vivo* is likely controlled by the master iron regulator Fur, as no significant changes in expression of the ncRNA *prrF1,2* and *prrH* were observed during infection^80^. Additionally, both HasAp and PhuS could also be involved in heme sensing and might be up-regulated during infection in response to the presence of heme in the lung^72^. Numerous studies have demonstrated the importance of siderophores in bacterial virulence and colonization^81^, and authors have shown that pyoverdine production can be detected in the sputum of CF patients^82^. It has also been shown that *P. aeruginosa* interaction with epithelial cells *in vitro* in presence of iron is sufficient to up-regulate pyoverdine acquisition genes^83^. Siderophores have a high affinity for iron, can displace iron from host NGAL and transferrin for iron piracy, and have been shown to also act as toxins^84^. While others have suggested that other iron acquisition systems such as Has and Phu play a role in *P. aeruginosa* pathogenesis^73^, their use as an alternative iron acquisition system during infection has been underappreciated. The data presented here strongly suggests that heme acquisition by the Has/Phu systems play a crucial role for iron acquisition *in vivo*. Others have shown that iron and heme utilization by *P. aeruginosa* depends on disease progression, and suggested that heme acquisition is characteristic of chronic *P. aeruginosa* infections^85^,^86^. Isolates from chronic CF respiratory infections were shown to become more efficient in heme utilization by up-regulating PrrF and HemO expression^86^. In our dataset, heme acquisition also seems to play a major role in acute infection. For this reason, it would be interesting to further define the timing and synergy of heme and iron acquisition systems in *P. aeruginosa* during infection. While siderophores have been used as a therapeutic target^87^,^88^, it is crucial to determine the therapeutic potential of heme acquisition inhibitors in *P. aeruginosa.* Seminal studies have paved the way to understand the role of gene expression in *P. aeruginosa* pathogenesis^11^,^41^,^74^. Current advances in technology now allow us to study bacterial pathogenesis from a more systematic approach^19^. We anticipate that the methodology described in this study will further our understanding of pathogen-host interactions and be applicable in a wide range of systems to facilitate the development of novel therapeutics.

### Experimental procedures

#### Bacterial strains and growth media

*P. aeruginosa* type strain PAO1 was originally obtained from Dr. Michael L. Vasil (University of Colorado). PAO1Δ*hasR* was obtained from Dr. Angela Wilks^70^ (University of Maryland). *P. aeruginosa* was grown in Pseudomonas isolation agar (Becton Dickinson, Franklin Lakes, NJ) for 16h at 37 °C before infection.

#### Infection model

*P. aeruginosa* PAO1 was grown as described above. Bacteria were swabbed off the plate and resuspended in phosphate buffered saline (PBS) at a concentration of 10^10^ colony forming units (CFU) per ml. A fraction of that suspension was centrifuged and resuspended in RNA protect to measure bacterial gene expression *in vitro* before infection. Twenty outbred six weeks old female CD1 mice (Charles River, Frederick, MD) were anesthetized by injection of ketamine and xylazine. Ten microliters of the bacterial suspension were administered to each nostril (10^8^ CFU/mouse). Ten mice were infected with PAO1, and 10 mice were administered PBS vehicle control. Sixteen hours post-infection, mice were euthanized by exposure to carbon dioxide. Lungs were extracted from 4 mice per group and 3 immediately placed in bacterial RNA protect (Qiagen, Germantown, MD) to prevent RNA degradation. The remaining lung was homogenized in PBS and serial dilutions were plated to determine the load in the lung. Sixteen hours post infection, approximately 10^9^ CFU were detected in the lung of each mouse (data not shown). To determine the role of *hasR* on *P. aeruginosa* pathogenesis, mice were infected as described above with 10^8^ CFU of PAO1Δ*hasR* or the parental strain PAO1. After 16 hours of infection, mice were euthanized as described above, and nasal washes and lungs were obtained. Lungs were placed into 1 ml of PBS and homogenized. Nasal washes and lung homogenates were then diluted in PBS, and plated on Lysogeny agar (LA) to determine the number of viable bacteria. Data were analyzed using a two-tailed student *t-test* and the software package Prism 7 (GraphPad, La Jolla, CA). These experiments were performed in accordance with the National Institutes of Health guide for the care and use of laboratory animals. The protocols used were approved by the University of Virginia and West Virginia University Institutional Animal Care and Use Committees.

#### RNA purification and RNA-seq Illumina library preparation and sequencing

RNA was isolated from bacteria grown *in vitro* on PIA plates using RNeasy Mini Kit (Qiagen) as specified by the instructions of the manufacturer. Briefly, cells were pelleted by centrifugation at 8,000 g and RNA protect was pipetted off. Cell were lysed using 200 μl of TE lysozyme for 5 min at room temperature. Cells were then mixed with 700 μl of RLT and 500 μl of 70% ethanol. RNA was then applied to RNeasy purification columns and cleaned with RW1 and RPE buffers. RNA was eluted, treated with RNase-free DNase (Qiagen), and cleaned up on another RNeasy Mini column. RNA was isolated from the lung using the same kit with the following modifications: after removing the RNA protect, each lung was homogenized mechanically for 1 min in 667 μl of TE with 1 mg/ml of lysozyme. A total of 2.33 ml of RLT buffer was then added and tissue was homogenized again for 1 min. Samples were centrifuged at 12,000 g for 10 min and supernatant was transferred to a fresh tube. Five ml of 70% ethanol were added and RNA was purified using 12 RNeasy columns (2 columns per sample). Columns were cleaned with RW1 and RPE buffers, and RNA was eluted. The RNA extracted from the two columns per sample was pooled, treated with RNase-free DNase (Qiagen), and cleaned up using an RNeasy Mini column.

The resulting RNA was quantified on a Nanodrop ND-1000 (Nanodrop, Wilmington, DE). Next, the RNA integrity was assessed using Agilent BioAnalyzer RNA Pico chip (Agilent, Santa Clara, CA). All samples were then submitted to two rounds of eukaryotic and prokaryotic Ribo-zero rRNA depletion (Illumina, San Diego, CA) and reassessed for RNA integrity. rRNA depleted mRNA samples were then fragmented and prepared into libraries using Illumina TruSeq RNA library prep kit v2. Libraries were checked for quality control with KAPA qPCR QC assay (KAPA Biosystems, Wilmington, MA). The libraries were then sequenced on an Illumina MiSeq by the University of Virginia Department of Biology Genomics Core using Illumina MiSeq v3 2 × 75 bp reads. Three MiSeq lanes were used and each lane contained one biological sample of infected *P. aeruginosa* infected lung, one non-infected lung, and one *P. aeruginosa in vitro* sample. A total of three lanes were used to obtain for 81 million 2 × 75 bp reads for the three biological replicates of each group. Sequencing data were deposited to the Sequence Read Archive (SRA) and are available under the reference number SRP090213, BioProject number PRJNA343201.

#### RNA-seq bioinformatics analyses

RNA-seq reads were analyzed using the software CLC Genomics workbench 7.5.1 (Qiagen). The *P. aeruginosa* PAO1 genome and annotations were downloaded from the *Pseudomonas* Genome Database and *Mus musculus* genome was downloaded from NCBI. Reads were mapped against the gene regions of the two genomes using the following settings for mapping: mismatch cost = 2, insertion cost = 3, deletion cost = 3, length fraction = 0.8, similarity fraction = 0.8. RPKM values were generated using default parameters for CLC Genomics. Fold changes in gene expression and statistical analyses were performed using an Extraction of Differential Gene Expression (EDGE) test with the Bonferroni correction^89^.

#### Metabolic and gene set enrichment analyses

Genes with differences in gene expression with a *p* value < 0.001 were mapped on the Kyoto Encyclopedia of Genes and Genomes using KEGG Mapper v.2.5^24^. Gene set enrichment analysis (GSEA) tests were performed using CLC Genomic workbench 7.5.1^23^. Analyses were performed using gene ontology (GO) terms for biological processes^25^, with a total of 10,000 permutations. Primary cellular localization of the gene products was obtained from the *P. aeruginosa* PAO1 genome annotation downloaded from the *Pseudomonas* Genome Database. The 300 genes most highly up and down regulated in the *P. aeruginosa* PAO1 gene expression dataset were analyzed and grouped using the STRING 9.1 database of known and predicted interactions^26^ (string-db.org).

## Additional Information

**How to cite this article**: Damron, F. H. *et al*. Dual-seq transcriptomics reveals the battle for iron during *Pseudomonas aeruginosa* acute murine pneumonia. *Sci. Rep.* **6**, 39172; doi: 10.1038/srep39172 (2016).

**Publisher's note:** Springer Nature remains neutral with regard to jurisdictional claims in published maps and institutional affiliations.

## Supplementary Material

Supplementary Tables

## Figures and Tables

**Table 1:** Host iron-associated genes differentially regulated during *P. aeruginosa* infection.

Gene name	Description	Fold up-regulated during infection	*p-*value
Lcn2	Lipocalin 2	30.85	8.34E-04
Ltf	Lactotransferrin	14.34	2.16E-05
Fth1	Ferritin heavy chain 1	6.27	2.33E-03
Cp	Ceruloplasmin	4.82	2.83E-05
Hba-a1	Hemoglobin alpha, adult chain 1	2.11	3.12E-03
Hba-a2	Hemoglobin alpha, adult chain 2	2.96	3.03E-04
Hbb-bs	Hemoglobin beta adult s chain	2.07	1.98E-03
Hbb-bt	Hemoglobin beta adult t chain	2.54	1.89E-04
Steap4	Metalloreductase able to reduce Fe^3+^ to Fe^2+^ using NAD^+^ as receptor	20.64	4.59E-05
Slc39a14	Soluble metal carrier protein	17.85	2.69E-05
Fbxl5	F-box and leucin-rich repeat protein 5, iron sensor	5.72	1.65E-05
Picalm	Phosphatidylinositol binding clathri assembly protein, transferrin receptor internalization	3.74	3.45E-05
Scd2	Stearoyl-coenzyme A desaturase 2, iron binding and oxydoreductase activity	−1.9	5.54E-05
Mmp8	Metallopeptidase 8	34.28	2.57E-05
Mmp9	Metallopeptidase 9	13.82	2.28E-05

Fold changes were calculated by comparing infected to non-infected mice. Fold changes greater than 1 indicate that the gene was up-regulated during infection compared to *in vitro* growth conditions. Fold changes inferior to −1 indicate that the gene was down-regulated during infection compared to *in vitro* growth conditions.

## References

[b1] Sadikot R. T., Blackwell T. S., Christman J. W. & Prince A. S. Pathogen-host interactions in *Pseudomonas aeruginosa* pneumonia. Am. J. Respir. Crit. Care Med. 171, 1209–23 (2005). 15695491 10.1164/rccm.200408-1044SO PMC2718459

[b2] Centers for Disease Control and Prevention, C. D. C. Antibiotic resistance threats in the United States, 2013. Atlanta CDC (2013).

[b3] Aliaga L., Mediavilla J. D. & Cobo F. A clinical index predicting mortality with *Pseudomonas aeruginosa* bacteraemia. J Med Microbiol 51, 615–619 (2002). 12132781 10.1099/0022-1317-51-7-615

[b4] Behrends V. . Metabolic adaptations of *Pseudomonas aeruginosa* during cystic fibrosis chronic lung infections. Env. Microbiol 15, 398–408 (2013). 22882524 10.1111/j.1462-2920.2012.02840.x

[b5] Mathee K. . Dynamics of *Pseudomonas aeruginosa* genome evolution. Proc. Natl. Acad. Sci. USA 105, 3100–5 (2008). 18287045 10.1073/pnas.0711982105 PMC2268591

[b6] Wolfgang M. C. . Conservation of genome content and virulence determinants among clinical and environmental isolates of *Pseudomonas aeruginosa*. Proc Natl Acad Sci USA 100, 8484–8489 (2003). 12815109 10.1073/pnas.0832438100 PMC166255

[b7] Barbier M. . From the environment to the host: re-wiring of the transcriptome of *Pseudomonas aeruginosa* from 22 degrees C to 37 degrees C. PLoS One 9, e89941 (2014). 24587139 10.1371/journal.pone.0089941 PMC3933690

[b8] Bielecki P. . *In-vivo* expression profiling of *Pseudomonas aeruginosa* infections reveals niche-specific and strain-independent transcriptional programs. PLoS One 6, e24235 (2011). 21931663 10.1371/journal.pone.0024235 PMC3171414

[b9] Lavoie E. G., Wangdi T. & Kazmierczak B. I. Innate immune responses to *Pseudomonas aeruginosa* infection. Microbes Infect 13, 1133–1145 (2011). 21839853 10.1016/j.micinf.2011.07.011 PMC3221798

[b10] Son M. S., Matthews W. J., Kang Y., Nguyen D. T. & Hoang T. T. *In vivo* evidence of *Pseudomonas aeruginosa* nutrient acquisition and pathogenesis in the lungs of cystic fibrosis patients. Infect. Immun. 75, 5313–24 (2007). 17724070 10.1128/IAI.01807-06 PMC2168270

[b11] Turner K. H., Everett J., Trivedi U., Rumbaugh K. P. & Whiteley M. Requirements for *Pseudomonas aeruginosa* acute burn and chronic surgical wound infection. PLoS Genet 10, e1004518 (2014). 25057820 10.1371/journal.pgen.1004518 PMC4109851

[b12] Campodonico V. L., Gadjeva M., Paradis-Bleau C., Uluer A. & Pier G. B. Airway epithelial control of *Pseudomonas aeruginosa* infection in cystic fibrosis. Trends Mol Med 14, 120–133 (2008). 18262467 10.1016/j.molmed.2008.01.002 PMC3697050

[b13] Hartl D. . Innate immunity in cystic fibrosis lung disease. J Cyst Fibros 11, 363–382 (2012). 22917571 10.1016/j.jcf.2012.07.003

[b14] Williams B. J., Dehnbostel J. & Blackwell T. S. *Pseudomonas aeruginosa*: host defence in lung diseases. Respirology 15, 1037–1056 (2010). 20723140 10.1111/j.1440-1843.2010.01819.x

[b15] Barber M. F. & Elde N. C. Nutritional immunity. Escape from bacterial iron piracy through rapid evolution of transferrin. Science. 346, 1362–1366 (2014). 25504720 10.1126/science.1259329 PMC4455941

[b16] Marvig R. L., Sommer L. M., Molin S. & Johansen H. K. Convergent evolution and adaptation of *Pseudomonas aeruginosa* within patients with cystic fibrosis. Nat Genet 47, 57–64 (2015). 25401299 10.1038/ng.3148

[b17] Marvig R. L. . Within-host evolution of *Pseudomonas aeruginosa* reveals adaptation toward iron acquisition from hemoglobin. MBio 5, e00966–14 (2014). 24803516 10.1128/mBio.00966-14 PMC4010824

[b18] Westermann A. J. . Dual RNA-seq unveils noncoding RNA functions in host–pathogen interactions. Nature 529, 496–501 (2016). 26789254 10.1038/nature16547

[b19] Westermann A. J., Gorski S. A. & Vogel J. Dual RNA-seq of pathogen and host. Nat Rev Microbiol 10, 618–630 (2012). 22890146 10.1038/nrmicro2852

[b20] Schulze S., Henkel S. G., Driesch D., Guthke R. & Linde J. Computational prediction of molecular pathogen-host interactions based on dual transcriptome data. Front. Microbiol. 6, 65 (2015). 25705211 10.3389/fmicb.2015.00065 PMC4319478

[b21] Cloney R. Microbial genetics: Dual RNA-seq for host-pathogen transcriptomics. Nat. Rev. Genet. 17, 126–7 (2016). 10.1038/nrg.2016.15 26852807

[b22] Pittman K. J., Aliota M. T. & Knoll L. J. Dual transcriptional profiling of mice and *Toxoplasma gondii* during acute and chronic infection. BMC Genomics 15, 806 (2014). 25240600 10.1186/1471-2164-15-806 PMC4177681

[b23] Tian L. . Discovering statistically significant pathways in expression profiling studies. Proc Natl Acad Sci USA 102, 13544–13549 (2005). 16174746 10.1073/pnas.0506577102 PMC1200092

[b24] Kanehisa M., Sato Y., Kawashima M., Furumichi M. & Tanabe M. KEGG as a reference resource for gene and protein annotation. Nucleic Acids Res. 44, D457–62 (2016). 26476454 10.1093/nar/gkv1070 PMC4702792

[b25] Ashburner M. . Gene ontology: tool for the unification of biology. The Gene Ontology Consortium. Nat Genet 25, 25–29 (2000). 10802651 10.1038/75556 PMC3037419

[b26] Franceschini A. . STRING v9.1: protein-protein interaction networks, with increased coverage and integration. Nucleic Acids Res 41, D808–15 (2013). 23203871 10.1093/nar/gks1094 PMC3531103

[b27] Macdonald I. A. & Kuehn M. J. Stress-induced outer membrane vesicle production by *Pseudomonas aeruginosa*. J Bacteriol 195, 2971–2981 (2013). 23625841 10.1128/JB.02267-12 PMC3697536

[b28] Tan T. T., Morgelin M., Forsgren A. & Riesbeck K. *Haemophilus influenzae* survival during complement-mediated attacks is promoted by *Moraxella catarrhalis* outer membrane vesicles. J Infect Dis 195, 1661–1670 (2007). 17471436 10.1086/517611

[b29] Vidakovics M. L. . B cell activation by outer membrane vesicles-a novel virulence mechanism. PLoS Pathog 6, e1000724 (2010). 20090836 10.1371/journal.ppat.1000724 PMC2799554

[b30] Kulp A. & Kuehn M. J. Biological functions and biogenesis of secreted bacterial outer membrane vesicles. Annu Rev Microbiol 64, 163–184 (2010). 20825345 10.1146/annurev.micro.091208.073413 PMC3525469

[b31] Damron F. H. . Analysis of the *Pseudomonas aeruginosa* regulon controlled by the sensor kinase KinB and sigma factor RpoN. J Bacteriol 194, 1317–1330 (2012). 22210761 10.1128/JB.06105-11 PMC3294845

[b32] KING E. O., WARD M. K. & RANEY D. E. Two simple media for the demonstration of pyocyanin and fluorescin. J. Lab. Clin. Med. 44, 301–7 (1954). 13184240

[b33] Sonnleitner E., Abdou L. & Haas D. Small RNA as global regulator of carbon catabolite repression in *Pseudomonas aeruginosa*. Proc Natl Acad Sci USA 106, 21866–21871 (2009). 20080802 10.1073/pnas.pnas.0910308106 PMC2799872

[b34] Sonnleitner E. & Haas D. Small RNAs as regulators of primary and secondary metabolism in *Pseudomonas* species. Appl. Microbiol. Biotechnol. 91, 63–79 (2011). 21607656 10.1007/s00253-011-3332-1

[b35] Sonnleitner E., Romeo A. & Bläsi U. Small regulatory RNAs in *Pseudomonas aeruginosa*. RNA Biol. 9, 364–71 (2012). 22336763 10.4161/rna.19231

[b36] Sonnleitner E. & Bläsi U. Regulation of Hfq by the RNA CrcZ in *Pseudomonas aeruginosa* carbon catabolite repression. PLoS Genet. 10, e1004440 (2014). 24945892 10.1371/journal.pgen.1004440 PMC4063720

[b37] Shaver C. M. & Hauser A. R. Relative contributions of *Pseudomonas aeruginosa* ExoU, ExoS, and ExoT to virulence in the lung. Infect Immun 72, 6969–6977 (2004). 15557619 10.1128/IAI.72.12.6969-6977.2004 PMC529154

[b38] Mougous J. D. . A virulence locus of *Pseudomonas aeruginosa* encodes a protein secretion apparatus. Science 312, 1526–30 (2006). 16763151 10.1126/science.1128393 PMC2800167

[b39] Wagner V. E., Bushnell D., Passador L., Brooks A. I. & Iglewski B. H. Microarray analysis of *Pseudomonas aeruginosa* quorum-sensing regulons: effects of growth phase and environment. J Bacteriol 185, 2080–2095 (2003). 12644477 10.1128/JB.185.7.2080-2095.2003 PMC151498

[b40] Caballero A., Thibodeaux B., Marquart M., Traidej M. & O’Callaghan R. Pseudomonas keratitis: protease IV gene conservation, distribution, and production relative to virulence and other Pseudomonas proteases. Invest Ophthalmol Vis Sci 45, 522–530 (2004). 14744894 10.1167/iovs.03-1050

[b41] Bielecki P., Glik J., Kawecki M. & Martins dos Santos V. a P. Towards understanding *Pseudomonas aeruginosa* burn wound infections by profiling gene expression. Biotechnol. Lett. 30, 777–90 (2008). 18158583 10.1007/s10529-007-9620-2

[b42] Jaffar-Bandjee M. C. . Production of elastase, exotoxin A, and alkaline protease in sputa during pulmonary exacerbation of cystic fibrosis in patients chronically infected by *Pseudomonas aeruginosa*. J. Clin. Microbiol. 33, 924–9 (1995). 7790462 10.1128/jcm.33.4.924-929.1995 PMC228069

[b43] Hogardt M. & Heesemann J. Microevolution of *Pseudomonas aeruginosa* to a chronic pathogen of the cystic fibrosis lung. Curr. Top. Microbiol. Immunol. 358, 91–118 (2013). 22311171 10.1007/82_2011_199

[b44] Goodman A. L. . A signaling network reciprocally regulates genes associated with acute infection and chronic persistence in *Pseudomonas aeruginosa*. Dev. Cell 7, 745–54 (2004). 15525535 10.1016/j.devcel.2004.08.020

[b45] Lapouge K., Schubert M., Allain F. H.-T. & Haas D. Gac/Rsm signal transduction pathway of gamma-proteobacteria: from RNA recognition to regulation of social behaviour. Mol. Microbiol. 67, 241–53 (2008). 18047567 10.1111/j.1365-2958.2007.06042.x

[b46] Lory S., Merighi M. & Hyodo M. Multiple activities of c-di-GMP in *Pseudomonas aeruginosa*. Nucleic Acids Symp. Ser. (Oxf). 51–2 (2009). 10.1093/nass/nrp026 19749255

[b47] Bordi C. . Regulatory RNAs and the HptB/RetS signalling pathways fine-tune *Pseudomonas aeruginosa* pathogenesis. Mol Microbiol 76, 1427–1443 (2010). 20398205 10.1111/j.1365-2958.2010.07146.x PMC2904497

[b48] Goodman A. L. . Direct interaction between sensor kinase proteins mediates acute and chronic disease phenotypes in a bacterial pathogen. Genes Dev. 23, 249–59 (2009). 19171785 10.1101/gad.1739009 PMC2648536

[b49] Ramphal R. . Control of *Pseudomonas aeruginosa* in the lung requires the recognition of either lipopolysaccharide or flagellin. J Immunol 181, 586–592 (2008). 18566425 10.4049/jimmunol.181.1.586 PMC2504754

[b50] Raoust E. . *Pseudomonas aeruginosa* LPS or flagellin are sufficient to activate TLR-dependent signaling in murine alveolar macrophages and airway epithelial cells. PLoS One 4, e7259 (2009). 19806220 10.1371/journal.pone.0007259 PMC2752798

[b51] Skerrett S. J., Wilson C. B., Liggitt H. D. & Hajjar A. M. Redundant Toll-like receptor signaling in the pulmonary host response to *Pseudomonas aeruginosa*. Am. J. Physiol. Lung Cell. Mol. Physiol. 292, L312–22 (2007). 16936244 10.1152/ajplung.00250.2006

[b52] Ernst R. K. . *Pseudomonas aeruginosa* lipid A diversity and its recognition by Toll-like receptor 4. J. Endotoxin Res. 9, 395–400 (2003). 14733728 10.1179/096805103225002764

[b53] Erridge C., Pridmore A., Eley A., Stewart J. & Poxton I. R. Lipopolysaccharides of *Bacteroides fragilis, Chlamydia trachomatis* and *Pseudomonas aeruginosa* signal via toll-like receptor 2. J. Med. Microbiol. 53, 735–40 (2004). 15272059 10.1099/jmm.0.45598-0

[b54] Pene F. . Toll-like receptor 2 deficiency increases resistance to *Pseudomonas aeruginosa* pneumonia in the setting of sepsis-induced immune dysfunction. J Infect Dis 206, 932–942 (2012). 22782952 10.1093/infdis/jis438

[b55] Faure K. . TLR4 signaling is essential for survival in acute lung injury induced by virulent *Pseudomonas aeruginosa* secreting type III secretory toxins. Respir. Res. 5, 1 (2004). 15040820 10.1186/1465-9921-5-1 PMC389879

[b56] Adamo R., Sokol S., Soong G., Gomez M. I. & Prince A. *Pseudomonas aeruginosa* flagella activate airway epithelial cells through asialoGM1 and toll-like receptor 2 as well as toll-like receptor 5. Am J Respir Cell Mol Biol 30, 627–634 (2004). 14607814 10.1165/rcmb.2003-0260OC

[b57] Soong G., Reddy B., Sokol S., Adamo R. & Prince A. TLR2 is mobilized into an apical lipid raft receptor complex to signal infection in airway epithelial cells. J Clin Invest 113, 1482–1489 (2004). 15146246 10.1172/JCI20773 PMC406530

[b58] Lau G. W. . The *Drosophila melanogaster* toll pathway participates in resistance to infection by the gram-negative human pathogen *Pseudomonas aeruginosa*. Infect. Immun. 71, 4059–66 (2003). 12819096 10.1128/IAI.71.7.4059-4066.2003 PMC162001

[b59] Hajjar A. M., Ernst R. K., Tsai J. H., Wilson C. B. & Miller S. I. Human Toll-like receptor 4 recognizes host-specific LPS modifications. Nat. Immunol. 3, 354–9 (2002). 11912497 10.1038/ni777

[b60] Sutterwala F. S. . Immune recognition of *Pseudomonas aeruginosa* mediated by the IPAF/NLRC4 inflammasome. J Exp Med 204, 3235–3245 (2007). 18070936 10.1084/jem.20071239 PMC2150987

[b61] Wu L. . Recognition of host immune activation by *Pseudomonas aeruginosa*. Science 309, 774–7 (2005). 16051797 10.1126/science.1112422

[b62] Peek M. E., Bhatnagar A., McCarty N. A. & Zughaier S. M. Pyoverdine, the Major Siderophore in *Pseudomonas aeruginosa*, Evades NGAL Recognition. Interdiscip. Perspect. Infect. Dis. 2012, 843509 (2012). 22973307 10.1155/2012/843509 PMC3438788

[b63] Brandel J. . Pyochelin, a siderophore of *Pseudomonas aeruginosa*: physicochemical characterization of the iron(III), copper(II) and zinc(II) complexes. Dalton Trans. 41, 2820–34 (2012). 22261733 10.1039/c1dt11804h

[b64] Cornelis P. Iron uptake and metabolism in pseudomonads. Appl Microbiol Biotechnol 86, 1637–1645 (2010). 20352420 10.1007/s00253-010-2550-2

[b65] Redly G. A. & Poole K. Pyoverdine-mediated regulation of FpvA synthesis in *Pseudomonas aeruginosa*: involvement of a probable extracytoplasmic-function sigma factor, FpvI. J Bacteriol 185, 1261–1265 (2003). 12562796 10.1128/JB.185.4.1261-1265.2003 PMC142879

[b66] Redly G. A. & Poole K. FpvIR control of fpvA ferric pyoverdine receptor gene expression in *Pseudomonas aeruginosa*: demonstration of an interaction between FpvI and FpvR and identification of mutations in each compromising this interaction. J Bacteriol 187, 5648–5657 (2005). 16077110 10.1128/JB.187.16.5648-5657.2005 PMC1196079

[b67] Meyer J. M., Neely A., Stintzi A., Georges C. & Holder I. A. Pyoverdin is essential for virulence of *Pseudomonas aeruginosa*. Infect Immun 64, 518–523 (1996). 8550201 10.1128/iai.64.2.518-523.1996 PMC173795

[b68] Takase H., Nitanai H., Hoshino K. & Otani T. Impact of siderophore production on *Pseudomonas aeruginosa* infections in immunosuppressed mice. Infect Immun 68, 1834–1839 (2000). 10722571 10.1128/iai.68.4.1834-1839.2000 PMC97355

[b69] Ochsner U. A., Johnson Z. & Vasil M. L. Genetics and regulation of two distinct haem-uptake systems, phu and has, In Pseudomonas aeruginosa. Microbiology 146 (Pt 1), 185–198 (2000). 10658665 10.1099/00221287-146-1-185

[b70] Smith A. D. & Wilks A. Differential contributions of the outer membrane receptors PhuR and HasR to heme acquisition in *Pseudomonas aeruginosa*. J Biol Chem 290, 7756–7766 (2015). 25616666 10.1074/jbc.M114.633495 PMC4367277

[b71] Lansky I. B. . The cytoplasmic heme-binding protein (PhuS) from the heme uptake system of *Pseudomonas aeruginosa* is an intracellular heme-trafficking protein to the delta-regioselective heme oxygenase. J Biol Chem 281, 13652–13662 (2006). 16533806 10.1074/jbc.M600824200

[b72] O’Neill M. J. & Wilks A. The *P. aeruginosa* heme binding protein PhuS is a heme oxygenase titratable regulator of heme uptake. ACS Chem. Biol. 8, 1794–802 (2013). 23947366 10.1021/cb400165b PMC3748626

[b73] Cornelis P. & Dingemans J. *Pseudomonas aeruginosa* adapts its iron uptake strategies in function of the type of infections. Front Cell Infect Microbiol 3, 75 (2013). 24294593 10.3389/fcimb.2013.00075 PMC3827675

[b74] Turner K. H., Wessel A. K., Palmer G. C., Murray J. L. & Whiteley M. Essential genome of *Pseudomonas aeruginosa* in cystic fibrosis sputum. Proc. Natl. Acad. Sci. 112, 4110–4115 (2015). 25775563 10.1073/pnas.1419677112 PMC4386324

[b75] Zhao K., Deng X., He C., Yue B. & Wu M. *Pseudomonas aeruginosa* outer membrane vesicles modulate host immune responses by targeting the Toll-like receptor 4 signaling pathway. Infect Immun 81, 4509–4518 (2013). 24082079 10.1128/IAI.01008-13 PMC3837971

[b76] Blohmke C. J. . Innate immunity mediated by TLR5 as a novel antiinflammatory target for cystic fibrosis lung disease. J Immunol 180, 7764–7773 (2008). 18490781 10.4049/jimmunol.180.11.7764

[b77] Schnare M., Rollinghoff M. & Qureshi S. Toll-like receptors: sentinels of host defence against bacterial infection. Int Arch Allergy Immunol 139, 75–85 (2006). 16319494 10.1159/000090001

[b78] Hood M. I. & Skaar E. P. Nutritional immunity: transition metals at the pathogen-host interface. Nat. Rev. Microbiol. 10, 525–37 (2012). 22796883 10.1038/nrmicro2836 PMC3875331

[b79] Cornelissen C. N. & Sparling P. F. Iron piracy: acquisition of transferrin-bound iron by bacterial pathogens. Mol Microbiol 14, 843–850 (1994). 7715446 10.1111/j.1365-2958.1994.tb01320.x

[b80] Oglesby-Sherrouse A. G. & Vasil M. L. Characterization of a Heme-Regulated Non-Coding RNA Encoded by the *prrF* Locus of. PLoS One 5, e9930 (2010). 20386693 10.1371/journal.pone.0009930 PMC2851614

[b81] De Vos D. . Study of pyoverdine type and production by *Pseudomonas aeruginosa* isolated from cystic fibrosis patients: prevalence of type II pyoverdine isolates and accumulation of pyoverdine-negative mutations. Arch Microbiol 175, 384–388 (2001). 11409549 10.1007/s002030100278

[b82] Martin L. W., Reid D. W., Sharples K. J. & Lamont I. L. *Pseudomonas* siderophores in the sputum of patients with cystic fibrosis. Biometals 24, 1059–1067 (2011). 21643731 10.1007/s10534-011-9464-z

[b83] Frisk A. . Transcriptome analysis of *Pseudomonas aeruginosa* after interaction with human airway epithelial cells. Infect. Immun. 72, 5433–8 (2004). 15322041 10.1128/IAI.72.9.5433-5438.2004 PMC517424

[b84] Kirienko N. V., Ausubel F. M. & Ruvkun G. Mitophagy confers resistance to siderophore-mediated killing by *Pseudomonas aeruginosa*. Proc. Natl. Acad. Sci. USA 112, 1821–6 (2015). 25624506 10.1073/pnas.1424954112 PMC4330731

[b85] Konings A. F. . *Pseudomonas aeruginosa* uses multiple pathways to acquire iron during chronic infection in cystic fibrosis lungs. Infect Immun 81, 2697–2704 (2013). 23690396 10.1128/IAI.00418-13 PMC3719594

[b86] Nguyen A. T. . Adaptation of iron homeostasis pathways by a *Pseudomonas aeruginosa* pyoverdine mutant in the cystic fibrosis lung. J Bacteriol 196, 2265–2276 (2014). 24727222 10.1128/JB.01491-14 PMC4054187

[b87] Wurst J. M. . Identification of inhibitors of PvdQ, an enzyme involved in the synthesis of the siderophore pyoverdine. ACS Chem Biol 9, 1536–1544 (2014). 24824984 10.1021/cb5001586 PMC4215858

[b88] Möllmann U., Heinisch L., Bauernfeind A., Köhler T. & Ankel-Fuchs D. Siderophores as drug delivery agents: application of the “Trojan Horse” strategy. Biometals 22, 615–24 (2009). 19214755 10.1007/s10534-009-9219-2

[b89] Robinson M. D. & Smyth G. K. Small-sample estimation of negative binomial dispersion, with applications to SAGE data. Biostatistics 9, 321–32 (2008). 17728317 10.1093/biostatistics/kxm030

