# Supplementary material for: Dual-seq transcriptomics reveals the battle for iron during Pseudomonas aeruginosa acute murine pneumonia
Source: Sci Rep. 2016 Dec 16;6:39172. doi: 10.1038/srep39172 (PMC5159919; doi:10.1038/srep39172)
Supplement: Supplementary Tables [file srep39172-s1.pdf]

SUPPLEMENTARY INFORMATION

Dual-seq transcriptomics reveals the battle for iron during *Pseudomonas aeruginosa* acute murine pneumonia

F. Heath Damron, Amanda G. Oglesby-Sherrouse, Angela Wilks, Mariette Barbier

Table S1: Changes in bacterial gene expression in response to infection measured by RNA-seq. Fold changes greater than 1 indicate that the gene was up-regulated during infection compared to *in vitro* growth conditions. Fold changes inferior to -1 indicate that the gene was down-regulated during infection compared to *in vitro* growth conditions.

Locus	Gene	Fold change	P-value
PA0694	*exbD2*	- inf	3.75E-29
PA0700	*PA0700*	- inf	1.95E-20
PA1355	*PA1355*	- inf	4.89E-21
PA1744	*PA1744*	- inf	4.26E-05
PA2140	*PA2140*	- inf	3.9E-20
PA2141	*PA2141*	- inf	2.45E-21
PA2142a	*PA2142a*	- inf	8.5E-107
PA2149	*PA2149*	- inf	3.57E-50
PA2161	*PA2161*	- inf	9.03E-45
PA2170	*PA2170*	- inf	4.49E-29
PA2183	*PA2183*	- inf	2.85E-46
PA4210	*phzA1*	- inf	3.62E-41
PA3407	*hasAp*	339.4891531	0
PA3406	*hasD*	293.6076547	1.1E-134
PA3405	*hasE*	214.3144174	9.2E-156
PA3408	*hasR*	78.77775938	6E-150
PA3432	*PA3432*	68.45287349	5E-113
PA0046	*PA0046*	66.84638796	2.5E-182
PA0045	*PA0045*	59.74432959	1.1E-118
PA1724	*pscK*	59.64410648	4.73E-64
PA1702	*PA1702*	58.71038873	2.3E-109
PA4219	*PA4219*	58.38546057	1.5E-140
PA1696	*pscO*	58.05630774	7.23E-70
PA4229	*pchC*	50.32981535	2.4E-169
PA4228	*pchD*	49.83010385	4.4E-179
PA4220	*PA4220*	48.71531486	7.5E-127
PA1701	*PA1701*	47.10390838	3.64E-52
PA1700	*PA1700*	45.14675909	6.9E-113
PA4218	*PA4218*	43.72936932	8.9E-167
PA4221	*fptA*	41.90985241	5.8E-204
PA4230	*pchB*	41.25121602	1.5E-229

PA1698	*popN*	40.3665478	1.2E-141
PA4231	*pchA*	40.04091029	7.08E-62
PA3901	*fecA*	40.01679069	4.7E-164
PA1721	*pscH*	38.72981228	2.6E-105
PA1722	*pscI*	37.94947723	8.4E-165
PA1720	*pscG*	34.72730905	4.3E-135
PA0047	*PA0047*	34.17466692	6.1E-134
PA1697	*PA1697*	33.93577148	2.6E-122
PA1717	*pscD*	33.87041724	4E-119
PA0781	*PA0781*	32.46185586	8.68E-81
PA1715	*pscB*	32.21254695	4.36E-88
PA1699	*PA1699*	32.14906382	7.3E-143
PA1723	*pscJ*	31.38739188	9.85E-94
PA4710	*phuR*	29.46457619	1.5E-131
PA1725	*pscL*	29.43609137	4.73E-91
PA0044	*exoT*	28.99746348	4.1E-198
PA3431	*PA3431*	28.18865328	8.33E-68
PA0252	*PA0252*	26.2462289	8.48E-76
PA1692	*PA1692*	25.89662161	3.42E-50
PA4222	*PA4222*	25.72795345	1.5E-125
PA1673	*PA1673*	25.55033365	1.2E-175
PA4223	*PA4223*	25.33171536	1.4E-152
PA1695	*pscP*	24.57378507	6.27E-80
PA1716	*pscC*	24.17878864	1.9E-142
PA4224	*pchG*	24.08912465	3.2E-142
PA4225	*pchF*	23.92644285	1.1E-171
PA1719	*pscF*	23.73656242	2.1E-141
PA3284	*PA3284*	23.72388031	1.4E-223
PA1708	*popB*	23.46316687	2.71E-64
PA1709	*popD*	22.99047857	5.87E-60
PA1556	*ccoO2*	22.05988275	4.4E-206
PA4616	*PA4616*	21.70948942	3.3E-120
PA4226	*pchE*	21.63037759	3.3E-153
PA1321	*cyoE*	21.58802996	2.03E-39
PA1694	*pscQ*	21.35266541	6.4E-68
PA4470	*fumC1*	20.73265425	6.7E-169
PA1913	*PA1913*	20.38996564	3.4E-79
PA1557	*ccoN2*	20.35559448	6.8E-210
PA5374	*betI*	19.96273641	2.1E-158
PA0170	*PA0170*	19.55395549	7.83E-20
PA4227	*pchR*	19.41122071	4.9E-108
PA3877	*narK1*	19.20335595	2.3E-142
PA1718	*pscE*	18.96015184	2.8E-151

PA4836	*PA4836*	18.88411805	9.67E-56
PA1555	*ccoP2*	18.85787323	3.1E-163
PA1691	*pscT*	18.83512065	9.35E-31
PA2203	*PA2203*	18.64286815	7.39E-11
PA3610	*potD*	18.57233132	2.89E-49
PA3283	*PA3283*	18.31026425	1.5E-156
PA1704	*pcrR*	18.16689231	1.95E-44
PA3409	*PA3409*	18.11019455	2.48E-22
PA4895	*PA4895*	17.8386417	1.93E-22
PA5440	*PA5440*	17.72112204	1.4E-136
PA0165	*PA0165*	17.13722144	3.5E-144
PA5373	*betB*	16.78697354	1E-143
PA5372	*betA*	16.54674367	5.9E-137
PA1703	*pcrD*	16.48331315	3.5E-128
PA4709	*PA4709*	16.12258907	3.8E-108
PA1713	*exsA*	15.77754998	1.4E-158
PA4896	*PA4896*	15.57229171	3.89E-25
PA3237	*PA3237*	15.2513981	5.34E-25
PA4599	*mexC*	15.06556594	3.52E-23
PA4837	*PA4837*	15.02333649	6.53E-55
PA1555.1	*ccoQ2*	14.95262457	1.2E-166
PA3841	*exoS*	14.50797821	9.5E-46
PA2463	*PA2463*	14.41908634	8.6E-25
PA3282	*PA3282*	14.38693082	3.56E-62
PA4771	*lldD*	14.30898075	2.31E-91
PA4252	*rplX*	14.10343814	8.31E-65
PA1791	*PA1791*	13.5345806	1.84E-64
PA1196	*PA1196*	13.50402262	7.05E-58
PA2451	*PA2451*	13.1633457	2.3E-115
PA4262	*rplD*	12.85051191	4.73E-76
PA4772	*PA4772*	12.8383315	2.09E-55
PA4386	*groES*	12.82660864	6.3E-175
PA1714	*exsD*	12.62609474	1E-157
PA3404	*PA3404*	12.59653504	7.02E-74
PA4855	*purD*	12.59182528	4.1E-168
PA4932	*rplI*	12.42599807	5.54E-83
PA4933	*PA4933*	12.41559284	1.92E-55
PA1319	*cyoC*	12.41258152	1.13E-40
PA4843	*PA4843*	12.31394549	2.2E-105
PA1228	*PA1228*	12.20183808	3.86E-25
PA1999	*dhcA*	12.19413013	3.7E-155
PA3900	*PA3900*	12.19011776	1.85E-11
PA2453	*PA2453*	11.91279036	1.62E-40

PA3278	*PA3278*	11.88009383	9.43E-64
PA1320	*cyoD*	11.86154614	1.15E-14
PA1277	*cobQ*	11.64908241	1.23E-30
PA4585	*rtcA*	11.61308503	3.58E-27
PA2126	*PA2126*	11.5188207	7.68E-26
PA4256	*rplP*	11.47208015	3.41E-65
PA4690.2	*PA4690.2*	11.40442872	6.57E-16
PA3880	*PA3880*	11.4025707	2.24E-38
PA5369.2	*PA5369.2*	11.2871496	2.4E-17
PA4271	*rplL*	11.19055005	4.18E-69
PA3641	*PA3641*	10.97069899	8.3E-175
PA4257	*rpsC*	10.94734428	1.43E-55
PA4761	*dnaK*	10.94321744	1.76E-47
PA4708	*phuT*	10.93383482	3.8E-103
PA3410	*PA3410*	10.89577291	1.54E-16
PA4613	*katB*	10.80761466	6.63E-82
PA1911	*femR*	10.78522021	1.13E-10
PA4348	*PA4348*	10.75261624	1.4E-176
PA4251	*rplE*	10.61098896	1.18E-74
PA1274	*PA1274*	10.52354887	1.5E-28
PA4280.2	*PA4280.2*	10.51680495	2.81E-29
PA3727	*PA3727*	10.46978605	6.01E-58
PA4254	*rpsQ*	10.42821426	2.54E-57
PA1279	*cobU*	10.38298414	1.26E-27
PA1596	*htpG*	10.3526677	1.9E-169
PA1706	*pcrV*	10.29492997	1.5E-151
PA3608	*potB*	10.26661471	8.78E-22
PA3337	*rfaD*	10.25584227	9.7E-128
PA0545	*PA0545*	10.18691807	1.99E-57
PA4571	*PA4571*	10.15595128	2.8E-131
PA3609	*potC*	10.12217696	5.57E-14
PA5555	*atpG*	10.02693492	8.07E-53
PA5375	*betT1*	9.8931015	3.11E-64
PA1705	*pcrG*	9.84705053	5.47E-95
PA4586	*PA4586*	9.840104501	1.61E-46
PA1276	*cobC*	9.818491108	2.32E-15
PA4155	*PA4155*	9.64812797	4.37E-11
PA4307	*pctC*	9.613404862	8.4E-111
PA4762	*grpE*	9.600370908	6.7E-152
PA4272	*rplJ*	9.514645785	8.32E-60
PA2004	*PA2004*	9.424067734	1.28E-28
PA1270	*PA1270*	9.388715906	2.42E-05
PA4385	*groEL*	9.380673848	9.66E-60

PA1707	*pcrH*	9.336339675	1.8E-152
PA4260	*rplB*	9.295453065	4.28E-61
PA2753	*PA2753*	9.282953395	4.5E-117
PA0171	*PA0171*	9.271731341	3.86E-18
PA4468	*sodM*	9.265359241	2.1E-145
PA4250	*rpsN*	9.206715199	1.2E-210
PA4760	*dnaJ*	9.199396224	7.9E-125
PA2034	*PA2034*	9.16381777	1.45E-55
PA4918	*PA4918*	9.153411051	5.99E-91
PA3742	*rplS*	9.138739083	1.5E-46
PA2000	*dhcB*	9.086790929	9E-141
PA5404	*PA5404*	8.978370376	1.26E-09
PA0277	*PA0277*	8.959335788	1.29E-58
PA4467	*PA4467*	8.829437722	8.5E-135
PA4469	*PA4469*	8.811967203	9.1E-140
PA3600	*PA3600*	8.80061797	1.52E-27
PA2500	*PA2500*	8.652225413	5.19E-30
PA0625	*PA0625*	8.631090875	9.7E-108
PA5139	*PA5139*	8.606228968	2.92E-82
PA2033	*PA2033*	8.579612772	4E-125
PA4261	*rplW*	8.542051598	1.75E-58
PA5553	*atpC*	8.514482906	3.1E-212
PA2459	*PA2459*	8.471031175	2.67E-12
PA4835	*PA4835*	8.377989004	8.89E-22
PA5558	*atpF*	8.342940247	1.07E-42
PA1278	*cobP*	8.330431594	1.57E-21
PA1690	*pscU*	8.187709232	2.29E-16
PA4759	*dapB*	8.12355718	1.2E-134
PA1912	*femI*	8.121715653	9.27E-12
PA4707	*PA4707*	8.112882244	8.04E-55
PA0141	*PA0141*	8.106446738	1.3E-170
PA1922	*PA1922*	8.079171085	0.000452
PA3601	*PA3601*	8.045520837	5.5E-108
PA4583	*PA4583*	8.03552245	2.6E-39
PA0435	*PA0435*	8.028317622	2.37E-14
PA3656	*rpsB*	7.956916088	3.87E-57
PA1317	*cyoA*	7.905414027	1.26E-68
PA1183	*dctA*	7.802709305	8.64E-28
PA2003	*bdhA*	7.771212121	3E-98
PA1546	*hemN*	7.730943922	1.24E-82
PA1275	*cobD*	7.667547436	2.07E-13
PA0639	*PA0639*	7.603263077	3.17E-38
PA3876	*narK2*	7.601207313	3.21E-29

PA5556	*atpA*	7.590522187	1.88E-46
PA4471	*PA4471*	7.549584578	7.2E-101
PA2653	*PA2653*	7.526931847	1.06E-32
PA1281	*cobV*	7.524686959	5.75E-10
PA4854	*purH*	7.521827114	2.3E-149
PA4238	*rpoA*	7.456125941	2.64E-50
PA4894	*PA4894*	7.436465606	0.00042
PA5469	*PA5469*	7.419294505	7.48E-26
PA2398	*fpvA*	7.406655617	4.19E-88
PA2127	*PA2127*	7.297889752	2.34E-77
PA1301	*PA1301*	7.121118861	2.02E-25
PA1280	*PA1280*	7.064889357	1.47E-12
PA3268	*PA3268*	6.999640049	1.26E-20
PA4675	*PA4675*	6.997000637	2.59E-58
PA1273	*cobB*	6.963448783	1.53E-20
PA1771	*estX*	6.947504924	2.11E-17
PA4240	*rpsK*	6.945353013	1.66E-49
PA3899	*PA3899*	6.930931168	3.51E-18
PA3662	*PA3662*	6.88858594	1.3E-22
PA5554	*atpD*	6.841290531	1.7E-44
PA0672	*hemO*	6.812340466	1.8E-136
PA3865a	*PA3865a*	6.811154957	7.06E-26
PA4258	*rplV*	6.800130804	2.48E-67
PA2913	*PA2913*	6.798132889	2.82E-10
PA3839	*PA3839*	6.728557515	5.62E-79
PA0638	*PA0638*	6.728312115	2.37E-31
PA1588	*sucC*	6.69422493	3.53E-49
PA0618	*PA0618*	6.686848486	7.28E-69
PA4577	*PA4577*	6.633283313	1.13E-33
PA1271	*PA1271*	6.613220969	1.84E-23
PA5016	*aceF*	6.602432648	2E-174
PA0465	*creD*	6.593765738	3.62E-16
PA0631	*PA0631*	6.577460027	1.26E-15
PA3840	*PA3840*	6.569000618	1.98E-78
PA4263	*rplC*	6.568728068	5.76E-46
PA3713	*spdH*	6.531298584	1.4E-81
PA4269	*rpoC*	6.462879984	3.01E-67
PA4584	*PA4584*	6.45875024	7.15E-10
PA3913	*PA3913*	6.452195623	6.23E-37
PA0169	*PA0169*	6.430738344	2.94E-14
PA0624	*PA0624*	6.398344181	5.24E-92
PA4239	*rpsD*	6.379406291	5.33E-47
PA5568	*PA5568*	6.374702899	3.5E-195

PA1302	*PA1302*	6.350180816	1.84E-19
PA0636	*PA0636*	6.342126191	4.07E-82
PA4610	*PA4610*	6.340276767	4.25E-48
PA4237	*rplQ*	6.33352945	1.95E-43
PA3728	*PA3728*	6.308840199	3.36E-43
PA3933	*PA3933*	6.269877447	5.64E-50
PA3082	*gbt*	6.246324571	2.11E-58
PA2912	*PA2912*	6.234191234	3.08E-06
PA2204	*PA2204*	6.212100137	7.09E-93
PA3729	*PA3729*	6.204202569	3.9E-124
PA3081	*PA3081*	6.198571782	1.29E-31
PA4273	*rplA*	6.191011462	1.27E-65
PA4744	*infB*	6.170099994	5.22E-34
PA4259	*rpsS*	6.145227289	1.6E-148
PA0637	*PA0637*	6.136651594	3.74E-27
PA4919	*pncB1*	6.105073739	3.5E-115
PA4244	*rplO*	6.094176534	1.07E-42
PA2630	*PA2630*	6.085209284	2.56E-98
PA5557	*atpH*	6.001216563	2.6E-192
PA4935	*rpsF*	5.991251164	2.81E-64
PA0162	*opdC*	5.976975326	1.93E-20
PA2905	*cobH*	5.961671677	1.41E-07
PA0626	*PA0626*	5.959943453	1.03E-33
PA3730	*PA3730*	5.934252513	1.1E-16
PA0628	*PA0628*	5.933946422	2.89E-28
PA3842	*PA3842*	5.907591173	2.1E-134
PA3887	*nhaP*	5.844574649	9.9E-79
PA0782	*putA*	5.83828777	3.1E-116
PA5539	*PA5539*	5.823474593	2.34E-06
PA4266	*fusA1*	5.761006003	8.07E-37
PA4706	*PA4706*	5.760073391	5.74E-24
PA4267	*rpsG*	5.711642152	1.98E-80
PA5535	*PA5535*	5.696920286	3.78E-24
PA0403	*pyrR*	5.677190353	7.57E-24
PA2001	*atoB*	5.658627379	2.9E-142
PA1148	*toxA*	5.6522242	8.9E-119
PA4770	*lldP*	5.650955391	5.08E-30
PA0640	*PA0640*	5.626850441	3.92E-26
PA3925	*PA3925*	5.615818917	2.33E-58
PA0434	*PA0434*	5.614495747	3.21E-13
PA4671	*PA4671*	5.595829497	2.48E-39
PA2739	*pheT*	5.595267953	2.4E-130
PA2202	*PA2202*	5.576010473	1.63E-10

PA0619	*PA0619*	5.551814536	2.8E-84
PA4246	*rpsE*	5.537195396	8.01E-35
PA1318	*cyoB*	5.534483751	4.13E-32
PA0783	*putP*	5.529541236	8.3E-100
PA3820	*secF*	5.512546988	7.3E-171
PA4743	*rbfA*	5.426137286	5.2E-122
PA0755	*opdH*	5.419109352	6.96E-15
PA3607	*potA*	5.396752379	6.58E-13
PA3769	*guaA*	5.381284627	1.9E-172
PA4243	*secY*	5.375350003	2.75E-33
PA1601	*PA1601*	5.368107273	1.18E-17
PA1300	*PA1300*	5.341660241	2.67E-15
PA0641	*PA0641*	5.333825922	1.34E-28
PA5054	*hslU*	5.325013597	1.4E-113
PA2903	*cobJ*	5.316945152	1.09E-09
PA4253	*rplN*	5.30883948	1.86E-64
PA2686	*pfeR*	5.305214443	1.74E-22
PA1693	*pscR*	5.297350302	3.31E-44
PA3281	*PA3281*	5.280275152	1.69E-26
PA5402	*PA5402*	5.276787363	4.37E-15
PA1589	*sucD*	5.273941106	1.24E-62
PA3000	*aroP1*	5.258722011	1.35E-84
PA0024	*hemF*	5.247525844	3.18E-96
PA4688	*hitB*	5.240544163	3.62E-24
PA2402	*PA2402*	5.231436442	4.2E-163
PA0985	*pyoS5*	5.207469632	9.19E-35
PA4168	*fpvB*	5.201317145	1.84E-22
PA5559	*atpE*	5.187200229	3.13E-47
PA5072	*PA5072*	5.179163578	4.01E-36
PA3631	*PA3631*	5.17032439	8.95E-80
PA1805	*ppiD*	5.16072948	3.8E-171
PA2063	*PA2063*	5.148076461	1.19E-11
PA4513	*PA4513*	5.143643813	2.6E-09
PA1115	*PA1115*	5.131756582	2.17E-10
PA0202	*PA0202*	5.127827923	1.82E-08
PA2462	*PA2462*	5.112950122	1.12E-15
PA4241	*rpsM*	5.110951948	2.2E-183
PA4247	*rplR*	5.082182342	4.5E-34
PA0751	*PA0751*	5.051718378	2.16E-08
PA3821	*secD*	5.046724676	1.8E-159
PA0622	*PA0622*	5.018471416	1.4E-115
PA5157	*PA5157*	5.00401407	2.37E-05
PA2850	*ohr*	5.001532846	1.06E-35

PA5235	*glpT*	4.987206322	1.26E-78
PA0634	*PA0634*	4.962193952	5.87E-63
PA4268	*rpsL*	4.960003402	9.82E-71
PA0633	*PA0633*	4.945419753	3.4E-89
PA2976	*rne*	4.945379234	4.4E-90
PA0416	*chpD*	4.925176424	1.38E-13
PA3651	*cdsA*	4.923212201	6.5E-106
PA4270	*rpoB*	4.914644724	1.24E-26
PA3018	*PA3018*	4.91416687	9.17E-31
PA2904	*cobI*	4.901978753	7.01E-24
PA3783	*PA3783*	4.890705837	1.63E-42
PA3912	*PA3912*	4.884626327	1.41E-35
PA4156	*PA4156*	4.884054371	2.21E-07
PA0563	*PA0563*	4.875431177	2.9E-157
PA5504	*PA5504*	4.855614294	9E-79
PA0527	*dnr*	4.854277973	3E-117
PA4673	*PA4673*	4.812600911	9E-130
PA5170	*arcD*	4.791881719	4.7E-50
PA4598	*mexD*	4.783628212	1.58E-06
PA0617	*PA0617*	4.752597893	1.44E-24
PA2392	*pvdP*	4.706795917	6.5E-143
PA4242	*rpmJ*	4.6934931	3.77E-53
PA1602	*PA1602*	4.618575145	4.1E-10
PA4280.5	*PA4280.5*	4.604193966	1.45E-05
PA5207	*PA5207*	4.603845284	1.61E-21
PA1429	*PA1429*	4.589995935	4.28E-30
PA1712	*exsB*	4.583089462	1.04E-74
PA2400	*pvdJ*	4.570848145	3.8E-150
PA4923	*PA4923*	4.55830534	9.63E-34
PA2740	*pheS*	4.54523313	3.5E-125
PA0140	*ahpF*	4.53054852	1.1E-102
PA3866	*PA3866*	4.523005441	2.2E-106
PA2952	*etfB*	4.518458473	1.57E-54
PA3561	*fruK*	4.512081931	2.26E-18
PA0623	*PA0623*	4.48603363	4.95E-30
PA3458	*PA3458*	4.478134158	1.99E-52
PA3562	*fruI*	4.477296252	3.66E-14
PA2399	*pvdD*	4.465985006	4.92E-81
PA3802	*hisS*	4.418822004	1.4E-107
PA0413	*chpA*	4.411901366	9E-103
PA0578	*PA0578*	4.392560763	2.08E-51
PA3643	*lpxB*	4.379639114	5.31E-37
PA0025	*aroE*	4.377252228	1.77E-38

PA4934	*rpsR*	4.360791385	3.42E-84
PA0627	*PA0627*	4.3591729	3.9E-06
PA0126	*PA0126*	4.35873371	8.3E-23
PA0150	*PA0150*	4.357047668	0.000568
PA3124	*PA3124*	4.355577745	4.08E-10
PA1405	*PA1405*	4.353083948	6.7E-06
PA2191	*exoY*	4.352128372	1.03E-39
PA2396	*pvdF*	4.315465833	1.1E-103
PA2389	*pvdR*	4.312155934	3E-102
PA3716	*PA3716*	4.30335417	1.2E-108
PA4920	*nadE*	4.278826891	2.1E-110
PA0048	*PA0048*	4.278674899	3.65E-12
PA4249	*rpsH*	4.258134038	1.33E-83
PA1600	*PA1600*	4.243871794	2.79E-05
PA0073	*PA0073*	4.236758153	1.22E-07
PA2742	*rpmI*	4.23256	2.75E-76
PA0291	*oprE*	4.222107831	2.81E-84
PA3642	*rnhB*	4.218010644	4.01E-33
PA3441	*PA3441*	4.171339499	3.3E-09
PA4621	*PA4621*	4.162397312	7.48E-11
PA1597	*PA1597*	4.152516549	2.01E-42
PA0352	*PA0352*	4.150089939	2.67E-19
PA4248	*rplF*	4.138338677	3.34E-55
PA1161	*rrmA*	4.135961945	9.37E-33
PA3613	*PA3613*	4.124294188	1.6E-104
PA5053	*hslV*	4.106179633	3.23E-44
PA4438	*PA4438*	4.103866674	3.45E-90
PA4283	*recD*	4.082945626	3.08E-17
PA5508	*PA5508*	4.074212226	3.15E-10
PA4004	*PA4004*	4.070321068	2.31E-34
PA0941	*PA0941*	4.067699311	2.62E-08
PA0402	*pyrB*	4.048558077	1.63E-84
PA4720	*trmA*	4.038502571	2.01E-56
PA5002	*PA5002*	4.033440967	9.85E-33
PA0594	*surA*	4.032449658	1.2E-123
PA5208	*PA5208*	4.027257569	2.56E-40
PA4588	*gdhA*	4.024405644	4.27E-52
PA3165	*hisC2*	4.023507498	1E-104
PA4705	*PA4705*	4.005730888	4.63E-17
PA0149	*PA0149*	3.985250786	9.42E-06
PA2951	*etfA*	3.977434884	1.3E-99
PA4724	*PA4724*	3.976139171	1.45E-14
PA0412	*pilK*	3.975940258	4.95E-91

PA0414	*chpB*	3.959333842	2.68E-17
PA2390	*pvdT*	3.956634891	4.08E-92
PA1711	*exsE*	3.944982785	6.7E-39
PA3859	*PA3859*	3.913441439	8.36E-96
PA2687	*pfeS*	3.9084219	1.84E-06
PA1790	*PA1790*	3.905809662	2.07E-28
PA4628	*lysP*	3.895842463	2.76E-25
PA0411	*pilJ*	3.888123352	9.4E-135
PA2996	*nqrD*	3.878589752	8.78E-36
PA3114	*truA*	3.87712045	4E-23
speA_1	*speA_1*	3.85913043	1.01E-78
PA4484	*gatB*	3.848070844	1.1E-109
PA5403	*PA5403*	3.836088823	1.14E-08
PA2385	*pvdQ*	3.831627132	2.62E-94
PA3800	*PA3800*	3.830947231	7.9E-137
PA3879	*narL*	3.830813681	2.82E-32
PA1971	*braZ*	3.827481384	4.53E-06
PA0316	*serA*	3.824695024	4.2E-78
PA4664	*hemK*	3.817800675	1.2E-35
PA3741	*PA3741*	3.817425858	3.83E-17
PA5192	*pckA*	3.817364001	2.53E-93
	lpdG	3.816039031	3.7E-161
PA1959	*bacA*	3.808059922	3.58E-19
PA2840	*PA2840*	3.807325559	8.62E-80
PA2969	*plsX*	3.800113923	8.15E-70
PA3700	*lysS*	3.79071371	1.93E-87
PA5201	*PA5201*	3.789933692	1.31E-73
PA2391	*opmQ*	3.781249692	2.61E-57
PA4665	*prfA*	3.780936187	2.15E-40
PA2119	*PA2119*	3.780684828	1.73E-55
PA3768	*PA3768*	3.76507769	1.83E-56
PA4587	*ccpR*	3.757351522	3.52E-75
PA2413	*pvdH*	3.751503157	2.57E-74
PA3743	*trmD*	3.736041074	3.91E-70
PA0752	*PA0752*	3.734706551	1.88E-05
PA3823	*tgt*	3.726163804	4.69E-31
PA0401	*PA0401*	3.706806582	5.2E-76
PA5536	*PA5536*	3.701958286	1.54E-05
PA3655	*tsf*	3.700666481	2.22E-71
PA3810	*hscA*	3.690214897	3.04E-75
PA5202	*PA5202*	3.686773348	2.8E-11
PA2911	*PA2911*	3.67949699	0.000546
PA2468	*foxI*	3.676975843	2.73E-07

PA0914	*PA0914*	3.67465247	6.5E-06
PA2395	*pvdO*	3.671833705	1.92E-82
PA4428	*sspA*	3.665404471	3.96E-31
PA4432	*rpsI*	3.660972149	2.49E-72
PA0419	*PA0419*	3.652186415	6.24E-19
PA3722	*PA3722*	3.651717406	9.22E-20
PA0428	*PA0428*	3.634587936	1.9E-115
PA3801	*PA3801*	3.615627632	3E-124
PA0973	*oprL*	3.610150914	1.08E-51
PA2995	*nqrE*	3.60590445	9.19E-42
PA2386	*pvdA*	3.603860578	7E-107
PA4479	*mreD*	3.603630365	8.25E-14
PA0620	*PA0620*	3.593691546	1.7E-36
PA3809	*fdx2*	3.57947539	2.41E-36
PA4740	*pnp*	3.578544726	7.43E-90
PA2450	*PA2450*	3.577527801	4.49E-30
PA2229	*PA2229*	3.575610782	5.74E-09
PA4196	*bfiR*	3.574847811	0.000386
PA2446	*gcvH2*	3.572178391	1.1E-47
PA4480	*mreC*	3.571056407	3.37E-23
PA5011	*waaC*	3.565483666	7.2E-33
PA5113	*PA5113*	3.562887249	1.47E-16
PA3336	*PA3336*	3.551231777	3.5E-14
PA1084	*flgI*	3.550087513	1.75E-26
PA2741	*rplT*	3.531158195	1.01E-78
PA5015	*aceE*	3.530325165	7.83E-95
PA1654	*PA1654*	3.527452759	9.24E-16
PA3149	*wbpH*	3.525775741	3.83E-60
PA3147	*wbpJ*	3.518172192	6.69E-43
PA4277	*tufB*	3.517778193	2.01E-97
PA0295	*PA0295*	3.515853067	4.6E-25
PA5564	*gidB*	3.514716992	6.3E-52
PA4568	*rplU*	3.513988314	1.78E-87
PA0018	*fmt*	3.5086088	4.03E-24
PA2929	*PA2929*	3.50714927	4.43E-06
PA4834	*PA4834*	3.505788565	2.6E-06
PA0774	*PA0774*	3.503102569	4.54E-12
PA2973	*PA2973*	3.502192173	5.7E-60
PA4274	*rplK*	3.488836174	6.99E-68
PA0526	*PA0526*	3.481888554	0.000664
PA0201	*PA0201*	3.477382113	1.63E-38
PA4255	*rpmC*	3.474205653	1.34E-66
PA0970	*tolR*	3.470867942	1.44E-39

PA3151	*hisF2*	3.468726257	4.94E-24
PA0008	*glyS*	3.465469188	1.4E-60
PA5003	*PA5003*	3.464240061	2.34E-32
PA4280	*birA*	3.453057007	2.11E-26
PA2321	*PA2321*	3.450440518	5.32E-10
PA4582	*PA4582*	3.444395528	4.33E-16
PA5194	*PA5194*	3.442090961	2.91E-20
PA2002	*PA2002*	3.441781411	8.55E-11
PA5530	*PA5530*	3.434131443	1.17E-58
PA0929	*PA0929*	3.432104113	8.95E-07
PA2481	*PA2481*	3.431579083	1.67E-19
PA5309	*PA5309*	3.428104272	1.34E-10
PA0091	*vgrG1*	3.422872427	1.39E-11
PA1272	*cobO*	3.421674653	8.98E-17
PA5138	*PA5138*	3.420820698	1.91E-25
PA0280	*cysA*	3.418632289	6.97E-12
PA3652	*uppS*	3.414926879	4.76E-79
PA3452	*mqoA*	3.407644435	4.2E-76
PA0768	*lepB*	3.406949809	3.33E-36
PA2393	*PA2393*	3.396074128	1.28E-70
PA3308	*hepA*	3.39371384	2.62E-42
PA5562	*spoOJ*	3.392309444	1.97E-60
PA2611	*cysG*	3.390025405	3.7E-36
PA3148	*wbpI*	3.387961222	5.23E-45
PA2482	*PA2482*	3.385944554	1.3E-10
PA4612	*PA4612*	3.380374666	4.24E-07
PA2624	*idh*	3.379928162	3.71E-65
PA2997	*nqrC*	3.37213968	1.8E-23
PA0075	*pppA*	3.365812545	2.14E-13
PA1586	*sucB*	3.361372674	8.91E-76
PA3287	*PA3287*	3.336033611	2.26E-24
PA0542	*PA0542*	3.334627701	2.93E-65
PA3115	*fimV*	3.334185377	1.38E-90
PA4838	*PA4838*	3.329275436	9.4E-06
PA2567	*PA2567*	3.325616798	5.9E-23
PA4328	*PA4328*	3.31772054	8.37E-30
PA5495	*thrB*	3.317022464	1.19E-44
PA3019	*PA3019*	3.30943652	3.56E-40
PA4003	*pbpA*	3.308326075	1.2E-21
PA3152	*hisH2*	3.30738021	7.96E-13
PA3790	*oprC*	3.307283448	7.21E-45
PA0593	*pdxA*	3.292657694	2.51E-23
PA0836	*ackA*	3.291143474	1.11E-41

PA1240	*PA1240*	3.278402239	0.000731
PA5075	*PA5075*	3.266842122	2.08E-29
PA5560	*atpB*	3.260006141	1.71E-96
PA5049	*rpmE*	3.257712797	1.11E-68
PA4063	*PA4063*	3.250113021	1.62E-18
PA3911	*PA3911*	3.248535969	2.47E-16
PA3731	*PA3731*	3.242354047	1.45E-43
PA5342	*PA5342*	3.241650409	6.86E-10
PA3875	*narG*	3.237069816	1.44E-15
PA0787	*PA0787*	3.224305016	1.77E-06
PA1517	*PA1517*	3.222062855	2.88E-15
PA0086	*PA0086*	3.208344923	2.23E-13
PA3146	*wbpK*	3.198009614	1.96E-24
PA1585	*sucA*	3.195898672	1.11E-78
PA3446	*PA3446*	3.190191691	7.64E-08
PA4065	*PA4065*	3.189926324	3.89E-09
PA4404	*PA4404*	3.188369593	2.55E-16
PA3915	*moaB1*	3.182348218	7.7E-21
PA3824	*queA*	3.176948395	3.34E-22
PA2977	*murB*	3.17489671	3.33E-25
PA4921	*PA4921*	3.171397272	2.8E-10
PA1303	*PA1303*	3.165975131	8.78E-06
PA2994	*nqrF*	3.162531189	1.38E-35
PA5406	*PA5406*	3.159588992	8.42E-26
PA2993	*PA2993*	3.157528633	1.69E-22
PA1361	*PA1361*	3.157380784	2.66E-06
PA5298	*PA5298*	3.150362247	5.74E-37
PA4265	*tufA*	3.148501729	1.89E-88
PA3650	*dxr*	3.147898694	3.55E-39
PA0607	*rpe*	3.141184708	2.15E-27
PA3116	*PA3116*	3.139817799	4.17E-17
PA0931	*pirA*	3.136217545	1.45E-10
PA0310	*PA0310*	3.134746839	3.57E-16
PA0074	*ppkA*	3.12985893	3.25E-10
PA0356	*PA0356*	3.129277545	3.58E-15
PA0433	*PA0433*	3.124079862	2.34E-06
PA0621	*PA0621*	3.123798426	2.58E-14
PA0078	*PA0078*	3.122580037	5.85E-11
PA0615	*PA0615*	3.117283277	9.73E-26
PA0948	*PA0948*	3.109001519	3.6E-12
PA1104	*fliI*	3.099533642	6.08E-19
PA0076	*PA0076*	3.098532884	1.39E-05
PA5425	*purK*	3.098269387	1.8E-36

PA1097	*fleQ*	3.09154578	4.39E-39
PA0616	*PA0616*	3.091391527	6.53E-11
PA4067	*oprG*	3.091349062	5.27E-78
PA3977	*hemL*	3.076696775	1.77E-34
PA1013	*purC*	3.074480806	3.24E-47
PA1746	*PA1746*	3.064949011	1.57E-38
PA5472	*PA5472*	3.060162839	1.47E-16
PA5051	*argS*	3.060086504	1.98E-34
PA4371	*PA4371*	3.049073093	1.14E-10
PA1483	*cycH*	3.047704813	9.44E-24
PA0608	*PA0608*	3.044928421	3.08E-26
PA2759	*PA2759*	3.044540595	2.83E-10
PA5009	*waaP*	3.040383788	3.05E-12
PA4064	*PA4064*	3.036563701	6.47E-06
PA3579	*PA3579*	3.035160621	1.07E-27
PA3129	*PA3129*	3.034069549	5.7E-10
PA1552	*ccoP1*	3.024925215	1.32E-48
PA2978	*ptpA*	3.017021957	2.57E-13
PA2906	*PA2906*	3.008716345	0.000147
PA5343	*PA5343*	2.995014698	5.36E-19
PA5486	*PA5486*	2.993898394	3.41E-10
PA5010	*waaG*	2.987110086	2.53E-28
PA3632	*PA3632*	2.985355504	4.03E-35
PA2735	*PA2735*	2.974409285	2.89E-38
PA1050	*PA1050*	2.973910125	9.76E-20
PA4356	*xenB*	2.971578149	4.21E-13
PA1647	*PA1647*	2.971029796	1.36E-06
PA5012	*waaF*	2.970373061	1.24E-27
PA2480	*PA2480*	2.967398866	8.09E-07
PA4694	*ilvC*	2.962049562	2.51E-51
PA4567	*rpmA*	2.961061578	1.52E-27
PA2394	*pvdN*	2.959307886	1.67E-43
PA5189	*PA5189*	2.958937057	7.54E-06
PA5478	*PA5478*	2.958198835	1.25E-14
PA4768	*smpB*	2.938095304	3.55E-29
PA1140	*PA1140*	2.937299773	1.29E-16
PA5296	*rep*	2.935002736	4.78E-25
PA2667	*PA2667*	2.932911262	7.95E-45
PA4323	*PA4323*	2.928493744	4.53E-12
PA2692	*PA2692*	2.926983225	3.57E-08
PA1056	*PA1056*	2.926767355	3.46E-10
PA0964	*pmpR*	2.924362428	1.37E-47
PA5035	*gltD*	2.917795177	1.71E-24

PA1288	*PA1288*	2.905942227	2.28E-44
PA4051	*thiL*	2.905638526	4.99E-14
PA4162	*PA4162*	2.901108003	0.000401
PA0754	*PA0754*	2.90095411	2.11E-08
PA5004	*PA5004*	2.898423557	1.13E-30
PA3745	*rpsP*	2.887278452	1.08E-27
PA4278	*PA4278*	2.883681905	1.05E-08
PA4322	*PA4322*	2.883003554	4.44E-20
PA0070	*PA0070*	2.880750495	7.84E-39
PA4284	*recB*	2.877023209	6.32E-12
PA4558	*PA4558*	2.875244662	9.06E-36
PA5232	*PA5232*	2.874899658	4.25E-42
PA0387	*PA0387*	2.873205846	1.61E-29
PA3612	*PA3612*	2.869494439	5.32E-10
PA4559	*lspA*	2.86864817	3.14E-29
PA3666	*dapD*	2.868107757	3.33E-35
PA5204	*argA*	2.865386008	2.52E-22
PA1800	*tig*	2.865140556	3.82E-77
PA0390	*metX*	2.85947655	4.57E-27
PA2998	*nqrB*	2.857677958	4.18E-35
PA4988	*waaA*	2.855386056	2.16E-08
PA0944	*purN*	2.843108719	2.74E-33
PA5118	*thiI*	2.843069951	2.45E-30
PA5048	*PA5048*	2.841356871	1.01E-19
PA0969	*tolQ*	2.841323725	8.39E-47
PA2411	*PA2411*	2.83785678	1.19E-50
PA4429	*PA4429*	2.832243654	3.53E-58
PA0410	*pilI*	2.831662156	9.31E-30
PA4754	*PA4754*	2.829509883	3.51E-28
PA3654	*pyrH*	2.827091971	1.4E-31
PA1926	*PA1926*	2.824692972	2.65E-14
PA0409	*pilH*	2.8231592	1.81E-43
PA5315	*rpmG*	2.820337004	6.42E-21
PA4686	*PA4686*	2.814436287	5.78E-25
PA4279	*PA4279*	2.81294475	2.28E-12
PA4358	*PA4358*	2.811884412	3.83E-12
PA4052	*nusB*	2.807365367	1.12E-34
PA5243	*hemB*	2.804768437	7.74E-38
PA5479	*gltP*	2.804376404	1.42E-32
PA3653	*frr*	2.801580308	2.13E-37
PA3604	*erdR*	2.799208827	5.03E-22
PA4272.1	*PA4272.1*	2.797578839	9.11E-35
PA3984	*lnt*	2.794677904	6.99E-14

PA2502	*PA2502*	2.783872998	0.000404
PA2975	*rluC*	2.782537974	3.95E-31
PA5485	*ampDh2*	2.778972313	4.58E-16
PA4292	*PA4292*	2.772183966	7.46E-32
PA0017	*PA0017*	2.771695105	1.33E-17
PA0582	*folB*	2.770283518	4.06E-05
PA4663	*moeB*	2.767053973	1.09E-17
PA4528	*pilD*	2.764945649	1.39E-16
PA5001	*PA5001*	2.758270942	5.42E-14
PA0128	*PA0128*	2.756535672	1.04E-23
PA3732	*PA3732*	2.754608869	4.07E-28
PA3560	*fruA*	2.746273301	0.000164
PA1441	*PA1441*	2.743933769	1.99E-31
PA5336	*gmk*	2.742739611	1.09E-25
PA0382	*micA*	2.740632345	1.84E-15
PA5503	*PA5503*	2.734027541	4.29E-22
PA5109	*PA5109*	2.728534679	1.62E-29
PA0519	*nirS*	2.728143614	4.34E-14
PA2251	*PA2251*	2.726235089	0.000989
PA0892	*aotP*	2.722982271	5.99E-17
PA0079	*PA0079*	2.722271221	2.06E-12
PA4370	*icmP*	2.721544285	4.08E-42
PA4640	*mqoB*	2.72144927	7.21E-26
PA1466	*PA1466*	2.71622711	0.000345
PA3744	*rimM*	2.715937726	7.87E-40
PA3106	*PA3106*	2.712436833	6.69E-27
PA3807	*ndk*	2.712185007	5E-64
PA2734	*PA2734*	2.711920462	9.22E-30
PA5127	*PA5127*	2.711797065	4.14E-17
PA4965	*PA4965*	2.711601031	2.13E-09
PA1132	*PA1132*	2.709811837	3.18E-07
PA0613	*PA0613*	2.709802237	2.43E-07
PA3048	*PA3048*	2.709708765	1.22E-13
PA0771	*era*	2.704712215	5.98E-33
PA5140	*hisF1*	2.70093643	7.76E-24
PA5129	*grx*	2.700527404	2.48E-14
PA0320	*PA0320*	2.697883105	3.05E-13
PA1795	*cysS*	2.697618182	3.23E-34
PA1934	*PA1934*	2.685530156	3.8E-20
PA0090	*clpV1*	2.679725724	6.1E-12
PA4698	*PA4698*	2.674854388	2.91E-08
PA3163	*cmk*	2.670343122	4.07E-32
PA4655	*hemH*	2.669767673	2.65E-20

PA0635	*PA0635*	2.665125848	7.56E-08
PA2964	*pabC*	2.663119082	1.29E-17
PA3228	*PA3228*	2.660797943	5.23E-09
PA2619	*infA*	2.658851523	1.23E-36
PA2885	*atuR*	2.657820172	0.000254
PA3878	*narX*	2.656721339	4.08E-11
PA4373	*PA4373*	2.654540349	5.62E-18
PA1792	*PA1792*	2.65095018	1.17E-15
PA4427	*sspB*	2.650055144	8.07E-25
PA3183	*zwf*	2.650017539	3.21E-32
PA2748	*PA2748*	2.644979451	5.84E-11
PA4569	*ispB*	2.641620485	2.25E-22
PA5236	*PA5236*	2.639404226	7.23E-15
PA3603	*dgkA*	2.635784742	7.01E-06
PA0947	*PA0947*	2.632706157	1.08E-22
PA3028	*moeA2*	2.632221353	4.63E-21
	mdoH	2.630291279	3.57E-31
PA2612	*serS*	2.626275391	4.19E-28
PA4458	*PA4458*	2.625072511	3.5E-28
PA4672	*PA4672*	2.621077372	2.22E-24
PA1549	*PA1549*	2.616406136	9.25E-14
PA1043	*PA1043*	2.605858797	8.84E-07
PA1710	*exsC*	2.605754707	3.55E-25
PA1085	*flgJ*	2.595657266	1.89E-18
PA1012	*PA1012*	2.592415083	1.23E-15
PA0077	*icmF1*	2.589175064	9.73E-09
PA3646	*lpxD*	2.588182039	1.91E-31
PA1460	*motC*	2.581475853	3.09E-17
PA3678	*PA3678*	2.581133389	1.03E-08
PA5052	*PA5052*	2.57515493	9.32E-18
PA0087	*PA0087*	2.574167194	0.000167
PA4372	*PA4372*	2.570590771	1.91E-23
PA4050	*pgpA*	2.569180206	1.36E-05
PA3169	*PA3169*	2.565586624	1.76E-22
PA0895	*aruC*	2.563509842	5.23E-28
PA2974	*PA2974*	2.554641776	2E-14
PA4054	*ribB*	2.55359775	9.36E-25
PA4560	*ileS*	2.552352478	1.44E-30
PA2199	*PA2199*	2.551486451	1.8E-15
PA0834	*PA0834*	2.548672745	1.26E-07
PA3793	*PA3793*	2.547672845	3.54E-09
PA1103	*PA1103*	2.547525937	9.86E-12
PA3951	*PA3951*	2.545216256	0.000394

PA1151	*imm2*	2.544020531	2.59E-16
PA2540	*PA2540*	2.543791929	7.3E-06
PA4745	*nusA*	2.543777033	3.44E-51
PA1687	*speE*	2.543016274	1.93E-14
PA3989	*holA*	2.540088703	4.02E-19
PA0549	*PA0549*	2.539396389	1.63E-18
PA1197	*PA1197*	2.537428857	2.56E-12
PA4390	*PA4390*	2.535051742	6.6E-16
PA3297	*PA3297*	2.534582992	1.39E-09
PA4321	*PA4321*	2.530580258	2.19E-06
PA0369	*PA0369*	2.530369664	4.85E-07
PA5136	*PA5136*	2.530181688	7.8E-07
PA4748	*tpiA*	2.528958493	1.78E-49
PA4996	*rfaE*	2.526803446	2.68E-26
PA5078	*PA5078*	2.526424905	7.35E-36
PA0955	*PA0955*	2.525659184	5.51E-20
PA4595	*PA4595*	2.52243578	1.17E-33
PA3006	*psrA*	2.522056163	5.56E-27
PA3002	*mfd*	2.520688971	5.99E-19
PA1804	*hupB*	2.517493017	2.08E-52
PA1757	*thrH*	2.514262909	3.55E-21
PA0408	*pilG*	2.513660741	1.7E-34
PA5316	*rpmB*	2.50183188	8.68E-28
PA4439	*trpS*	2.495126091	1.65E-24
PA3150	*wbpG*	2.493778252	5.12E-22
PA5335	*PA5335*	2.492535639	1.58E-16
PA5171	*arcA*	2.492376871	7.7E-46
PA4433	*rplM*	2.491883745	1.74E-62
PA4930	*alr*	2.489928663	6.35E-10
PA4730	*panC*	2.488357197	1.23E-20
PA4234	*uvrA*	2.488041107	1.23E-23
PA3861	*rhl*	2.483529145	1.56E-31
PA3735	*thrC*	2.480304561	6.81E-24
PA3162	*rpsA*	2.480218843	2.09E-56
PA1552.1	*ccoQ1*	2.476336414	2.69E-15
PA0085	*hcp1*	2.47387516	2.78E-24
PA0552	*pgk*	2.469921342	2.81E-22
PA3182	*pgl*	2.469912505	2.31E-11
PA4045	*PA4045*	2.468608014	1.25E-08
PA0961	*PA0961*	2.465853058	1.04E-09
PA4411	*murC*	2.465003179	1.74E-24
PA4053	*ribE*	2.464185632	1.32E-39
PA0799	*PA0799*	2.461102805	9.62E-07

PA3634	*PA3634*	2.458509292	7.65E-19
PA0281	*cysW*	2.455458412	0.000291
PA5043	*pilN*	2.454981991	8.22E-28
PA4408	*ftsA*	2.452246673	1.02E-26
PA5074	*PA5074*	2.443836024	9.43E-09
PA5135	*PA5135*	2.441598196	4.35E-06
PA5340	*PA5340*	2.437403712	2.67E-19
PA2454	*PA2454*	2.437234407	2.56E-07
PA1015	*PA1015*	2.436757444	6.65E-08
PA2733	*PA2733*	2.435332151	1.05E-14
PA5044	*pilM*	2.431243158	2.37E-24
PA3134	*gltX*	2.422744693	2.4E-24
PA2648	*nuoM*	2.420438527	8E-21
PA4068	*PA4068*	2.420309054	2.63E-14
PA1452	*flhA*	2.417218033	1.4E-11
PA2044	*PA2044*	2.41674078	5.93E-15
PA2019	*PA2019*	2.416255045	1.96E-05
PA5007	*PA5007*	2.412816186	3E-12
PA3644	*lpxA*	2.40142743	2.1E-28
PA4320	*PA4320*	2.399118486	5.58E-08
PA3822	*PA3822*	2.395456041	2.31E-41
PA5507	*PA5507*	2.394301288	1.58E-06
PA4840	*PA4840*	2.39268337	1.01E-07
PA3659	*PA3659*	2.392380428	1.87E-14
PA1057	*PA1057*	2.391554009	0.000113
PA3633	*ygbP*	2.391192003	3.39E-08
PA0945	*purM*	2.385383606	1.53E-24
PA0020	*PA0020*	2.383794794	3.38E-22
PA5357	*PA5357*	2.382850165	7.97E-09
PA4755	*greA*	2.381040694	3.64E-23
PA1846	*cti*	2.373381353	6.11E-10
PA1011	*PA1011*	2.370161032	2.28E-34
PA4489	*PA4489*	2.368193366	9.97E-10
PA5114	*PA5114*	2.367278652	0.000925
PA4285	*recC*	2.366681142	1.67E-08
PA5304	*dadA*	2.364729565	1.84E-18
PA5141	*hisA*	2.361566836	6.38E-11
PA3931	*PA3931*	2.357924639	4.22E-06
PA4202	*PA4202*	2.355735001	2.88E-07
PA0915	*PA0915*	2.347737165	8.09E-06
PA0400	*PA0400*	2.339742616	1.24E-27
PA4566	*obg*	2.336614717	5.49E-20
PA1105	*fliJ*	2.335752354	0.000161

PA5569	*rnpA*	2.331712638	1.12E-26
PA4264	*rpsJ*	2.32645166	1.72E-22
PA3003	*PA3003*	2.326101725	6.83E-17
PA0775	*PA0775*	2.325791211	4.14E-09
PA4413	*ftsW*	2.325084566	1.75E-14
PA4966	*PA4966*	2.32358502	9.26E-11
PA4415	*mraY*	2.321451381	1.85E-19
PA0767	*lepA*	2.320795858	9.3E-21
PA2770	*PA2770*	2.316600161	3.09E-14
PA0963	*aspS*	2.314346515	5E-27
PA0485	*PA0485*	2.313974353	1.63E-07
PA4565	*proB*	2.313243702	5.79E-21
PA3715	*PA3715*	2.309937334	0.000139
PA0357	*mutY*	2.309084662	2.03E-11
PA4579	*PA4579*	2.30684994	3.72E-07
PA0393	*proC*	2.306665211	1.19E-11
PA0341	*lgt*	2.301886126	1.44E-09
PA4750	*folP*	2.300396601	3.65E-17
PA0399	*PA0399*	2.300193188	2.91E-25
PA0013	*PA0013*	2.298997471	0.000271
PA2970	*rpmF*	2.297285351	4.48E-09
PA0427	*oprM*	2.290386279	4.64E-26
PA5128	*secB*	2.289914346	8.74E-31
PA0437	*codA*	2.283084571	1.5E-08
PA3159	*wbpA*	2.280010286	6.46E-33
PA5117	*typA*	2.272278569	1.49E-34
PA2425	*pvdG*	2.271864014	9.77E-18
PA4431	*PA4431*	2.271248973	6.9E-31
PA3179	*PA3179*	2.267619731	3.59E-19
PA3736	*hom*	2.2672868	6.5E-17
PA4488	*PA4488*	2.267182755	0.000806
PA0835	*pta*	2.263820479	2.41E-19
PA5131	*pgm*	2.253453546	2.48E-21
PA5426	*purE*	2.252308612	6.69E-17
PA3838	*PA3838*	2.252068811	0.000174
PA3014	*faoA*	2.250778795	9.11E-34
PA4066	*PA4066*	2.250292684	6.92E-05
PA1453	*flhF*	2.249812257	5.08E-18
PA2264	*PA2264*	2.248388346	2.06E-10
PA1553	*ccoO1*	2.247054366	1.64E-24
PA2987	*PA2987*	2.244423511	3.63E-15
PA4557	*lytB*	2.243283202	1.89E-17
PA0508	*PA0508*	2.241473663	5.58E-15

PA3046	*PA3046*	2.237332194	4.86E-12
PA3982	*PA3982*	2.236699277	1.12E-15
PA0770	*rnc*	2.236269575	5.23E-19
PA5244	*PA5244*	2.234832241	1.51E-15
PA4662	*murI*	2.233187868	4.4E-07
PA4749	*glmM*	2.232702824	7.87E-19
PA0518	*nirM*	2.230180148	7.09E-05
PA1098	*fleS*	2.226776176	4.96E-13
PA2616	*trxB1*	2.224027578	2.98E-18
PA1044	*PA1044*	2.215448531	9.99E-05
PA4848	*accC*	2.211974826	1.19E-26
PA4853	*fis*	2.211330747	4.18E-10
PA3799	*PA3799*	2.21084302	1.04E-19
PA4719	*PA4719*	2.207371163	8.02E-13
PA3770	*guaB*	2.204953897	6.97E-30
PA0723	*coaB*	2.204621799	2.57E-13
PA0331	*ilvA1*	2.202689245	1.04E-12
PA3172	*PA3172*	2.202508954	4.44E-10
PA4809	*fdhE*	2.202375749	3.5E-05
PA2384	*PA2384*	2.19900674	1.33E-17
PA5549	*glmS*	2.19670632	6.64E-13
PA0373	*ftsY*	2.195064318	3.4E-15
PA2613	*PA2613*	2.192512945	2.37E-11
PA3999	*dacC*	2.1920062	1.23E-22
PA0773	*pdxJ*	2.191131043	8.82E-14
PA3675	*PA3675*	2.180074799	1.07E-12
PA4670	*prs*	2.179971722	1.83E-34
PA3987	*leuS*	2.17731791	5.19E-24
PA4931	*dnaB*	2.172416169	4.25E-17
PA5173	*arcC*	2.170481676	1.87E-27
PA0663	*PA0663*	2.165334577	3.93E-13
PA4728	*folK*	2.16342194	2.26E-08
PA4527	*pilC*	2.163051639	5.01E-17
PA1101	*fliF*	2.159746183	9E-12
PA5130	*PA5130*	2.159674914	3.72E-18
PA4352	*PA4352*	2.158222851	2.38E-17
PA3227	*ppiA*	2.156114577	2.63E-10
PA1563	*PA1563*	2.155524805	5.87E-05
PA1518	*PA1518*	2.154779361	1.84E-09
PA2730	*PA2730*	2.150741958	3.7E-12
PA1099	*fleR*	2.149895094	4.84E-11
PA1584	*sdhB*	2.149445994	2.36E-30
PA0298	*spuB*	2.148538253	5.68E-13

PA3013	*foaB*	2.148084367	4.1E-32
PA1155	*nrdB*	2.145384878	1.95E-24
PA4430	*PA4430*	2.144226821	1.37E-22
PA3811	*hscB*	2.137843878	5.37E-19
PA4410	*ddlB*	2.130193994	2.76E-15
PA1675	*PA1675*	2.128663332	0.00043
PA2843	*PA2843*	2.126331504	7.63E-07
PA4967	*parE*	2.123855535	7.36E-16
PA4494	*roxS*	2.123024999	1.44E-15
PA5005	*PA5005*	2.122846116	1.22E-20
PA4483	*gatA*	2.120127296	4.66E-16
PA5414	*PA5414*	2.118457249	4.08E-10
PA4669	*ipk*	2.117938176	4.09E-15
PA0577	*dnaG*	2.116247716	6.02E-10
PA5146	*PA5146*	2.112366576	2.24E-14
PA2397	*pvdE*	2.10116292	1.98E-14
PA4487	*PA4487*	2.100893482	7.61E-06
PA4572	*fklB*	2.100035251	2.62E-22
PA0579	*rpsU*	2.097627344	1.14E-24
PA1964	*PA1964*	2.094874634	1.9E-15
PA3950	*PA3950*	2.09205362	1.75E-08
PA0789	*PA0789*	2.091435822	0.000487
PA4061	*PA4061*	2.091239955	6.78E-16
PA5025	*metY*	2.085037198	4.11E-08
PA1150	*pys2*	2.084701436	7.12E-11
PA5552	*glmU*	2.082430717	2.03E-16
PA2424	*pvdL*	2.081830637	2.25E-18
PA5231	*PA5231*	2.080759216	1.35E-14
PA1086	*flgK*	2.078793998	1.66E-12
PA4046	*PA4046*	2.075096556	1.58E-09
PA4644	*PA4644*	2.069189403	2.21E-05
PA0080	*PA0080*	2.068376888	0.00022
PA1459	*PA1459*	2.065108686	2.05E-14
PA0004	*gyrB*	2.061199546	1.58E-20
PA4006	*nadD*	2.06116776	1.16E-13
PA4992	*PA4992*	2.060634352	9.22E-06
PA0022	*PA0022*	2.056100757	5.23E-14
PA0943	*PA0943*	2.055714825	1.01E-16
PA1081	*flgF*	2.054826177	5.45E-11
PA0968	*PA0968*	2.053945143	3.85E-10
PA3635	*eno*	2.053810213	3.59E-28
PA2957	*PA2957*	2.053244213	2.29E-12
PA2961	*holB*	2.049273218	3.68E-07

PA3193	*glk*	2.045485049	0.000222
PA0583	*PA0583*	2.045243615	0.000916
PA0389	*PA0389*	2.045128235	1.1E-09
PA5064	*PA5064*	2.045044469	1.31E-07
PA1075	*PA1075*	2.042270579	3.95E-05
PA2754a	*PA2754a*	2.039290438	0.00036
PA5040	*pilQ*	2.038499519	9.32E-23
PA2615	*ftsK*	2.036909473	2.08E-14
PA4548	*PA4548*	2.03589045	9.61E-06
PA1045	*PA1045*	2.033305313	3.8E-06
PA5443	*uvrD*	2.029988558	1.3E-11
PA5428	*PA5428*	2.029911443	1.68E-05
PA3397	*fpr*	2.025198415	4.33E-23
PA0370	*PA0370*	2.023989954	1.06E-08
PA4943	*PA4943*	2.023603647	9.34E-21
PA3167	*serC*	2.021209486	8.96E-18
PA4314	*purU1*	2.02066588	5.11E-13
PA1830	*PA1830*	2.020341717	1.77E-21
PA5046	*PA5046*	2.019957126	5.45E-26
PA4700	*mrcB*	2.016803527	2.44E-07
PA4999	*waaL*	2.015446361	4.1E-06
PA2761	*PA2761*	2.015123467	1.66E-05
PA0415	*chpC*	2.009008339	1.14E-05
PA0971	*tolA*	2.003846179	9.81E-15
PA5223	*ubiH*	2.003700215	3.26E-13
PA3979	*PA3979*	2.003452424	5.29E-06
PA4329	*pykA*	2.002662432	1.51E-16
PA5441	*PA5441*	1.999567722	7.17E-10
PA4417	*murE*	1.995576044	1.92E-13
PA4687	*hitA*	1.992185804	2.76E-13
PA2647	*nuoL*	1.986874085	1.44E-12
PA4449	*hisG*	1.986764122	1.38E-12
PA1415	*PA1415*	1.985912484	4.12E-05
PA4407	*ftsZ*	1.984302213	8.96E-17
PA2642	*nuoG*	1.983673577	1.59E-13
PA0972	*tolB*	1.982623991	1.76E-15
PA4636	*PA4636*	1.981788419	0.000377
PA3763	*purL*	1.98114075	3.63E-14
PA4957	*psd*	1.980747073	4.17E-13
PA4693	*pssA*	1.977915679	1.92E-12
PA3834	*valS*	1.968439029	2.64E-14
PA4811	*fdnH*	1.966859529	1.19E-12
PA4493	*roxR*	1.96673262	2E-16

PA2760	*PA2760*	1.964601398	1.78E-21
PA2795	*PA2795*	1.963794492	0.000865
PA2627	*PA2627*	1.962324349	1.65E-09
PA1614	*gpsA*	1.961593617	3.61E-06
PA3975	*thiD*	1.961260138	6.29E-08
PA0904	*lysC*	1.958219087	2.74E-13
PA0392	*PA0392*	1.954749078	1.25E-06
PA4542	*clpB*	1.953144087	2.62E-13
PA4457	*PA4457*	1.951637022	2.17E-13
PA1534	*recR*	1.949822279	8E-09
PA1766	*PA1766*	1.949718482	1.37E-12
PA1299	*PA1299*	1.943138389	7.48E-05
PA3001	*PA3001*	1.943053687	3.74E-25
PA5034	*hemE*	1.94081099	1.32E-11
PA3435	*PA3435*	1.938597763	2.56E-07
PA4387	*PA4387*	1.938120659	6.82E-08
PA0426	*mexB*	1.93704799	9.76E-13
PA4030	*PA4030*	1.928707686	0.000504
PA3817	*PA3817*	1.928122399	2.29E-05
PA1461	*motD*	1.927559139	1.34E-05
PA3803	*gcpE*	1.925909962	9.76E-12
PA0913	*mgtE*	1.925788732	2.73E-06
PA4852	*PA4852*	1.919928482	1.35E-13
PA4245	*rpmD*	1.913366307	9.16E-21
PA4461	*PA4461*	1.90782083	1.54E-14
PA3164	*PA3164*	1.900137787	1.3E-13
PA4448	*hisD*	1.89984943	6.68E-07
PA2408	*PA2408*	1.895637833	2.25E-07
PA5531	*tonB1*	1.89259157	9.37E-06
PA2629	*purB*	1.892408718	1.43E-12
PA1794	*glnS*	1.886711646	5.87E-12
PA2645	*nuoJ*	1.884406032	9.87E-08
PA4005	*PA4005*	1.880000877	6.22E-10
PA0898	*aruD*	1.879660476	2.46E-06
PA5260	*hemC*	1.873598442	1.93E-10
PA5193	*yrfI*	1.869967604	2.53E-07
PA1193	*PA1193*	1.867397674	0.000128
PA3640	*dnaE*	1.866705148	8.2E-11
PA0580	*gcp*	1.865824178	5.1E-08
PA0779	*PA0779*	1.862809639	3.95E-08
PA0891	*PA0891*	1.862394704	3.94E-05
PA3309	*PA3309*	1.861245494	3.14E-20
PA3194	*edd*	1.860916632	2.58E-09

PA2641	*nuoF*	1.858294567	3.31E-09
PA2377	*PA2377*	1.858228384	0.000118
PA5494	*PA5494*	1.853153817	4.51E-10
PA3647	*PA3647*	1.851658435	2.66E-12
PA1796	*folD*	1.851462214	8.71E-09
PA0374	*ftsE*	1.851233145	8.6E-10
PA3804	*PA3804*	1.848460732	2.4E-08
PA0375	*ftsX*	1.848343758	2.87E-08
PA2436	*PA2436*	1.846424692	0.000323
PA0541	*PA0541*	1.845847905	2.59E-09
PA4695	*ilvH*	1.844692321	1.06E-08
PA4412	*murG*	1.8419205	1.57E-05
PA4275	*nusG*	1.841185233	1.27E-14
PA4960	*PA4960*	1.84070831	2.13E-09
PA4656	*PA4656*	1.839577614	4.84E-05
PA5036	*gltB*	1.835917744	5.98E-09
PA1080	*flgE*	1.833979477	3.06E-09
PA2412	*PA2412*	1.832982138	7.21E-17
PA4481	*mreB*	1.832123082	1.09E-10
PA3563	*fruR*	1.831487645	0.000171
PA0431	*PA0431*	1.829893679	1.01E-09
PA4414	*murD*	1.829460369	1.96E-09
PA3117	*asd*	1.828801895	5.12E-09
PA3453	*PA3453*	1.821149185	5.07E-06
PA4460	*PA4460*	1.819902324	4.33E-11
PA4810	*fdnI*	1.81884039	1.04E-05
PA2290	*gcd*	1.815778434	1.97E-09
PA2323	*PA2323*	1.814058542	6.86E-08
PA1480	*ccmF*	1.814007519	3.95E-07
PA2851	*efp*	1.811969793	4.06E-13
PA0658	*PA0658*	1.810250557	0.000111
PA5042	*pilO*	1.809460754	1.95E-08
PA5274	*rnk*	1.809271797	4.18E-10
PA5065	*ubiB*	1.807995131	0.000143
PA4741	*rpsO*	1.807673784	2.47E-15
PA1116	*PA1116*	1.80695187	3.3E-07
PA2983	*PA2983*	1.801119524	9.08E-08
PA1583	*sdhA*	1.801042035	2.71E-14
PA5334	*rph*	1.800953412	7.77E-09
PA3158	*wbpB*	1.798286513	1.7E-11
PA0896	*aruF*	1.7969827	8.21E-08
PA1767	*PA1767*	1.795911424	4.85E-09
PA4997	*msbA*	1.794668695	2.13E-08

PA3173	*PA3173*	1.793649684	3.47E-07
PA3154	*wzy*	1.793626151	0.000152
PA5041	*pilP*	1.789777958	1.07E-08
PA0705	*migA*	1.784594812	4.43E-08
PA3483	*PA3483*	1.784081126	6.49E-05
PA4729	*panB*	1.78106027	0.000701
PA3413	*PA3413*	1.780068036	0.000555
PA5345	*recG*	1.779966718	0.000977
PA3626	*PA3626*	1.778877421	9.68E-06
PA4647	*uraA*	1.778015658	1.44E-05
PA4491	*PA4491*	1.776717492	0.000278
PA0668	*tyrZ*	1.770816834	1.26E-09
PA0354	*PA0354*	1.764959325	1.54E-07
PA4756	*carB*	1.762353611	1.34E-09
PA3997	*lipB*	1.761510886	6.18E-07
PA1162	*dapE*	1.759635896	8.26E-07
PA4727	*pcnB*	1.758373995	1.61E-08
PA3440a	*PA3440a*	1.754727524	0.000394
PA5221	*PA5221*	1.752703846	0.000374
PA4964	*parC*	1.75249571	1.96E-07
PA0609	*trpE*	1.750763252	6.36E-07
PA3645	*fabZ*	1.749643381	1.12E-08
PA2409	*PA2409*	1.74314233	1.43E-06
PA4031	*ppa*	1.742929212	2.52E-13
PA4459	*PA4459*	1.739907322	7.09E-07
PA2407	*PA2407*	1.739869056	6.22E-07
PA1083	*flgH*	1.734846906	4.48E-05
PA0576	*rpoD*	1.733868972	7.39E-15
PA2979	*kdsB*	1.727602113	0.000454
PA1102	*fliG*	1.725254978	4.72E-07
PA5215	*gcvT1*	1.724452399	5.49E-07
PA4317	*PA4317*	1.723331014	2.28E-06
PA5110	*fbp*	1.722347718	2.16E-10
PA4001	*sltB1*	1.720623884	2.66E-05
PA0872	*phhA*	1.715911591	5.85E-07
PA5019	*PA5019*	1.714016794	2.85E-05
PA4602	*glyA3*	1.707825297	3.79E-10
PA2999	*nqrA*	1.707134182	1.8E-06
PA2732	*PA2732*	1.704701321	0.000128
PA3181	*PA3181*	1.704067118	0.000709
PA0449	*PA0449*	1.703485604	6.94E-07
PA0651	*trpC*	1.703372167	3.6E-06
PA4418	*ftsI*	1.699965409	5.51E-06

PA3827	*PA3827*	1.697800043	1.13E-05
PA5000	*wapR*	1.694200724	2.89E-06
PA3107	*metZ*	1.692270667	5.62E-06
PA3262	*PA3262*	1.690849816	8.25E-12
PA2649	*nuoN*	1.690199968	4.72E-05
PA1457	*cheZ*	1.688452385	2.85E-07
PA2614	*lolA*	1.687182256	7.86E-07
PA5142	*hisH1*	1.684877927	8.38E-06
PA4850	*prmA*	1.683014195	0.000192
PA0337	*ptsP*	1.683009371	1.59E-06
PA5331	*pyrE*	1.679588835	0.000154
PA3263	*PA3263*	1.676248359	1.47E-06
PA4333	*PA4333*	1.667091362	5.17E-07
PA1532	*dnaX*	1.662535755	5.62E-06
PA0432	*sahH*	1.659087295	2.14E-11
PA4952	*PA4952*	1.657179164	6.53E-05
PA1554	*ccoN1*	1.657148942	5.38E-10
PA3988	*PA3988*	1.644133582	1.18E-06
PA0758	*PA0758*	1.641106019	4.07E-05
PA5291	*PA5291*	1.638057634	0.000225
PA4544	*rluD*	1.634666584	0.000188
PA1082	*flgG*	1.626458297	0.000233
PA0778	*icp*	1.624584424	0.000805
PA1049	*pdxH*	1.621433399	6.1E-06
PA5563	*soj*	1.61395533	0.000335
PA3285	*PA3285*	1.604837285	0.000942
PA0660	*PA0660*	1.603839262	5.66E-05
PA5322	*algC*	1.597642438	0.00013
PA5172	*arcB*	1.597022342	3.59E-09
PA4416	*murF*	1.592914012	0.000191
PA1793	*ppiB*	1.588114166	3.49E-06
PA4409	*ftsQ*	1.580421018	0.000705
PA3940	*PA3940*	1.577879288	3.64E-06
PA4450	*murA*	1.573529751	4.16E-07
PA2743	*infC*	1.571783637	2.4E-11
PA0956	*proS*	1.56623625	5.55E-06
PA1580	*gltA*	1.562335281	5.76E-05
PA0903	*alaS*	1.561694568	0.000271
PA1787	*acnB*	1.560091021	3.79E-07
PA0009	*glyQ*	1.559707413	0.000155
PA3155	*wbpE*	1.557322881	0.000392
PA3168	*gyrA*	1.55556815	1.12E-05
PA1551	*PA1551*	1.554695671	0.000422

PA2626	*trmU*	1.541603395	0.000919
PA0425	*mexA*	1.539776548	0.00025
PA4938	*purA*	1.534006469	4.15E-07
PA4406	*lpxC*	1.527892611	3.26E-05
PA0002	*dnaN*	1.503995573	3.3E-05
PA0958	*oprD*	1.477949531	0.000152
PA4941	*hflC*	1.445458017	0.00016
PA1777	*oprF*	-1.26702446	3.16E-06
PA4764	*fur*	-1.33559422	2.74E-05
PA1801	*clpP*	-1.37125279	6.36E-08
PA2854	*PA2854*	-1.37442003	1.87E-05
PA4525	*pilA*	-1.39486324	1.77E-08
PA0769	*PA0769*	-1.41094659	0.000561
PA0547	*PA0547*	-1.41102286	4.89E-05
PA2023	*galU*	-1.4132511	8.22E-08
PA1609	*fabB*	-1.41513247	6.3E-07
PA4944	*hfq*	-1.41527849	4.38E-07
PA1681	*aroC*	-1.41530603	0.000389
PA5018	*msrA*	-1.41857425	4.12E-05
PA5013	*ilvE*	-1.4204145	2.2E-09
PA5489	*dsbA*	-1.42395223	1.03E-08
PA0838	*PA0838*	-1.42950248	0.00089
PA1821	*PA1821*	-1.43415074	5.47E-05
PA5371	*PA5371*	-1.4459322	0.000261
PA3257	*prc*	-1.44613299	9.54E-07
PA0653	*PA0653*	-1.45065772	1.83E-05
PA0068	*PA0068*	-1.45123163	0.000299
PA1754	*cysB*	-1.45182478	7.8E-07
PA5474	*PA5474*	-1.46720222	0.000145
PA4971	*aspP*	-1.48480492	8.14E-05
PA5174	*PA5174*	-1.48536685	3.87E-06
PA5067	*hisE*	-1.49203374	2.6E-06
PA4492	*PA4492*	-1.49998147	3.7E-06
PA5107	*blc*	-1.50327449	2.7E-05
PA0300	*spuD*	-1.50861222	7.52E-08
PA2531	*PA2531*	-1.51140622	7.8E-07
PA3199	*PA3199*	-1.51837314	1.09E-05
PA0265	*gabD*	-1.51884913	1.48E-07
PA5125	*ntrC*	-1.52112525	0.000567
PA5233	*PA5233*	-1.5280418	0.000513
PA5289	*PA5289*	-1.53108241	0.00016
PA5167	*PA5167*	-1.53318096	8.67E-05
PA4851	*PA4851*	-1.53459567	2.86E-06

PA0314	*PA0314*	-1.53570589	4.71E-07
PA1305	*PA1305*	-1.54224446	4.33E-07
PA3195	*gapA*	-1.55065314	3.93E-06
PA0962	*PA0962*	-1.55934108	0
PA0103a	*PA0103a*	-1.5663492	0.000437
PA1752	*PA1752*	-1.56863625	9.09E-06
PA0763	*mucA*	-1.57712286	0
PA0849	*trxB2*	-1.57793909	7.82E-08
PA2025	*gor*	-1.58109341	5E-09
PA2637	*nuoA*	-1.58192499	1.14E-08
PA3256	*PA3256*	-1.58592524	6.6E-05
PA4519	*speC*	-1.59180114	4.3E-09
PA4135	*PA4135*	-1.60187458	0.000249
PA3754	*PA3754*	-1.60265076	2.82E-05
PA3017	*PA3017*	-1.60383077	2.48E-09
PA4455	*PA4455*	-1.60389178	9.21E-10
PA0837	*slyD*	-1.60460855	0
PA4079	*PA4079*	-1.6106318	2.56E-08
PA2444	*glyA2*	-1.61128701	4.18E-08
PA4722	*PA4722*	-1.61139144	3.35E-06
PA0006	*PA0006*	-1.61536661	4.65E-05
PA4534	*PA4534*	-1.61929969	1.27E-06
PA0429	*PA0429*	-1.6206213	3.65E-12
PA5200	*amgR*	-1.62066058	4.97E-11
PA1829	*PA1829*	-1.62532453	0.000436
PA2707	*PA2707*	-1.626381	2.91E-08
PA4315	*mvaT*	-1.6325726	2.03E-08
PA3531	*bfrB*	-1.63380746	0
PA1775	*cmpX*	-1.63393146	4.45E-09
PA1769	*PA1769*	-1.63505142	3.87E-09
PA0667	*PA0667*	-1.64622719	1.34E-10
PA4537	*PA4537*	-1.65554339	6.24E-06
PA0655	*PA0655*	-1.65580741	6.05E-15
PA4922	*azu*	-1.65587858	0
PA2427	*PA2427*	-1.65799715	1.07E-13
PA2666	*PA2666*	-1.65905681	0.000447
PA5305	*PA5305*	-1.66223194	1.44E-08
PA4556	*pilE*	-1.66597618	3.66E-09
PA1249	*aprA*	-1.66872773	7.68E-11
PA0060	*PA0060*	-1.6715343	0.000674
PA5176	*PA5176*	-1.67396184	2.84E-10
PA5435	*PA5435*	-1.68368298	4.83E-08
PA2709	*cysK*	-1.6839406	1.86E-11

PA5447	*wbpZ*	-1.69088223	4.03E-05
PA4657	*PA4657*	-1.69100168	7.38E-10
PA5358	*ubiA*	-1.69364056	7.8E-11
PA2802	*PA2802*	-1.69600985	9.62E-08
PA5068	*tatA*	-1.69972869	8.73E-11
PA0376	*rpoH*	-1.70151624	0
PA3266	*capB*	-1.70890372	0
PA3784	*PA3784*	-1.70952276	1.46E-11
PA2754	*PA2754*	-1.71114631	5.29E-13
PA2860	*PA2860*	-1.7115361	4.15E-07
PA3201	*PA3201*	-1.7260436	2.82E-09
PA4523	*PA4523*	-1.72877198	1.05E-13
PA5347	*PA5347*	-1.73811043	7.86E-08
PA3697	*PA3697*	-1.73897786	7.03E-09
PA3170	*PA3170*	-1.74335471	1.19E-11
PA4180	*PA4180*	-1.74349676	1.63E-11
PA4798	*PA4798*	-1.74352259	5.9E-05
PA1029	*PA1029*	-1.74437271	0.000179
PA1933	*PA1933*	-1.74535882	6.86E-10
PA4787	*PA4787*	-1.75422607	1.41E-08
PA1812	*mltD*	-1.76067353	4.18E-13
PA5162	*rmlD*	-1.76540688	2.75E-11
PA0158	*PA0158*	-1.77831067	6.38E-05
PA5363	*PA5363*	-1.78042743	1.68E-12
PA4511	*PA4511*	-1.78441557	0.000302
PA5436	*PA5436*	-1.78594194	2.44E-09
PA4970	*PA4970*	-1.78686928	1.1E-11
PA4401	*PA4401*	-1.78761399	3.22E-07
PA3122	*PA3122*	-1.79121732	0.000696
PA0071	*PA0071*	-1.79759493	1.67E-10
PA2403	*PA2403*	-1.79777218	2.05E-12
PA5198	*PA5198*	-1.79848494	1.65E-08
PA4434	*PA4434*	-1.79892501	1.66E-09
PA2582	*PA2582*	-1.8053896	0
PA3533	*PA3533*	-1.80582523	0
PA0661	*PA0661*	-1.8132247	1.72E-10
PA1679	*PA1679*	-1.81741018	4.24E-15
PA3972	*PA3972*	-1.81816424	0.000228
PA1579	*PA1579*	-1.82277211	7.63E-20
PA2318	*PA2318*	-1.83076451	6.77E-05
PA1838	*cysI*	-1.83087362	1.9E-12
PA0167	*PA0167*	-1.8323862	6.57E-08
PA4701	*PA4701*	-1.83457764	6.74E-14

PA2856	*tesA*	-1.83523161	2.11E-06
PA5150	*PA5150*	-1.85122925	4.52E-17
PA2009	*hmgA*	-1.85141647	1.17E-08
PA4236	*katA*	-1.85458092	0
PA0312	*PA0312*	-1.8561705	2.86E-13
PA3584	*glpD*	-1.85848772	0
PA1741	*PA1741*	-1.86704779	1.16E-11
PA1813	*PA1813*	-1.86906729	3.04E-10
PA5206	*argE*	-1.87407381	1.42E-11
PA2824	*PA2824*	-1.87480883	3.85E-05
PA1096	*PA1096*	-1.87655665	0
PA3052	*PA3052*	-1.87737845	1.1E-10
PA1203	*PA1203*	-1.88003052	0.000842
PA3349	*PA3349*	-1.88383126	0
PA5165	*PA5165*	-1.8838556	0.000102
PA4951	*orn*	-1.8860684	2.17E-15
PA1756	*cysH*	-1.89190447	1.71E-08
PA3437	*folM*	-1.89499655	6.28E-07
PA4521	*PA4521*	-1.9033609	9.36E-07
PA0715	*PA0715*	-1.9033807	1.74E-09
PA1946	*rbsB*	-1.90639429	1.74E-06
PA2797	*PA2797*	-1.90657646	6.4E-17
PA0745	*PA0745*	-1.91028637	1.58E-08
PA3043	*PA3043*	-1.91044266	2.57E-10
PA2443	*sdaA*	-1.9131974	5.94E-16
PA4175	*piv*	-1.91392065	8.96E-21
PA1065	*PA1065*	-1.91561704	7.57E-05
PA1005	*PA1005*	-1.91714157	1.4E-15
PA0053	*PA0053*	-1.91771465	2.14E-11
PA3582	*glpK*	-1.92893925	6.29E-23
PA2896	*PA2896*	-1.93144825	5.41E-05
PA0706	*cat*	-1.93762415	1.89E-09
PA3345	*PA3345*	-1.93845988	0.000321
PA5267	*hcpB*	-1.95808254	4.78E-08
PA0565	*PA0565*	-1.96328821	2.93E-09
PA2685	*PA2685*	-1.9638614	1.32E-08
PA2960	*pilZ*	-1.96834104	1.95E-13
PA3815	*iscR*	-1.97206175	2.9E-21
PA3022	*PA3022*	-1.97620706	4.31E-16
PA2586	*gacA*	-1.97923268	9.47E-22
PA1306	*PA1306*	-1.97987761	7.12E-14
PA2756	*PA2756*	-1.98150209	3.98E-15
PA2718	*PA2718*	-1.98300067	9.93E-06

PA5313	*PA5313*	-1.98648437	2.52E-12
PA1610	*fabA*	-1.98666039	8.18E-22
PA5240	*trxA*	-1.99399004	0
PA1474	*PA1474*	-1.99846993	2.44E-15
PA3053	*PA3053*	-2.0017382	3E-05
PA1039	*PA1039*	-2.00300356	0.000181
PA3229	*PA3229*	-2.00369995	0.000207
PA0157	*PA0157*	-2.00391002	0.000128
PA5450	*wzt*	-2.00516555	2.64E-06
PA4604	*PA4604*	-2.00519779	6.57E-08
PA1247	*aprE*	-2.0076475	1.34E-07
PA5288	*glnK*	-2.01307147	0
PA1034	*PA1034*	-2.02006374	3.11E-05
PA3010	*PA3010*	-2.03122201	3.77E-21
PA2231	*pslA*	-2.03157768	6.74E-12
PA1246	*aprD*	-2.03465552	9.06E-11
PA1199	*PA1199*	-2.04100309	2.41E-21
PA1366	*PA1366*	-2.04300581	1.82E-11
PA3399	*PA3399*	-2.04753406	5.39E-06
PA5111	*gloA3*	-2.0498315	4.02E-12
PA4611	*PA4611*	-2.05015144	0
PA2008	*fahA*	-2.0516979	1.4E-09
PA0500	*bioB*	-2.05200588	2.46E-15
PA4954	*motA*	-2.05667773	1.11E-22
PA4026	*PA4026*	-2.06580076	1.99E-15
PA1478	*PA1478*	-2.06686506	1.19E-06
PA5119	*glnA*	-2.07039406	0
PA1248	*aprF*	-2.07869326	6.15E-05
PA4419	*ftsL*	-2.07915478	6.05E-09
PA1129	*PA1129*	-2.08232067	5.25E-07
PA0195	*pntAA*	-2.08689856	6E-11
PA2606	*PA2606*	-2.08928722	9.68E-14
PA2559a	*PA2559a*	-2.09310152	0.000258
PA3313	*PA3313*	-2.09457196	5.5E-21
PA0862	*PA0862*	-2.09606509	3.94E-20
PA4940	*PA4940*	-2.09842701	4.31E-13
PA2805	*PA2805*	-2.10035205	0
PA0055	*PA0055*	-2.10779797	4.9E-17
PA5091	*hutG*	-2.10966281	0.000206
PA2045	*PA2045*	-2.10989145	6.15E-13
PA0338	*PA0338*	-2.11602041	3.24E-05
PA4451	*PA4451*	-2.12073652	1.85E-20
PA0385	*PA0385*	-2.1226252	1.81E-05

PA4614	*mscL*	-2.13267538	0
PA1432	*lasI*	-2.13447399	5.54E-24
PA2609	*PA2609*	-2.13488673	1.42E-15
PA1331	*PA1331*	-2.13495105	0.000208
PA5423	*PA5423*	-2.13500497	9.78E-16
PA0820	*PA0820*	-2.137419	2.52E-10
PA0821	*PA0821*	-2.13786853	1.88E-06
PA1244	*PA1244*	-2.13861361	1.3E-24
PA4291	*PA4291*	-2.13933455	8.21E-23
PA1998	*dhcR*	-2.14050561	0.000345
PA1245	*PA1245*	-2.14309228	6.37E-28
PA0148	*PA0148*	-2.15113746	2.62E-15
PA0603	*PA0603*	-2.15412428	1.45E-09
PA2942.1	*PA2942.1*	-2.16198261	1.09E-17
PA2049	*PA2049*	-2.1626731	1.41E-10
PA1066	*PA1066*	-2.16364389	4.44E-06
PA3340	*PA3340*	-2.16838833	1.34E-06
PA3898	*PA3898*	-2.18577737	5.78E-07
PA2062	*PA2062*	-2.18667506	2.19E-06
PA4697	*PA4697*	-2.19088664	1.92E-18
PA1463	*PA1463*	-2.19239654	6.54E-25
PA1134	*PA1134*	-2.21522627	6.98E-07
PA0953	*PA0953*	-2.21627129	1.16E-12
PA5017	*PA5017*	-2.21995057	4.63E-24
PA1040	*PA1040*	-2.22196559	1.73E-07
PA4735	*PA4735*	-2.22229318	4.72E-25
PA3306	*PA3306*	-2.23099948	1.59E-10
PA3733	*PA3733*	-2.2314525	4.02E-12
PA2568	*PA2568*	-2.23735166	1.17E-12
PA3739	*PA3739*	-2.23823836	1.14E-05
PA3338	*PA3338*	-2.23945211	1.68E-07
PA3475	*pheC*	-2.24126888	6.78E-11
PA2195	*hcnC*	-2.24150248	3.73E-08
PA3856	*PA3856*	-2.24206414	3.24E-14
PA3455	*PA3455*	-2.24428939	8.26E-26
PA5161	*rmlB*	-2.25076697	1.47E-38
PA0792	*prpD*	-2.25660177	2.83E-11
PA0446	*PA0446*	-2.25838791	6.34E-12
PA4466	*PA4466*	-2.26046909	3.52E-14
PA5356	*glcC*	-2.26314791	0.000121
PA3576	*PA3576*	-2.26687751	1.85E-08
PA4463	*PA4463*	-2.26841869	0
PA4111	*PA4111*	-2.27014766	3.22E-11

PA4027	*PA4027*	-2.27482042	1.11E-05
PA2841	*PA2841*	-2.28178393	2.24E-08
PA0397	*PA0397*	-2.28390132	3.5E-08
PA1376	*aceK*	-2.28496198	2.22E-12
PA4421.1	*rnpB*	-2.2851161	0
PA1269	*PA1269*	-2.29670558	2.08E-12
PA4181	*PA4181*	-2.29793784	1.53E-05
PA3330	*PA3330*	-2.30235485	0.000232
PA2857	*PA2857*	-2.30411361	1.06E-06
PA3785	*PA3785*	-2.30961351	1.78E-18
PA5446	*PA5446*	-2.31400588	0
PA3615	*PA3615*	-2.3147084	6.82E-30
PA1760	*PA1760*	-2.32783508	6.86E-07
PA1469	*PA1469*	-2.33001052	4.01E-05
PA1092	*fliC*	-2.34669116	0
PA4116	*bphO*	-2.34888056	1.58E-11
PA1544	*anr*	-2.3498956	0
PA3412	*PA3412*	-2.35079672	0.000384
PA5153	*PA5153*	-2.35527624	2.24E-25
PA2620	*clpA*	-2.37599988	0
PA2747a	*PA2747a*	-2.38193111	1.2E-05
PA3857	*pcs*	-2.39078965	2.62E-23
PA2007	*maiA*	-2.39182421	3.01E-20
PA5522	*PA5522*	-2.39394441	5.49E-06
PA4391	*PA4391*	-2.39399859	4.71E-06
PA3190	*PA3190*	-2.39418406	3.31E-25
PA1123	*PA1123*	-2.39906819	1.72E-18
PA2827	*PA2827*	-2.40060413	4.75E-29
PA3602	*PA3602*	-2.40397048	3.53E-27
PA3819	*PA3819*	-2.40577874	8.97E-38
PA3786	*PA3786*	-2.4139915	1.83E-21
PA4535	*PA4535*	-2.42779974	5.48E-20
PA4713	*PA4713*	-2.43424437	0.000113
PA3239	*PA3239*	-2.43544318	3.58E-22
PA1473	*PA1473*	-2.43950426	8.76E-07
PA0954	*PA0954*	-2.44158027	1.79E-19
PA4017	*PA4017*	-2.44381485	3.53E-30
PA3796	*PA3796*	-2.44396926	1.87E-12
PA2749	*endA*	-2.44690262	5.63E-09
PA1325	*PA1325*	-2.45612263	8.93E-06
PA3681	*PA3681*	-2.4581377	2.49E-05
PA2781	*PA2781*	-2.45934889	0.000772
PA5261	*algR*	-2.47274849	2.72E-40

PA0952	*PA0952*	-2.4731516	1.81E-31
PA5255	*algQ*	-2.47325121	0
PA3436	*PA3436*	-2.47770147	6.94E-06
PA3352	*PA3352*	-2.47870201	0
PA5449	*wbpX*	-2.48216964	1.72E-08
PA2694	*PA2694*	-2.48702709	6.96E-10
PA3973	*PA3973*	-2.4893909	6.78E-16
PA2193	*hcnA*	-2.5006741	2.42E-06
PA5526	*PA5526*	-2.50364553	8.41E-18
PA3788	*PA3788*	-2.50745051	2.51E-10
PA1120	*tpbB*	-2.50836825	1.67E-09
PA5378	*PA5378*	-2.50913633	4.64E-19
PA4884	*PA4884*	-2.51130666	1.19E-18
PA5445	*PA5445*	-2.51200305	7.89E-11
PA3851	*PA3851*	-2.51432147	1.04E-29
PA1736	*PA1736*	-2.51753594	4.85E-05
PA0827	*PA0827*	-2.52980616	0.000114
PA3295	*PA3295*	-2.53519547	6.09E-30
PA2279	*arsC*	-2.53879686	2E-07
PA5451	*wzm*	-2.54128394	3.13E-10
PA1124	*dgt*	-2.54701027	1.94E-17
PA0784	*PA0784*	-2.54911922	3.2E-29
PA1623	*PA1623*	-2.55103644	1.12E-33
PA3272	*PA3272*	-2.55314322	0.000472
PA3463	*PA3463*	-2.55964837	9.85E-06
PA1401	*PA1401*	-2.56214521	0.00073
PA1817	*PA1817*	-2.56595574	1.94E-22
PA1994	*PA1994*	-2.56674164	1.03E-07
PA2751	*PA2751*	-2.57021313	0.000547
PA3142	*PA3142*	-2.57033672	2.31E-15
PA2404	*PA2404*	-2.58043766	7.46E-36
PA4024	*eutB*	-2.58458066	0.000147
PA1995	*PA1995*	-2.58681559	1.35E-09
PA2491	*PA2491*	-2.5890104	1.39E-33
PA4498	*PA4498*	-2.58914653	1.88E-25
PA3844	*PA3844*	-2.5953217	3.49E-27
PA4536	*PA4536*	-2.60162475	5.64E-08
PA2623	*icd*	-2.60767674	0
PA5452	*wbpW*	-2.60961742	3.03E-15
PA4712	*PA4712*	-2.61038525	2.31E-06
PA5461	*PA5461*	-2.61184482	0
PA5105	*hutC*	-2.61338805	4.08E-22
PA4522	*ampD*	-2.62126135	1.49E-18

PA3753	*PA3753*	-2.62227088	1.47E-19
PA1603	*PA1603*	-2.62414531	5.39E-15
PA1850	*PA1850*	-2.62443048	2.95E-05
PA2773	*PA2773*	-2.62630148	0.000566
PA4793	*PA4793*	-2.63224024	1.42E-29
PA2026	*PA2026*	-2.63525508	2.06E-09
PA4205	*mexG*	-2.64014249	3.64E-07
PA5030	*PA5030*	-2.64316002	6.82E-39
PA0905	*rsmA*	-2.64497775	0
PA4366	*sodB*	-2.64750212	0
PA4392	*PA4392*	-2.64951091	4.01E-06
PA5163	*rmlA*	-2.65544355	0
PA0830	*PA0830*	-2.66658702	1.21E-06
PA5253	*algP*	-2.66659147	0
PA3687	*ppc*	-2.67368819	1.13E-21
PA0851	*PA0851*	-2.6880425	3.97E-15
PA3098	*xcpW*	-2.68866503	7.42E-22
PA5285	*PA5285*	-2.68869042	9.76E-55
PA4677	*PA4677*	-2.69195352	1.51E-08
PA2105	*PA2105*	-2.69552228	4.84E-10
PA1119	*yfiB*	-2.6957655	2.17E-25
PA4775	*PA4775*	-2.69745573	0.000337
PA1879	*PA1879*	-2.70426731	1.22E-09
PA0114	*senC*	-2.7133933	7.75E-27
PA2659	*PA2659*	-2.71534016	8.85E-19
PA2121	*PA2121*	-2.71652908	2.12E-05
PA4636a	*PA4636a*	-2.72072447	8.69E-06
PA0704	*PA0704*	-2.72089956	1.55E-11
PA0424	*mexR*	-2.72240032	6.31E-37
PA3300	*fadD2*	-2.72758137	9.36E-10
PA4335	*PA4335*	-2.73294015	3.25E-10
PA0604	*PA0604*	-2.73320225	7.55E-11
PA3855	*PA3855*	-2.77213414	7.84E-19
PA2831	*PA2831*	-2.78058719	9.45E-31
PA3966	*PA3966*	-2.788447	4.84E-26
PA5360	*phoB*	-2.79259356	1.99E-30
PA0345	*PA0345*	-2.7952586	0.000971
PA4773	*PA4773*	-2.7982977	5.2E-12
PA3329	*PA3329*	-2.80477214	2.66E-06
PA1852	*PA1852*	-2.80484313	0
PA0846	*PA0846*	-2.81314534	2.49E-27
PA0793	*PA0793*	-2.81340111	5.49E-15
PA0344	*PA0344*	-2.81378519	1.31E-09

PA4633	*PA4633*	-2.82166804	4.95E-43
PA0996	*pqsA*	-2.82957234	0.000827
PA5520	*PA5520*	-2.83125723	4.08E-17
PA5124	*ntrB*	-2.83751269	2.86E-28
PA1358	*PA1358*	-2.84152848	7.53E-24
PA3123	*PA3123*	-2.8427246	1.34E-21
PA4108	*PA4108*	-2.84302955	1.76E-20
PA1991	*PA1991*	-2.84366064	0.000482
PA2280	*PA2280*	-2.846625	2.82E-08
PA4183	*PA4183*	-2.84996466	6.16E-11
PA2864	*PA2864*	-2.85464851	3.57E-37
PA0741	*PA0741*	-2.85785343	0.000426
PA2301	*PA2301*	-2.8672172	4.03E-11
PA1181	*PA1181*	-2.87684354	2.36E-08
PA1776	*sigX*	-2.88060055	8.01E-75
PA3333	*fabH2*	-2.88400508	1.23E-08
PA4147	*acoR*	-2.88843315	7.76E-07
PA0737	*PA0737*	-2.89429026	4.85E-07
PA4025	*PA4025*	-2.89646155	0.000731
PA2723	*PA2723*	-2.90023606	5.75E-10
PA5329	*PA5329*	-2.9014183	1.81E-27
PA4041	*PA4041*	-2.90226556	4.87E-06
PA1127	*PA1127*	-2.90808781	5.36E-14
PA4309	*pctA*	-2.91005614	2.05E-44
PA5020	*PA5020*	-2.91084594	4.02E-08
PA2430	*PA2430*	-2.92380187	6.32E-05
PA3238	*PA3238*	-2.92749991	3.08E-30
PA0095	*PA0095*	-2.92891167	9.11E-15
PA4641	*PA4641*	-2.93039935	6.38E-30
PA3331	*PA3331*	-2.93166182	6.85E-13
PA4827	*PA4827*	-2.93602068	1.23E-09
PA3957	*PA3957*	-2.93634962	4.19E-11
PA0436	*PA0436*	-2.93915136	3.35E-38
PA1095	*PA1095*	-2.94333256	0
PA1112.1	*PA1112.1*	-2.94390053	0
PA1523	*xdhB*	-2.95759769	4.65E-05
PA0543	*PA0543*	-2.95795811	1.78E-05
PA1402	*PA1402*	-2.95873696	0.000543
algZ	*algZ*	-2.9601252	7.29E-30
PA2927	*PA2927*	-2.96812863	1.7E-09
PA1672	*PA1672*	-2.97274459	1.24E-08
PA0132	*PA0132*	-2.97370243	2.17E-08
PA2633	*PA2633*	-2.97768339	2.63E-18

PA4033	*PA4033*	-2.99363545	6.1E-05
PA0460	*PA0460*	-2.99764259	7.43E-40
PA2276	*PA2276*	-3.01085581	0.000762
PA3186	*oprB*	-3.02524282	3.85E-17
PA1943	*PA1943*	-3.02851586	4.54E-12
PA3787	*PA3787*	-3.03369268	9.81E-25
PA0490	*PA0490*	-3.03531415	9.94E-11
PA2682	*PA2682*	-3.03838491	7.55E-06
PA3906	*PA3906*	-3.04292601	3.18E-14
PA4774	*PA4774*	-3.05033317	0.000109
PA5527	*PA5527*	-3.05433381	1.54E-65
PA1033	*PA1033*	-3.0552618	5.31E-41
PA0731	*PA0731*	-3.05526761	9.25E-25
PA2668	*PA2668*	-3.0570864	4.63E-24
PA4040	*PA4040*	-3.0642453	2.43E-05
PA0871	*phhB*	-3.06617286	5.58E-85
PA2949	*PA2949*	-3.06972887	2.25E-16
PA0794	*PA0794*	-3.07537043	1.81E-17
PA4914	*PA4914*	-3.08363219	0.000289
PA4382	*PA4382*	-3.09447152	1.21E-06
PA2608	*PA2608*	-3.09479322	1.92E-22
PA2549	*PA2549*	-3.09899923	1.1E-13
PA1437	*PA1437*	-3.11046047	1.1E-06
PA3430	*PA3430*	-3.11171078	4.38E-22
PA2605	*PA2605*	-3.11353721	6.62E-39
PA5249	*PA5249*	-3.11602605	1.26E-20
PA5454	*rmd*	-3.11791671	4.1E-36
PA4182	*PA4182*	-3.11925944	8.36E-29
PA0380	*PA0380*	-3.12297397	3.09E-19
PA1814	*PA1814*	-3.12632502	1.54E-41
PA4495	*PA4495*	-3.1266775	2.11E-65
PA0959	*PA0959*	-3.13320422	3.57E-37
PA4955	*PA4955*	-3.13427975	5.82E-21
PA3096	*xcpY*	-3.13768583	2.53E-31
PA4345	*PA4345*	-3.15188331	2.08E-31
PA4927	*PA4927*	-3.16422133	2.11E-08
PA3217	*cyaB*	-3.16455071	1.44E-27
PA3097	*xcpX*	-3.16865095	3.6E-25
PA2344	*mtlZ*	-3.18982692	1.38E-09
PA0540	*PA0540*	-3.1907363	6.73E-10
PA5164	*rmlC*	-3.20126229	4.93E-66
PA4994	*PA4994*	-3.21711667	8.49E-05
PA0364	*PA0364*	-3.22325209	4.56E-05

PA5104	*PA5104*	-3.24409846	1.17E-14
PA0099	*PA0099*	-3.24536312	1.01E-06
PA4717	*PA4717*	-3.25062637	2.19E-56
PA1121	*yfiR*	-3.26942803	1.22E-15
PA3908	*PA3908*	-3.27425694	1.87E-40
PA3867	*PA3867*	-3.27698037	1.33E-07
PA2764	*PA2764*	-3.27989434	7.16E-45
PA2244	*pslN*	-3.290501	6.5E-06
PA3079	*PA3079*	-3.29057194	3.32E-20
PA2814	*PA2814*	-3.29336373	4.6E-09
PA3919	*PA3919*	-3.29925379	2.32E-55
PA5060	*phaF*	-3.30345954	0
PA0810	*PA0810*	-3.31879613	6.54E-12
PA2358	*PA2358*	-3.31999977	0.000453
PA2883	*PA2883*	-3.32907248	1.51E-21
PA3327	*PA3327*	-3.33801042	3.91E-10
PA3351	*flgM*	-3.34497805	0
PA5108	*PA5108*	-3.34612988	2.72E-61
PA1342	*PA1342*	-3.35047498	0
PA4346	*PA4346*	-3.35486611	0.000145
PA3221	*csaA*	-3.35670776	1.57E-30
PA3373	*PA3373*	-3.36235051	3.88E-30
PA3779	*PA3779*	-3.37945769	1.54E-07
PA2893	*atuH*	-3.38652338	0.000769
PA5028	*PA5028*	-3.39021499	5.42E-63
PA1472	*PA1472*	-3.39037747	1.3E-07
PA1753	*PA1753*	-3.39226288	5.34E-38
PA0960	*PA0960*	-3.39385129	8.07E-17
PA1189	*PA1189*	-3.39969168	1.98E-09
PA4378	*inaA*	-3.40907918	6.95E-50
PA2897	*PA2897*	-3.40979011	2.7E-109
PA1545	*PA1545*	-3.42722312	1.71E-50
PA1430	*lasR*	-3.43688184	9.5E-74
PA3254	*PA3254*	-3.44403419	2.66E-10
PA4889	*PA4889*	-3.44671088	0.000306
PA1328	*PA1328*	-3.45137385	1.81E-24
PA1737	*PA1737*	-3.4557708	2.98E-13
PA2830	*htpX*	-3.45657229	8.36E-90
PA0505	*PA0505*	-3.45840278	1.1E-127
PA3682	*PA3682*	-3.46195373	3.99E-16
PA1945	*PA1945*	-3.46384965	7.39E-07
PA4364	*PA4364*	-3.46450954	3.02E-16
PA4456	*PA4456*	-3.48119836	1.21E-97

PA5213	*gcvP1*	-3.48196231	4.51E-14
PA2489	*PA2489*	-3.49579345	7.49E-10
PA3459	*PA3459*	-3.51885442	3.5E-78
PA4926	*PA4926*	-3.5283189	9.01E-08
PA4326	*PA4326*	-3.53045572	5.69E-41
PA3080	*PA3080*	-3.53689779	5.66E-63
PA2989	*PA2989*	-3.53758501	3.23E-22
PA3825	*PA3825*	-3.54175978	1.52E-12
PA1951	*PA1951*	-3.54901906	2.08E-13
PA0572	*PA0572*	-3.56748882	4.4E-103
PA1198	*PA1198*	-3.59676285	1.5E-154
PA2750	*PA2750*	-3.61848657	1.07E-43
PA0853	*PA0853*	-3.6264027	2.58E-17
PA5521	*PA5521*	-3.62768913	1.83E-77
PA0422	*PA0422*	-3.62814473	5.44E-72
PA1423	*bdlA*	-3.62950043	3.08E-22
PA5073	*PA5073*	-3.63178994	1.79E-68
PA1592	*PA1592*	-3.6417129	0
PA1191	*PA1191*	-3.64200191	1.09E-16
PA4605	*PA4605*	-3.65384341	2.87E-45
PA0804	*PA0804*	-3.66162836	9.43E-52
PA0734	*PA0734*	-3.66952237	6.69E-12
PA4875	*PA4875*	-3.67831663	4.95E-27
PA0462	*PA0462*	-3.68068532	6.55E-60
PA5453	*gmd*	-3.69672877	1E-69
PA5528	*PA5528*	-3.70090178	0
PA4635	*PA4635*	-3.70404304	2.33E-06
PA0839	*PA0839*	-3.71643086	7.03E-20
PA0777	*PA0777*	-3.71751654	1.78E-16
PA0988	*PA0988*	-3.719233	1.51E-56
PA3089	*PA3089*	-3.72646465	3.67E-10
PA4034	*aqpZ*	-3.7335744	5.21E-11
PA2373	*PA2373*	-3.73625729	5.21E-11
PA1887	*PA1887*	-3.74895132	1.8E-05
PA4384	*PA4384*	-3.76434967	6.08E-28
PA0689	*PA0689*	-3.77771868	5.18E-33
PA3756	*PA3756*	-3.78514569	8.4E-104
PA3846	*PA3846*	-3.79827878	1.83E-76
PA3426	*PA3426*	-3.80170234	3.83E-26
PA2716	*PA2716*	-3.80369497	4.17E-09
PA4444	*mltB1*	-3.80755345	1.97E-47
PA1408	*PA1408*	-3.8122352	1.11E-08
PA5276	*lppL*	-3.8231069	4.62E-26

PA0557	*PA0557*	-3.82317962	3.54E-14
PA3581	*glpF*	-3.83636123	1.23E-92
PA2511	*PA2511*	-3.83751746	7.06E-09
PA0315	*PA0315*	-3.83950215	0
PA0452	*PA0452*	-3.85392404	6.7E-06
PA1939	*PA1939*	-3.86890129	2.79E-88
PA4591	*PA4591*	-3.88872078	2.2E-07
PA3740	*PA3740*	-3.91257947	1.03E-62
PA0507	*PA0507*	-3.92155292	9.55E-07
PA3095	*xcpZ*	-3.9220883	1.5E-26
PA2915	*PA2915*	-3.92596982	1.39E-44
PA5361	*phoR*	-3.93497275	2.89E-16
PA0029	*PA0029*	-3.94731077	0.000969
PA5462	*PA5462*	-3.95198066	3.66E-36
PA3711	*PA3711*	-3.95529666	5.26E-25
PA3572	*PA3572*	-3.95545052	4.76E-27
PA1895	*PA1895*	-3.95841797	4.15E-06
PA1749	*PA1749*	-3.9604792	4.1E-122
PA3103	*xcpR*	-3.97712863	6.9E-109
PA3684	*PA3684*	-3.98823061	5.95E-25
PA5106	*PA5106*	-3.98849776	1.11E-20
PA3332	*PA3332*	-3.98940351	1.16E-27
PA1202	*PA1202*	-3.99968377	3E-152
PA4606	*PA4606*	-4.00038824	4E-111
PA0269	*PA0269*	-4.00561001	1.53E-06
PA2788	*PA2788*	-4.01706352	2.2E-119
PA3907	*PA3907*	-4.02326525	2.1E-23
PA4327	*PA4327*	-4.04158055	2E-15
PA4681	*PA4681*	-4.04576366	8.17E-05
PA2780	*PA2780*	-4.04776793	1.2E-11
PA5234	*PA5234*	-4.07450143	2.6E-47
PA3102	*xcpS*	-4.07938218	8.87E-69
PA4782	*PA4782*	-4.08480848	3.09E-35
PA2796	*tal*	-4.10803228	4.8E-154
PA3905	*PA3905*	-4.11354711	6.49E-13
PA2378	*PA2378*	-4.11622158	8.87E-32
PA0854	*fumC2*	-4.11729443	2.09E-63
PA4190	*pqsL*	-4.11979588	9.35E-65
PA3100	*xcpU*	-4.13139908	3.7E-106
PA3140	*PA3140*	-4.13321105	1.5E-09
PA4397	*panE*	-4.15324057	4.72E-12
PA2376	*PA2376*	-4.155419	0.000591
PA3236	*PA3236*	-4.17308381	3.04E-05

PA3105	*xcpQ*	-4.18057142	2.3E-114
PA1872	*PA1872*	-4.2178736	1.24E-60
PA0855	*PA0855*	-4.23730181	5.03E-19
PA2879	*PA2879*	-4.23779859	1.29E-40
PA2702	*PA2702*	-4.24433899	1.09E-23
PA2755a	*PA2755a*	-4.25033087	5.4E-99
PA5380	*gbdR*	-4.2576463	3.44E-22
PA3690	*PA3690*	-4.25901663	6.58E-41
PA3120	*leuD*	-4.27035635	4.1E-108
PA3526	*PA3526*	-4.27162731	1.18E-66
PA3965	*PA3965*	-4.27559053	1.22E-94
PA0788	*PA0788*	-4.27797009	2.18E-07
PA3031	*PA3031*	-4.27837971	0
PA2114	*PA2114*	-4.28408586	0.000963
PA0012	*PA0012*	-4.29843557	5.59E-24
PA2189	*PA2189*	-4.30152643	4.1E-06
PA2678	*PA2678*	-4.30477034	1.85E-05
PA5421	*fdhA*	-4.31452984	1.85E-05
PA0982	*PA0982*	-4.34759556	2.08E-33
PA1122	*PA1122*	-4.35188523	1.4E-51
PA1159	*PA1159*	-4.35567328	1.66E-97
PA4379	*PA4379*	-4.3620076	3.6E-100
PA2786	*PA2786*	-4.36884333	6E-17
PA4206	*mexH*	-4.39249366	6.29E-13
PA0732	*PA0732*	-4.39802416	4.92E-35
PA0473	*PA0473*	-4.41569501	1.04E-31
PA0447	*gcdH*	-4.42323287	7.73E-54
PA0366	*PA0366*	-4.42721895	4.24E-32
PA0524	*norB*	-4.43144239	0.000358
PA4010	*PA4010*	-4.4389084	7.3E-115
PA2047	*PA2047*	-4.44430422	3.9E-17
PA3261	*PA3261*	-4.44447795	6.73E-70
PA3039	*PA3039*	-4.45504308	3.5E-07
PA4499	*PA4499*	-4.47299112	2.83E-48
PA1345	*PA1345*	-4.47720115	3.37E-07
PA3496	*PA3496*	-4.47857549	2.28E-14
PA4176	*ppiC2*	-4.49060424	1.9E-113
PA3551	*algA*	-4.50242523	4.4E-30
PA0805	*PA0805*	-4.50535824	3.7E-157
PA3969	*PA3969*	-4.50621288	2.55E-86
PA2708	*PA2708*	-4.51040289	1.21E-07
PA3104	*xcpP*	-4.51398003	1.12E-85
PA2878	*PA2878*	-4.5314847	1.05E-18

PA1617	*PA1617*	-4.54060999	5.48E-40
PA5103	*PA5103*	-4.56511209	1.23E-24
PA3392	*nosZ*	-4.57126352	7.23E-08
PA1641	*PA1641*	-4.58683579	4.82E-09
PA3689	*PA3689*	-4.60023336	5.82E-87
PA0575	*PA0575*	-4.62583759	8.79E-20
PA3945	*PA3945*	-4.62826932	9.12E-67
PA4362	*PA4362*	-4.65797451	2E-29
PA1250	*aprI*	-4.69605011	5.65E-52
PA2621	*PA2621*	-4.69645167	9.73E-85
PA2787	*cpg2*	-4.69794947	6.49E-35
PA0365	*PA0365*	-4.70375427	8.3E-16
PA2555	*PA2555*	-4.71087488	1.33E-09
PA0861	*PA0861*	-4.71921394	3.07E-59
PA2477	*PA2477*	-4.74154717	2.58E-05
PA0049	*PA0049*	-4.753193	1.07E-10
PA1106	*PA1106*	-4.7551554	9.4E-103
PA2327	*PA2327*	-4.76674746	2.84E-67
PA1944	*PA1944*	-4.76817107	5.49E-47
PA1136	*PA1136*	-4.77098161	1.4E-12
PA2076	*PA2076*	-4.7743402	1.74E-20
PA5191	*PA5191*	-4.78576237	2.99E-36
PA5306	*PA5306*	-4.79102543	0.000513
PA2329	*PA2329*	-4.799753	6.65E-88
PA2985	*PA2985*	-4.80155686	8.73E-83
PA5180	*PA5180*	-4.82057284	9.91E-72
PA4117	*bphP*	-4.84268881	5.6E-122
PA4207	*mexI*	-4.85126152	8.64E-12
PA4336	*PA4336*	-4.8654287	5.09E-41
PA4733	*acsB*	-4.86878988	3.11E-26
PA0270	*PA0270*	-4.87117247	6.68E-14
PA1118	*PA1118*	-4.90407603	1.67E-65
PA3041	*PA3041*	-4.92337243	2E-87
PA3929	*cioB*	-4.92400384	6.5E-109
PA3307	*PA3307*	-4.93533913	5.2E-70
PA2799	*PA2799*	-4.94428281	2.52E-44
PA2938	*PA2938*	-4.98099612	3.98E-05
PA4365	*PA4365*	-5.01793506	4.51E-19
PA3852	*PA3852*	-5.05325783	4.2E-129
PA3534	*PA3534*	-5.06006833	1.39E-08
PA0840	*PA0840*	-5.06217399	3.03E-59
PA1774	*crfX*	-5.08079154	1.2E-114
PA1761	*PA1761*	-5.08703125	1.28E-59

PA1167	*PA1167*	-5.14783146	2.27E-39
PA0459	*PA0459*	-5.1484268	4.77E-91
PA4993	*PA4993*	-5.18481744	2.04E-88
PA3858	*PA3858*	-5.19086085	9.36E-86
PA3795	*PA3795*	-5.19695575	5.7E-117
PA2306	*ambA*	-5.20602843	1.03E-92
PA4714	*PA4714*	-5.20639362	1.8E-179
PA2679	*PA2679*	-5.22390226	4.96E-28
PA2504	*PA2504*	-5.22891033	1.49E-35
PA3427	*PA3427*	-5.24651087	9.54E-35
PA3180	*PA3180*	-5.31779587	4.9E-101
PA4830	*PA4830*	-5.33190585	3.19E-57
PA0951a	*PA0951a*	-5.33265428	3.2E-126
PA0795	*prpC*	-5.33781602	8.9E-124
PA3253	*PA3253*	-5.33800114	2.22E-10
PA3094	*PA3094*	-5.3488219	8.1E-143
PA5182	*PA5182*	-5.38518335	3.5E-100
PA2887	*atuB*	-5.39908859	2.69E-06
PA3252	*PA3252*	-5.39923538	2.22E-10
PA4325	*PA4325*	-5.42670469	8.3E-135
PA2791	*PA2791*	-5.44183756	7.74E-16
PA3833	*PA3833*	-5.47719778	5.96E-84
PA2328	*PA2328*	-5.47732481	1.49E-85
PA3188	*PA3188*	-5.53032533	2.11E-09
PA2374	*PA2374*	-5.53364097	3.78E-11
PA1190	*PA1190*	-5.53399544	9.28E-42
PA1149	*PA1149*	-5.5469989	2.65E-05
PA2111	*PA2111*	-5.55429854	9.14E-09
PA1762	*PA1762*	-5.55566174	1.79E-14
PA3099	*xcpV*	-5.55713615	1.23E-36
PA3963a	*PA3963a*	-5.55762577	5.21E-05
PA5297	*poxB*	-5.5748815	2.93E-40
PA1112	*PA1112*	-5.6070434	3.1E-117
PA2415	*PA2415*	-5.66546091	4.86E-10
PA4312	*PA4312*	-5.67597535	5.9E-103
PA2816	*PA2816*	-5.69295969	9.92E-68
PA5546	*PA5546*	-5.69697982	2E-186
PA5061	*PA5061*	-5.70009284	2.1E-187
PA0558	*PA0558*	-5.70098766	9.5E-102
PA2330	*PA2330*	-5.7064636	5.1E-108
PA3372	*PA3372*	-5.72129091	3.73E-60
PA2779	*PA2779*	-5.72151411	7.77E-54
PA4828	*PA4828*	-5.72781975	2.63E-20

PA3273	*PA3273*	-5.73503304	8.37E-10
PA0180	*cttP*	-5.75874064	4.87E-80
PA0173	*PA0173*	-5.78638969	0.000401
PA0676	*vreR*	-5.79702374	2.52E-42
PA3568	*PA3568*	-5.88730761	1.55E-16
PA1575	*PA1575*	-5.88742864	2.58E-76
PA1894	*PA1894*	-5.93616385	9.86E-06
PA1003	*mvfR*	-5.9372887	1.3E-139
PA4829	*lpd3*	-5.94781736	9.94E-22
PA4289	*PA4289*	-5.95839158	2.67E-30
PA4915	*PA4915*	-5.9649764	3.72E-44
PA0690	*PA0690*	-5.97874217	1.77E-20
PA0039	*PA0039*	-5.98218376	3.5E-199
PA5098	*hutH*	-6.05923596	5.64E-08
PA1111	*PA1111*	-6.08213194	4.29E-05
PA4786	*PA4786*	-6.10255313	2.28E-30
PA5437	*PA5437*	-6.11200301	2.2E-157
PA4112	*PA4112*	-6.12079428	4.04E-43
PA3570	*mmsA*	-6.15067	1.48E-77
PA3920	*PA3920*	-6.18311997	4.29E-27
PA4497	*PA4497*	-6.19037782	5.76E-72
PA1048	*PA1048*	-6.19645931	3.7E-175
PA1989	*pqqE*	-6.21044132	1.32E-18
PA5545	*PA5545*	-6.22896856	1.04E-97
PA0743	*PA0743*	-6.22925357	1.69E-30
PA2345	*PA2345*	-6.23010709	2.97E-74
PA2277	*arsR*	-6.23775508	4.81E-28
PA3928	*PA3928*	-6.27277224	2.2E-134
PA4624	*PA4624*	-6.28624761	1.04E-19
PA1471	*PA1471*	-6.34340326	6.9E-130
PA2338	*PA2338*	-6.35071763	4.25E-07
PA2550	*PA2550*	-6.35299305	2.54E-26
PA0050	*PA0050*	-6.35705443	1.7E-110
PA4785	*PA4785*	-6.36488672	1.39E-25
PA0788a	*PA0788a*	-6.36762756	6.14E-42
PA0306a	*PA0306a*	-6.37030868	5.95E-36
PA0143	*nuh*	-6.38210691	1E-144
PA2580	*PA2580*	-6.42923736	1.11E-20
PA1107	*PA1107*	-6.47859077	1.81E-18
PA2475	*PA2475*	-6.50163763	2.23E-29
PA3346	*PA3346*	-6.51756123	2.1E-129
PA2490	*PA2490*	-6.54046033	1.62E-22
PA3930	*cioA*	-6.54651106	8.5E-146

PA0797	*PA0797*	-6.55660627	4.64E-81
PA0742	*PA0742*	-6.62580455	1.3E-16
PA0847	*PA0847*	-6.63189318	3.14E-17
PA0035	*trpA*	-6.63887394	1.1E-113
PA1990	*pqqH*	-6.64487912	7.84E-08
PA2771	*PA2771*	-6.65712679	1.52E-93
PA2593	*qteE*	-6.66010176	7.93E-19
PA5227.1	*ssrS*	-6.66235688	5.7E-227
PA0806	*PA0806*	-6.72014009	1.38E-05
PA3101	*xcpT*	-6.73243729	4.31E-31
PA2790	*PA2790*	-6.73578539	6.6E-104
PA5395	*PA5395*	-6.76694464	1.61E-14
PA2339	*PA2339*	-6.78993253	0.000105
PA4654	*PA4654*	-6.83693312	0.000806
PA5424	*PA5424*	-6.83863302	4.8E-177
PA1348	*PA1348*	-6.87092618	2.55E-09
PA1643a	*PA1643a*	-6.87648946	7.2E-102
PA0701	*PA0701*	-6.8803249	4.45E-08
PA1650	*PA1650*	-6.9060892	1.15E-41
PA4496	*PA4496*	-6.91799175	3.6E-135
PA4338	*PA4338*	-6.91981907	1.6E-27
PA0983	*PA0983*	-6.92937396	2.48E-36
PA2379	*PA2379*	-6.94069289	7.79E-79
PA2360	*PA2360*	-6.95539907	5.76E-18
PA4608	*PA4608*	-6.98558978	6.3E-165
PA4874	*PA4874*	-6.98714365	2.5E-160
PA2560	*PA2560*	-6.99582954	8.6E-134
PA3462	*PA3462*	-7.01059806	1.1E-10
PA3040	*PA3040*	-7.08535752	6.1E-165
PA1988	*pqqD*	-7.1097789	7.34E-25
PA0007	*PA0007*	-7.13457323	1.05E-49
PA1287	*PA1287*	-7.17249151	8.1E-162
PA4650	*PA4650*	-7.18448341	7.16E-29
PA0256	*PA0256*	-7.18465107	3.5E-52
PA2177	*PA2177*	-7.18674962	2.42E-19
PA1333	*PA1333*	-7.18788306	3.35E-19
PA3023	*PA3023*	-7.20898817	1.83E-59
PA3042	*PA3042*	-7.23026493	5.9E-109
PA1648	*PA1648*	-7.23344795	2.95E-24
PA5181.1	*PA5181.1*	-7.25827509	5.59E-53
PA5181	*PA5181*	-7.2679939	1.73E-82
PA0656	*PA0656*	-7.31280325	9.3E-139
PA2562	*PA2562*	-7.3156872	1.2E-145

PA3216	*PA3216*	-7.31682227	7.63E-20
PA3311	*PA3311*	-7.33307372	7.4E-26
PA2364	*PA2364*	-7.34131057	1.3E-27
PA0179	*PA0179*	-7.37461341	1.6E-136
PA0286	*desA*	-7.43518067	2.3E-63
PA2361	*PA2361*	-7.43575515	1.16E-13
PA2553	*PA2553*	-7.44596499	2.08E-80
PA2618	*PA2618*	-7.4600081	1.6E-114
PA4300	*tadC*	-7.47622387	8.77E-17
PA0803	*PA0803*	-7.51898991	3.36E-97
PA0213	*PA0213*	-7.52739318	9.5E-15
PA0796	*prpB*	-7.52918825	1.3E-146
PA3472	*PA3472*	-7.52946722	2.7E-142
PA0216	*PA0216*	-7.5347629	1.89E-12
PA1731	*PA1731*	-7.59724655	3.14E-55
PA2807	*PA2807*	-7.60067016	7.86E-05
PA5271	*PA5271*	-7.60750892	6.5E-120
PA2920	*PA2920*	-7.62150129	2.3E-112
PA2414	*PA2414*	-7.65843919	1.24E-37
PA1878	*PA1878*	-7.6771429	8.4E-101
PA1882	*PA1882*	-7.68806415	2.43E-11
PA4351	*PA4351*	-7.69315376	1.12E-89
PA4293	*pprA*	-7.78407816	6.7E-19
PA0329	*PA0329*	-7.7853917	3E-185
PA3121	*leuC*	-7.79650592	1.6E-158
PA5099	*PA5099*	-7.80635747	1.41E-10
PA4212	*phzC1*	-7.80981468	7.29E-36
PA1963	*PA1963*	-7.86416575	1.16E-25
PA3540	*algD*	-7.8707083	1.7E-128
PA2869	*PA2869*	-7.88376769	1.18E-36
PA4127	*hpcG*	-7.89104225	4.28E-08
PA0798	*pmtA*	-7.89711123	1.01E-45
PA0918	*PA0918*	-7.90389385	3.41E-73
PA2302	*ambE*	-7.95650915	7.7E-62
PA4301	*tadB*	-8.0857842	4.58E-14
PA0208	*mdcA*	-8.12566064	2.01E-29
PA3009	*PA3009*	-8.12671105	6E-122
PA1258	*PA1258*	-8.2773451	1.82E-06
PA2746a	*PA2746a*	-8.28003205	5.4E-111
PA1873	*PA1873*	-8.32407718	2.18E-20
PA2919	*PA2919*	-8.33300477	2.92E-21
PA0215	*PA0215*	-8.34473633	1.35E-34
PA2862	*lipA*	-8.38604734	2.47E-19

PA4084	*cupB3*	-8.49541491	5.36E-05
PA2340	*PA2340*	-8.54506505	2.7E-12
PA2815	*PA2815*	-8.59280065	5.3E-129
PA2569	*PA2569*	-8.62879786	6.92E-43
PA2701	*PA2701*	-8.63589104	8.66E-09
PA4888	*desB*	-8.63693826	4.04E-09
PA3629	*adhC*	-8.73432735	7.4E-154
PA0288	*gpuA*	-8.79108147	2.7E-12
PA4653	*PA4653*	-8.80689338	3.63E-29
PA3289	*PA3289*	-8.81303837	4.07E-98
PA4500	*PA4500*	-8.81800598	9.2E-150
PA4015	*PA4015*	-8.84877004	2.9E-178
PA0028	*PA0028*	-8.85137688	1.1E-89
PA4132	*PA4132*	-8.85926858	2.4E-131
PA2506	*PA2506*	-8.88570154	0.000255
PA2070	*PA2070*	-8.91594906	1.23E-06
PA4682	*PA4682*	-8.9181512	1.11E-31
PA1166	*PA1166*	-8.95530163	2.1E-81
PA0484	*PA0484*	-8.99941972	2.5E-159
PA2024	*PA2024*	-9.02313523	1.04E-64
PA2604	*PA2604*	-9.03206941	0
PA3691	*PA3691*	-9.07728004	4.71E-48
PA5366	*pstB*	-9.13687797	5.7E-151
PA0451	*PA0451*	-9.14851703	1.8E-27
PA5101	*PA5101*	-9.22514771	3.2E-19
PA2416	*treA*	-9.30164387	7.09E-22
PA0826.2	*ssrA*	-9.31676448	0
PA5219	*PA5219*	-9.32999045	3.7E-59
PA0674	*vreA*	-9.33522668	9.84E-85
PA2331	*PA2331*	-9.39105966	1.8E-174
PA2175	*PA2175*	-9.4428462	3.24E-15
PA4208	*opmD*	-9.45472766	3.62E-26
PA2108	*PA2108*	-9.46209483	1.77E-15
PA4108a	*PA4108a*	-9.50165709	9.17E-11
PA2448	*PA2448*	-9.50340709	5.47E-37
PA3460	*PA3460*	-9.5320032	3.8E-126
PA4144	*PA4144*	-9.54086965	2.02E-07
PA0290	*PA0290*	-9.57151176	7.3E-103
PA1837a	*PA1837a*	-9.59827941	7.71E-88
PA2305	*ambB*	-9.59984555	1.79E-60
PA2563	*PA2563*	-9.67098261	7.01E-66
PA3038	*PA3038*	-9.67617782	3.36E-72
PA0274	*PA0274*	-9.68319346	4.07E-07

PA1987	*pqqC*	-9.69844648	3.44E-19
PA1562	*acnA*	-9.70278044	3.7E-160
PA0027	*PA0027*	-9.72150402	3.5E-123
PA1901	*phzC2*	-9.74049486	1.64E-45
PA2485	*PA2485*	-9.75260616	1.5E-50
PA3205	*PA3205*	-9.78954564	4.6E-160
PA0178	*PA0178*	-9.81718602	3.67E-70
PA2370	*PA2370*	-9.9079727	1.05E-27
PA0036	*trpB*	-9.91114005	1.3E-119
PA4778	*cueR*	-9.96399174	3.4E-116
PA0687	*PA0687*	-10.0796242	1.42E-09
PA2544	*PA2544*	-10.1207897	2.7E-159
PA2071	*fusA2*	-10.1304087	3E-144
PA0112	*PA0112*	-10.1550904	1.25E-10
PA3554	*arnA*	-10.214782	8.1E-70
PA0523	*norC*	-10.2376522	4.69E-17
PA3921	*PA3921*	-10.2380959	1.32E-84
PA3628	*PA3628*	-10.2468301	2.5E-133
PA3712	*PA3712*	-10.2763167	6.7E-149
PA1431	*rsaL*	-10.2772645	4.75E-67
PA2168	*PA2168*	-10.329473	7.54E-18
PA4652	*PA4652*	-10.3452452	5.61E-16
PA1254	*PA1254*	-10.346892	6.51E-23
PA2188	*PA2188*	-10.3882908	6.63E-15
PA2571	*PA2571*	-10.3933542	2.36E-72
PA2552	*PA2552*	-10.4284142	1.79E-77
PA0108	*coIII*	-10.4460881	5.55E-88
PA3904	*PA3904*	-10.5176681	4.9E-129
PA3792	*leuA*	-10.5364868	0
PA2363	*PA2363*	-10.5943831	9.2E-54
PA5359	*PA5359*	-10.806456	3.3E-191
PA4022	*PA4022*	-10.8199862	2.31E-68
PA0673	*PA0673*	-10.8307507	1.3E-176
PA2721	*PA2721*	-10.8378287	2.62E-76
PA2065	*pcoA*	-10.8684606	5.84E-12
PA4607	*PA4607*	-10.958344	1.4E-184
PA5212	*PA5212*	-11.0741061	3.2E-171
PA3415	*PA3415*	-11.1079725	5.26E-41
PA0483	*PA0483*	-11.1883323	8.2E-118
PA1135	*PA1135*	-11.2220698	1.8E-112
PA3535	*PA3535*	-11.2450445	4.05E-50
PA4134	*PA4134*	-11.2828704	2.7E-139
PA1728	*PA1728*	-11.3620416	4.8E-164

PA3251	*PA3251*	-11.4004044	2.61E-17
PA3692	*lptF*	-11.4205517	1.22E-47
PA0585	*PA0585*	-11.5845575	2.08E-32
PA0866	*aroP2*	-11.5850649	1.49E-80
PA4788	*PA4788*	-11.6026387	2.47E-35
PA1732	*PA1732*	-11.6074936	7E-111
PA2156	*PA2156*	-11.6569285	4.14E-14
PA5365	*phoU*	-11.7848118	3.2E-139
PA2808	*ptrA*	-11.8124263	3.65E-25
PA2918	*PA2918*	-11.8522977	9.1E-125
PA2381	*PA2381*	-11.8638539	1.9E-136
PA0776	*PA0776*	-11.9837596	3.49E-39
PA0921	*PA0921*	-12.0184925	7.7E-121
PA1932	*PA1932*	-12.0477752	7.12E-33
PA0038	*PA0038*	-12.0740715	2.6E-180
PA2486	*PA2486*	-12.1051987	5.32E-42
PA4877	*PA4877*	-12.146174	6E-110
PA3733a	*PA3733a*	-12.1960644	6.7E-158
PA1730	*PA1730*	-12.2260329	5.17E-76
PA2573	*PA2573*	-12.236445	6.2E-153
PA2303	*ambD*	-12.2438238	1.82E-92
PA5057	*phaD*	-12.2700487	3E-121
PA3325	*PA3325*	-12.3023817	1.11E-52
PA2722	*PA2722*	-12.4142613	2.08E-50
PA1745	*PA1745*	-12.4620908	4.16E-85
PA5348	*PA5348*	-12.5545322	2.9E-230
PA4635a	*PA4635a*	-12.5711715	5.93E-34
PA0176	*aer2*	-12.6123797	2.2E-113
PA1860	*PA1860*	-12.6292913	1.41E-78
PA1252	*PA1252*	-12.634626	2.17E-56
PA1931	*PA1931*	-12.6544402	4.37E-59
PA5368	*pstC*	-12.7049381	1.6E-122
PA0841	*PA0841*	-12.7105565	5.21E-93
PA3477	*rhlR*	-12.7215296	0
PA2419	*PA2419*	-12.7306659	4.71E-64
PA2176	*PA2176*	-12.8813195	3.62E-22
PA3362	*PA3362*	-12.9567828	1.2E-106
PA2304	*ambC*	-13.0023776	2.43E-93
PA2750a	*PA2750a*	-13.0695746	1.7E-162
PA4214	*phzE1*	-13.0979427	5.55E-42
PA1733	*PA1733*	-13.2397625	3.6E-123
PA1255	*PA1255*	-13.2571455	2.05E-21
PA4303	*tadZ*	-13.2891524	1.01E-25

PA2554	*PA2554*	-13.3692523	2.05E-52
PA0355	*pfpI*	-13.4086854	2.8E-122
PA3417	*PA3417*	-13.4579711	2.66E-40
PA4781	*PA4781*	-13.6755632	1.79E-75
PA4171	*PA4171*	-13.6908949	3.5E-118
PA1986	*pqqB*	-13.7340081	2.15E-60
PA3461	*PA3461*	-13.8289746	1.6E-107
PA2574	*alkB1*	-13.8600439	3.38E-18
PA4305	*rcpC*	-13.8799262	1.97E-81
PA2423	*PA2423*	-13.9685861	2.5E-139
PA0211	*mdcD*	-14.0592582	4.32E-72
PA0212	*mdcE*	-14.062925	2.97E-61
PA1172	*napC*	-14.1927461	1.3E-129
PA3374	*PA3374*	-14.2664706	7.6E-140
PA1881	*PA1881*	-14.2778255	3.5E-111
PA5367	*pstA*	-14.3607485	7.8E-160
PA2587	*pqsH*	-14.3927422	3.3E-157
PA3347	*PA3347*	-14.4185759	6.4E-236
PA3585	*glpM*	-14.4531637	3.6E-134
PA0685	*PA0685*	-14.4603329	3.23E-34
PA4573	*PA4573*	-14.521291	2.8E-139
PA1351	*PA1351*	-14.6141505	2.99E-15
PA2017	*PA2017*	-14.6436179	1.5E-110
PA2591	*PA2591*	-14.6453388	8.9E-109
PA3368	*PA3368*	-14.6983192	1.44E-67
PA2341	*PA2341*	-14.7876498	6.59E-17
PA0567	*PA0567*	-14.7951887	9.6E-118
PA1256	*PA1256*	-14.8051988	2.98E-26
PA2622	*cspD*	-14.8734118	2.8E-236
PA2145	*PA2145*	-14.9363743	1.27E-22
PA0026	*plcB*	-14.9834194	9.8E-163
PA2747	*PA2747*	-14.985193	5.62E-36
PA2072	*PA2072*	-14.9916028	1.01E-34
PA4304	*rcpA*	-15.0462089	3.48E-42
PA4172	*PA4172*	-15.1768274	9.8E-33
PA2179	*PA2179*	-15.2886764	1.08E-25
PA2592	*PA2592*	-15.5036673	4.2E-158
PA5178	*PA5178*	-15.5300013	0
PA4625	*PA4625*	-15.6572623	7.6E-80
PA4726.11	*crcZ*	-15.6606857	0
PA0175	*PA0175*	-15.7219559	1.88E-38
PA4702	*PA4702*	-15.7804428	4.7E-145
PA4311	*PA4311*	-15.9043343	3.9E-107

PA0174	*PA0174*	-15.9489108	1E-10
PA1041	*PA1041*	-15.9500478	4.6E-167
PA5220	*PA5220*	-16.0945382	3.6E-165
PA2162	*PA2162*	-16.1459484	2.71E-52
PA0587	*PA0587*	-16.1964815	2.1E-146
PA4590	*pra*	-16.2831212	4.31E-83
PA4143	*PA4143*	-16.2966275	1.58E-20
PA2144	*glgP*	-16.3378362	1.8E-100
PA4880	*PA4880*	-16.4969696	1.1E-131
PA0696	*PA0696*	-16.5991508	1.33E-31
PA2572	*PA2572*	-16.6269832	5E-110
PA0586	*PA0586*	-16.6409357	6.8E-132
PA2250	*lpdV*	-16.6755698	2.1E-126
PA1905	*phzG2*	-16.7153536	7.44E-91
PA1880	*PA1880*	-16.8301022	2.9E-123
PA4876	*osmE*	-16.9421136	2.4E-213
PA0675	*vreI*	-16.9650968	5.64E-50
PA1356	*PA1356*	-16.990994	5.94E-40
PA0698	*PA0698*	-17.0500326	5.29E-41
PA2371	*PA2371*	-17.3217254	5.29E-52
PA2699	*PA2699*	-17.334406	1.18E-27
PA1323	*PA1323*	-17.3516632	9.9E-176
PA0981	*PA0981*	-17.3820443	4.1E-120
PA2362	*PA2362*	-17.3939694	2.1E-13
PA1354	*PA1354*	-17.443464	2.9E-70
PA2109	*PA2109*	-17.4943844	1.42E-18
PA2185	*katN*	-17.5037151	3.17E-67
PA1898	*qscR*	-17.5062669	2.54E-37
PA1259	*PA1259*	-17.5719614	8.57E-39
PA3326	*PA3326*	-17.7069854	0
PA4925	*PA4925*	-17.7084149	5.95E-91
PA5059	*PA5059*	-17.7101016	3.4E-121
PA0684	*PA0684*	-17.9943221	8.97E-23
PA3416	*PA3416*	-18.0139902	6.73E-52
PA1176	*napF*	-18.0888952	4.36E-70
PA3623	*PA3623*	-18.212054	4.8E-295
PA2048	*PA2048*	-18.2351931	5.7E-105
PA4142	*PA4142*	-18.2487205	3.71E-73
PA0677	*PA0677*	-18.4813492	1.78E-87
PA0865	*hpd*	-18.5099094	1.6E-141
PA3369	*PA3369*	-18.7566228	7.2E-156
PA3688	*PA3688*	-18.8400553	1.7E-168
PA2107	*PA2107*	-18.8859974	3.62E-10

PA3049	*rmf*	-18.933321	0
PA0588	*PA0588*	-19.0312298	1.53E-68
PA4215	*phzF1*	-19.1521057	2.07E-46
PA1903	*phzE2*	-19.1598249	8.46E-48
PA0681	*PA0681*	-19.2411436	9.61E-73
PA0103	*PA0103*	-19.2930087	1.6E-132
PA3363	*amiR*	-19.3727672	6.2E-141
PA1904	*phzF2*	-19.4380781	1.38E-51
PA0105	*coxB*	-19.444576	6.7E-133
PA4913	*PA4913*	-19.4552893	1.88E-72
PA0110	*PA0110*	-19.4878463	6.25E-22
PA0109	*PA0109*	-19.4920091	7.2E-111
PA1899	*phzA2*	-19.5365415	2.74E-35
PA4294	*PA4294*	-19.6394019	5.57E-81
PA0102	*PA0102*	-19.6488115	0
PA2636	*PA2636*	-19.9084082	1.2E-38
PA2247	*bkdA1*	-20.2916817	1.6E-143
PA0683	*PA0683*	-20.4129288	6.5E-44
PA3274	*PA3274*	-20.578773	5.2E-143
PA2369	*PA2369*	-20.7181329	8.56E-65
PA2135	*PA2135*	-20.7520381	5.25E-11
PA1350	*PA1350*	-20.7862793	1.96E-58
PA0209	*PA0209*	-20.7936112	5.45E-21
PA3418	*ldh*	-21.0234751	3.6E-150
PA4297	*tadG*	-21.7523019	1.01E-86
PA4290	*PA4290*	-21.7886588	2.45E-63
PA4377	*PA4377*	-21.9284293	1.6E-190
PA0699	*PA0699*	-21.9403239	8.79E-31
PA0679	*PA0679*	-21.9694756	3.8E-106
PA2167	*PA2167*	-22.247947	6.77E-49
PA2248	*bkdA2*	-22.3645609	2.5E-122
PA2249	*bkdB*	-22.5656746	1.5E-110
PA2157	*PA2157*	-22.6009697	2.83E-41
PA2181	*PA2181*	-22.6666779	7.67E-22
PA3371	*PA3371*	-23.0115969	4.9E-194
PA3569	*mmsB*	-23.0614761	2.15E-79
PA0107	*PA0107*	-23.0911182	4.15E-62
PA4295	*fppA*	-23.2526031	1.84E-11
PA3364	*amiC*	-23.5235165	1.51E-88
PA1053	*PA1053*	-23.6970109	0
PA5100	*hutU*	-23.8809004	2E-119
PA4302	*tadA*	-24.0024601	4.49E-40
PA4296	*pprB*	-24.0135136	7.1E-170

PA2746	*PA2746*	-24.086409	1E-173
PA2588	*PA2588*	-24.1845013	2.5E-129
PA4649	*PA4649*	-24.3090248	1.19E-60
PA0177	*PA0177*	-24.723513	1.1E-79
PA2142	*PA2142*	-24.8034586	5.79E-46
PA1902	*phzD2*	-24.8073082	7.26E-64
PA5056	*phaC1*	-24.8901972	1.7E-145
PA0059	*osmC*	-25.0593529	3.4E-159
PA3234	*PA3234*	-25.1585727	3.4E-128
PA0692	*PA0692*	-25.2626294	5.21E-33
PA4299	*tadD*	-25.6786854	6.52E-29
PA1173	*napB*	-25.9985633	2.33E-77
PA0887	*acsA*	-26.0632234	2.2E-146
PA3734	*PA3734*	-26.4438848	4.6E-120
PA0106	*coxA*	-26.4631844	1.43E-92
PA0214	*PA0214*	-26.6865191	7.59E-30
PA3622	*rpoS*	-26.7863477	0
PA2139	*PA2139*	-26.8228417	1.05E-34
PA4128	*PA4128*	-26.8646157	1.54E-36
PA2138	*PA2138*	-27.0472455	5.29E-58
PA2022	*PA2022*	-27.0589835	5.3E-119
PA0347	*glpQ*	-27.0623997	1.9E-70
PA1130	*rhlC*	-27.4854415	6.4E-171
PA2418	*PA2418*	-27.517184	6.64E-44
PA3476	*rhlI*	-27.8460691	3.4E-186
PA4078	*PA4078*	-27.8522836	1.79E-81
PA0680	*PA0680*	-28.3327616	5.86E-41
PA2160	*PA2160*	-28.974149	7.4E-152
PA0695	*PA0695*	-29.0393261	1E-32
PA2763a	*PA2763a*	-29.1734499	1.8E-99
PA3986	*PA3986*	-29.2494231	9.6E-155
PA0978	*PA0978*	-29.3896848	2E-117
PA3723	*PA3723*	-29.7410781	2.2E-169
PA2173a	*PA2173a*	-29.8294721	1.8E-120
PA3032	*snr1*	-29.8687377	7.15E-92
PA5481	*PA5481*	-29.8897119	3.4E-191
PA4131	*PA4131*	-29.9355487	1.3E-153
PA2192	*PA2192*	-30.1041251	8.13E-22
PA2152	*PA2152*	-30.707606	1.57E-83
PA2190	*PA2190*	-30.8671131	7.1E-194
PA0691	*PA0691*	-30.8717647	1.95E-32
PA1870	*PA1870*	-31.3410611	3.9E-104
PA2187	*PA2187*	-31.4589572	8E-39

PA2565	*PA2565*	-31.6309138	1.64E-89
PA1175	*napD*	-32.4638659	1.1E-125
PA2564	*PA2564*	-32.6890862	2.4E-205
PA1930	*PA1930*	-33.2320423	1.5E-102
PA3370	*PA3370*	-33.4437209	8.85E-63
PA1606	*PA1606*	-33.6633883	2.2E-152
PA2375	*PA2375*	-33.7410652	4.06E-95
PA2112	*PA2112*	-34.6501333	1.12E-07
PA1324	*PA1324*	-34.790315	1.3E-259
PA1869	*PA1869*	-35.3842207	7.1E-206
PA0686	*PA0686*	-35.6496455	5.06E-36
PA1180	*phoQ*	-35.9455442	1.3E-175
PA3519	*PA3519*	-37.1221101	2.33E-16
PA2880	*PA2880*	-37.2350436	3.1E-130
PA0852	*cbpD*	-37.303509	0
PA2153	*glgB*	-37.4472328	5.43E-79
PA2164	*PA2164*	-38.1501311	8.9E-104
PA2137	*PA2137*	-38.481675	6.02E-70
PA1344	*PA1344*	-38.5038716	1.4E-214
PA2178	*PA2178*	-38.8655341	2.34E-38
PA2165	*PA2165*	-39.2057588	2.6E-133
PA5058	*phaC2*	-39.3118334	1.3E-148
PA1404	*PA1404*	-39.6732444	5.9E-144
PA3924	*PA3924*	-39.9894817	3.2E-113
PA2882	*PA2882*	-40.0601282	4.3E-125
PA0730	*PA0730*	-40.151262	3E-226
PA5482	*PA5482*	-40.4463082	1.97E-14
PA4350	*PA4350*	-41.0595002	4.3E-306
PA3366	*amiE*	-41.4965995	2.98E-65
PA4209	*phzM*	-41.5791151	1.8E-163
PA0111	*PA0111*	-41.9751957	7.81E-32
PA0682	*PA0682*	-42.4936184	1.07E-38
PA2433	*PA2433*	-42.7536171	4.8E-213
PA1177	*napE*	-42.9173196	3.5E-124
PA2154	*PA2154*	-43.0474149	3.75E-24
PA3586	*PA3586*	-43.4237726	3E-138
PA4130	*PA4130*	-43.8943332	7.4E-112
PA0844	*plcH*	-44.3303444	5.65E-94
PA1784	*PA1784*	-44.4090911	2.9E-123
PA2367	*PA2367*	-45.2044556	6.8E-122
PA1327	*PA1327*	-45.387794	1E-193
PA2155	*PA2155*	-45.438941	1.34E-28
PA0843	*plcR*	-45.5669513	1.4E-139

PA1914	*PA1914*	-47.1491713	3.04E-96
PA3553	*arnC*	-47.4112859	8.1E-182
PA2173	*PA2173*	-47.6364385	5.7E-131
PA5369	*pstS*	-47.9848386	0
PA2589	*PA2589*	-48.4113275	9.87E-47
PA0688	*PA0688*	-49.1220283	2.2E-197
PA2158	*PA2158*	-49.7796336	1.2E-80
PA0740	*sdsA1*	-49.8569551	2.2E-119
PA4133	*PA4133*	-50.1455121	3.1E-155
PA0188	*PA0188*	-50.880821	1.13E-69
PA1876	*PA1876*	-51.3318484	4.5E-140
PA2066	*PA2066*	-51.6874152	1.7E-127
PA2366	*PA2366*	-52.2226147	1.7E-155
PA2030	*PA2030*	-52.2853764	7.5E-186
PA1174	*napA*	-52.5699512	1.6E-106
PA3250	*PA3250*	-52.6729066	3E-150
PA2151	*PA2151*	-52.8127554	6.1E-116
PA0842	*PA0842*	-52.8469284	4.1E-120
PA3375	*PA3375*	-52.8811372	2.22E-84
PA4703	*PA4703*	-54.2486353	2E-117
PA2150	*PA2150*	-54.8183664	9.89E-61
PA0697	*PA0697*	-55.351953	2.51E-47
PA3231	*PA3231*	-56.014864	5.2E-103
PA4213	*phzD1*	-56.0463037	1.38E-67
PA3235	*PA3235*	-56.6053945	6.4E-200
PA4651	*PA4651*	-56.6060098	7.3E-114
PA4739	*PA4739*	-56.9056109	0
PA1900	*phzB2*	-56.9809444	4.7E-127
PA3377	*PA3377*	-57.0583958	1.9E-141
PA0210	*mdcC*	-58.4370082	4.55E-44
PA2163	*PA2163*	-59.6136875	3.6E-62
PA3451	*PA3451*	-59.8637367	5.6E-182
PA2803	*PA2803*	-60.3225468	4.6E-117
PA1871	*lasA*	-61.2806563	1.4E-220
PA2696	*PA2696*	-61.3306899	8.6E-112
PA2937	*PA2937*	-61.6413619	3.2E-133
PA1875	*PA1875*	-62.0698499	1.5E-168
PA2717	*cpo*	-62.8892151	8.9E-167
PA2881	*PA2881*	-64.2268583	4E-159
PA3384	*phnC*	-64.686766	1.4E-112
PA4141	*PA4141*	-64.82751	0
PA2172	*PA2172*	-65.2111507	5.2E-134
PA2428	*PA2428*	-65.8301087	7.7E-148

PA2182	*PA2182*	-66.3216189	3.53E-77
PA0693	*exbB2*	-67.318961	2.87E-42
PA3365	*PA3365*	-67.7834537	7.2E-131
PA2365	*PA2365*	-68.7371383	2.3E-157
PA2148	*PA2148*	-69.7054898	2.17E-52
PA3923	*PA3923*	-69.7761702	9.5E-182
PA0051	*phzH*	-71.1812334	2.6E-176
PA1179	*phoP*	-71.9870493	0
PA1349	*PA1349*	-72.373771	1.04E-49
PA1353	*PA1353*	-72.6642204	7.5E-140
PA4129	*PA4129*	-72.9614707	2.8E-130
PA0122	*PA0122*	-73.7455422	0
PA5440a	*PA5440a*	-73.914279	0
PA2184	*PA2184*	-74.5303975	1.7E-149
PA2804	*PA2804*	-75.1577208	1.9E-194
PA2566	*PA2566*	-75.3125662	2.6E-181
PA3378	*PA3378*	-77.3946661	7.9E-161
PA3296	*phoA*	-80.3374925	2.3E-174
PA1874	*PA1874*	-80.9894933	5.3E-235
PA3376	*PA3376*	-88.3015717	1.2E-114
PA4738	*PA4738*	-88.8019403	1.8E-210
PA3382	*phnE*	-89.6099322	5.6E-173
PA0052	*PA0052*	-91.2248618	3E-165
PA3922	*PA3922*	-92.4233749	6.5E-73
PA1877	*PA1877*	-96.8617272	5.9E-118
PA2368	*PA2368*	-97.2321239	2.56E-37
PA2159	*PA2159*	-100.10995	6E-144
PA3381	*PA3381*	-101.437707	2.3E-122
PA3380	*PA3380*	-101.762421	4.4E-157
PA2174	*PA2174*	-102.053478	1.7E-212
PA4211	*phzB1*	-107.969811	1.9E-111
PA2300	*chiC*	-109.430884	1.2E-225
PA3478	*rhlB*	-110.288241	0
PA2147	*katE*	-110.68223	1.1E-147
PA5460	*PA5460*	-116.384764	9.7E-227
PA2939	*PA2939*	-120.000803	0
PA2166	*PA2166*	-120.695742	0
PA3319	*plcN*	-124.635885	4.4E-216
PA1131	*PA1131*	-127.938569	2E-113
PA0678	*PA0678*	-129.410347	2.86E-73
PA2067	*PA2067*	-133.53102	2.4E-175
PA2027	*PA2027*	-134.134205	0
PA3361	*lecB*	-136.085973	1.33E-81

PA0187	*PA0187*	-141.73338	6.7E-120
PA2134	*PA2134*	-144.547219	5.5E-172
PA3520	*PA3520*	-146.627754	4.5E-271
PA4648	*PA4648*	-146.735832	1.5E-167
PA3552	*arnB*	-147.440079	0
PA1571	*PA1571*	-151.107834	0
PA3724	*lasB*	-154.297024	0
PA2031	*PA2031*	-163.224616	4.1E-127
PA3280	*oprO*	-163.967998	0
PA2635	*PA2635*	-165.754942	1.5E-291
PA2068	*PA2068*	-170.057393	4.2E-185
PA0979	*PA0979*	-177.993265	1.7E-184
PA1178	*oprH*	-178.614928	0
PA4306	*flp*	-183.668609	3.3E-280
PA1343	*PA1343*	-205.189047	2.1E-139
PA3219	*PA3219*	-206.537471	2.7E-172
PA3383	*PA3383*	-206.887194	3.03E-77
PA3910	*eddA*	-208.653961	3.47E-80
PA1260	*PA1260*	-220.212167	2.4E-169
PA2069	*PA2069*	-225.929314	1.2E-267
PA3909	*eddB*	-227.185866	0
PA2171	*PA2171*	-253.423828	3.7E-211
PA2146	*PA2146*	-257.92613	0
PA3479	*rhlA*	-258.133699	0
PA2143	*PA2143*	-277.058589	7.4E-118
PA2180	*PA2180*	-292.746255	3.69E-83
PA4637a	*PA4637a*	-345.842164	0
PA3379	*PA3379*	-400.722857	7.8E-154
PA2169	*PA2169*	-413.182363	1.1E-138
PA2570	*lecA*	-513.51683	0

<u>Table S2:</u> Changes in bacterial gene expression associated with outer membrane vesicle localization in response to infection measured by RNA-seq. Fold changes greater than 1 indicate that the gene was up-regulated in *P. aeruginosa* infected mice compared to non-infected mice. Fold changes inferior to -1 indicate that the gene was down-regulated in *P. aeruginosa* infected mice compared to non-infected mice. This table includes genes which products were predicted or experimentally shown to be associated with outer membrane vesicles.

Locus	Gene	Fold change	P-value
PA4489	PA4489	2.37	9.97E-10
PA4491	PA4491	1.78	0.000278
PA3821	secD	5.05	1.8E-159
PA5560	atpB	3.26	1.71E-96
PA0768	lepB	3.41	3.33E-36
PA5479	gltP	2.80	1.42E-32
PA3984	lnt	2.79	6.99E-14
PA4997	msbA	1.79	2.13E-08
PA4700	mrcB	2.02	2.44E-07
PA5291	PA5291	1.64	0.000225
PA4461	PA4461	1.91	1.54E-14
PA5568	PA5568	6.37	3.5E-195
PA3641	PA3641	10.97	8.3E-175
PA1805	ppiD	5.16	3.8E-171
PA3729	PA3729	6.20	3.9E-124
PA2973	PA2973	3.50	5.7E-60
PA3147	wbpJ	3.52	6.69E-43
PA5558	atpF	8.34	1.07E-42
PA5232	PA5232	2.87	4.25E-42
PA3822	PA3822	2.40	2.31E-41
PA4292	PA4292	2.77	7.46E-32
PA4431	PA4431	2.27	6.9E-31
PA1584	sdhB	2.15	2.36E-30
PA4430	PA4430	2.14	1.37E-22
PA4003	pbpA	3.31	1.2E-21
PA5304	dadA	2.36	1.84E-18
PA5052	PA5052	2.58	9.32E-18
PA3875	narG	3.24	1.44E-15
PA1583	sdhA	1.80	2.71E-14
PA4957	psd	1.98	4.17E-13
PA3878	narX	2.66	4.08E-11
PA0074	ppkA	3.13	3.25E-10
PA2290	gcd	1.82	1.97E-09
PA1767	PA1767	1.80	4.85E-09
PA0077	tssM1	2.59	9.73E-09

PA0375	ftsX	1.85	2.87E-08
PA2983	PA2983	1.80	9.08E-08
PA0913	mgtE	1.93	2.73E-06
PA5065	ubiB	1.81	0.000143
PA3838	PA3838	2.25	0.000174
PA0080	tssJ1	2.07	0.00022
PA4385	groEL	9.38	9.66E-60
PA4266	fusA1	5.76	8.07E-37
PA0963	aspS	2.31	5E-27
PA3001	PA3001	1.94	3.74E-25
PA0411	pilJ	3.89	9.4E-135
PA5553	atpC	8.51	3.1E-212
PA1556	ccoO2	22.06	4.4E-206
PA5016	aceF	6.60	2E-174
PA0141	PA0141	8.11	1.3E-170
PA1596	htpG	10.35	1.9E-169
PA0782	putA	5.84	3.1E-116
PA5015	aceE	3.53	7.83E-95
PA4771	lldD	14.31	2.31E-91
PA2976	rne	4.95	4.4E-90
PA4568	rplU	3.51	1.78E-87
PA4267	rpsG	5.71	1.98E-80
PA2840	PA2840	3.81	8.62E-80
PA2741	rplT	3.53	1.01E-78
PA1585	sucA	3.20	1.11E-78
PA1800	tig	2.87	3.82E-77
PA3452	mqoA	3.41	4.2E-76
PA4262	rplD	12.85	4.73E-76
PA1586	sucB	3.36	8.91E-76
PA4251	rplE	10.61	1.18E-74
PA4432	rpsI	3.66	2.49E-72
PA0618	PA0618	6.69	7.28E-69
PA4274	rplK	3.49	6.99E-68
PA4269	rpoC	6.46	3.01E-67
PA4273	rplA	6.19	1.27E-65
PA4253	rplN	5.31	1.86E-64
PA1589	sucD	5.27	1.24E-62
PA3656	rpsB	7.96	3.87E-57
PA4257	rpsC	10.95	1.43E-55
PA5555	atpG	10.03	8.07E-53
PA4745	nusA	2.54	3.44E-51
PA4238	rpoA	7.46	2.64E-50
PA4240	rpsK	6.95	1.66E-49

PA4761	dnaK	10.94	1.76E-47
PA4239	rpsD	6.38	5.33E-47
PA5556	atpA	7.59	1.88E-46
PA5554	atpD	6.84	1.7E-44
PA4237	rplQ	6.33	1.95E-43
PA4671	PA4671	5.60	2.48E-39
PA1746	PA1746	3.06	1.57E-38
PA4670	prs	2.18	1.83E-34
PA3014	faoA	2.25	9.11E-34
PA3159	wbpA	2.28	6.46E-33
PA5128	secB	2.29	8.74E-31
PA4270	rpoB	4.91	1.24E-26
PA1553	ccoO1	2.25	1.64E-24
PA3150	wbpG	2.49	5.12E-22
PA5005	PA5005	2.12	1.22E-20
PA3309	PA3309	1.86	3.14E-20
PA4352	PA4352	2.16	2.38E-17
PA4582	PA4582	3.44	4.33E-16
PA0576	rpoD	1.73	7.39E-15
PA4481	mreB	1.83	1.09E-10
PA4756	carB	1.76	1.34E-09
PA1787	acnB	1.56	3.79E-07
PA4333	PA4333	1.67	5.17E-07
PA2999	nqrA	1.71	1.8E-06
PA2540	PA2540	2.54	7.3E-06
PA4941	hflC	1.45	0.00016
PA2994	nqrF	3.16	1.38E-35
PA0620	PA0620	3.59	1.7E-36
PA0985	pyoS5	5.21	9.19E-35
PA1150	pys2	2.08	7.12E-11
PA1080	flgE	1.83	3.06E-09
PA1086	flgK	2.08	1.66E-12
PA3262	PA3262	1.69	8.25E-12
PA3901	fecA	40.02	4.7E-164
PA0165	PA0165	17.14	3.5E-144
PA4675	PA4675	7.00	2.59E-58
PA1288	PA1288	2.91	2.28E-44
PA1271	PA1271	6.61	1.84E-23
PA3268	PA3268	7.00	1.26E-20
PA0162	opdC	5.98	1.93E-20
PA2462	PA2462	5.11	1.12E-15
PA3800	PA3800	3.83	7.9E-137
PA0625	PA0625	8.63	9.7E-108

PA3082	gbt	6.25	2.11E-58
PA1011	PA1011	2.37	2.28E-34
PA0641	PA0641	5.33	1.34E-28
PA2463	PA2463	14.42	8.6E-25
PA5441	PA5441	2.00	7.17E-10
PA4277	tufB	3.52	2.01E-97
PA3999	dacC	2.19	1.23E-22
PA0594	surA	4.03	1.2E-123
PA0972	tolB	1.98	1.76E-15
PA0636	PA0636	6.34	4.07E-82
PA2624	idh	3.38	3.71E-65
PA0955	PA0955	2.53	5.51E-20
PA5113	PA5113	3.56	1.47E-16
PA2642	nuoG	1.98	1.59E-13
PA3483	PA3483	1.78	6.49E-05
PA1555	ccoP2	18.86	3.1E-163
PA3801	PA3801	3.62	3E-124
PA0045	PA0045	59.74	1.1E-118
PA0622	PA0622	5.02	1.4E-115
PA3716	PA3716	4.30	1.2E-108
PA3115	fimV	3.33	1.38E-90
PA0633	PA0633	4.95	3.4E-89
PA1791	PA1791	13.53	1.84E-64
PA0277	PA0277	8.96	1.29E-58
PA4429	PA4429	2.83	3.53E-58
PA2952	etfB	4.52	1.57E-54
PA1552	ccoP1	3.02	1.32E-48
PA2453	PA2453	11.91	1.62E-40
PA0070	tagQ1	2.88	7.84E-39
PA0623	PA0623	4.49	4.95E-30
PA2997	nqrC	3.37	1.8E-23
PA4372	PA4372	2.57	1.91E-23
PA4480	mreC	3.57	3.37E-23
PA0126	PA0126	4.36	8.3E-23
PA5130	PA5130	2.16	3.72E-18
PA4390	PA4390	2.54	6.6E-16
PA5146	PA5146	2.11	2.24E-14
PA2264	PA2264	2.25	2.06E-10
PA5414	PA5414	2.12	4.08E-10
PA5136	PA5136	2.53	7.8E-07
PA1483	cycH	3.05	9.44E-24
PA3804	PA3804	1.85	2.4E-08
PA4614	mscL	-2.13	0

PA0114	senC	-2.71	7.75E-27
PA1180	phoQ	-35.95	1.3E-175
PA1775	cmpX	-1.63	4.45E-09
PA2815	PA2815	-8.59	5.3E-129
PA2830	htpX	-3.46	8.36E-90
PA3553	arnC	-47.41	8.1E-182
PA4455	PA4455	-1.60	9.21E-10
PA2623	icd	-2.61	0
PA5119	glnA	-2.07	0
PA0588	PA0588	-19.03	1.53E-68
PA2023	galU	-1.41	8.22E-08
PA3531	bfrB	-1.63	0
PA3554	arnA	-10.21	8.1E-70
PA3602	PA3602	-2.40	3.53E-27
PA4022	PA4022	-10.82	2.31E-68
PA4775	PA4775	-2.70	0.000337
PA4525	pilA	-1.39	1.77E-08
PA1053	PA1053	-23.70	0
PA3623	PA3623	-18.21	4.8E-295
PA4735	PA4735	-2.22	4.72E-25
PA2065	pcoA	-10.87	5.84E-12
PA3031	PA3031	-4.28	0
PA3040	PA3040	-7.09	6.1E-165
PA3691	PA3691	-9.08	4.71E-48
PA4495	PA4495	-3.13	2.11E-65
PA4773	PA4773	-2.80	5.2E-12
PA4876	osmE	-16.94	2.4E-213
PA5253	algP	-2.67	0

<u>Table S3:</u> Changes in murine gene expression in response to infection measured by RNA-seq. Fold changes greater than 1 indicate that the gene was up-regulated in *P. aeruginosa* infected mice compared to non-infected mice. Fold changes inferior to -1 indicate that the gene was down-regulated in *P. aeruginosa* infected mice compared to non-infected mice.

**Gene**	**Fold change**	***p*-value**
Csf3	706.23	8.93E-104
Cxcl2	487.41	5.38E-159
Mt2	311.95	1.45E-203
Cxcl1	251.63	2.80E-118
Irg1	223.92	1.71E-44
Ngp	215.56	2.88E-126
Il6	195.34	7.34E-35
Cxcl3	180.12	6.70E-59
Cxcl10	128.13	1.19E-20
Saa3	108.39	7.40E-39
Adamts4	101.25	2.78E-14
Ccl3	100.25	1.79E-119
Ccl4	93.16	1.22E-33
Rsad2	90.82	2.13E-44
Ccl2	89.77	2.76E-28
Cxcl5	87.22	1.08E-21
Cxcl9	86.44	2.26E-11
Tnfaip3	85.2	1.05E-132
Mt1	73.22	2.38E-136
Ccl20	66.12	1.18E-15
Ptx3	65.64	9.86E-09
Ifitm6	65.59	4.32E-23
Gpr84	61.14	1.93E-08
Ccl7	56.16	1.33E-09
Cd14	55.26	2.16E-100
Il1f9	53.72	5.40E-14
Il1r2	53.37	2.51E-09
Slfn4	50.39	1.42E-14
Serpina3f	50.37	2.37E-20
Camp	48.42	1.76E-10
Gm29408	47.15	6.15E-30
Apol8	45.17	8.55E-06
Trem1	43.32	2.06E-13
Oasl1	42.25	1.45E-07
Lilrb4	42.13	2.28E-97
Gm17268	41.49	1.71E-05
S100a9	40.92	1.20E-158

Mx2	39.28	5.88E-12
Socs3	37.97	7.34E-97
Tnf	37.2	3.26E-23
Mmp8	34.28	2.86E-07
Il1rn	34.05	3.73E-15
Gm15287	34	6.61E-05
Retnlg	31.76	4.49E-76
Selp	31.47	6.61E-05
Ccrn4l	30.94	3.10E-20
Lcn2	30.85	7.39E-104
Tmem252	30.62	2.45E-30
Asprv1	30.58	6.53E-10
Gm26809	30.29	1.91E-174
Ifit1	30.28	1.12E-26
AA467197	29.87	5.14E-04
Nlrp3	29.35	8.64E-11
Clec4d	29.34	9.69E-09
Rnd1	28.7	1.82E-13
Gm12840	28.62	5.75E-111
S100a8	28.38	4.61E-134
Gm4951	28.28	2.51E-09
Mrgpra2a	28.07	2.59E-04
Gdf15	26.57	3.49E-13
Prss22	26.52	2.59E-04
Clec4e	26.43	3.33E-10
Gm13086	25.62	1.20E-10
Osm	25.04	1.01E-15
Csf2	24.98	4.44E-11
Gm16242	24.89	1.69E-05
Rab36	24.54	3.32E-10
Nfkbia	24.54	5.95E-101
Adam8	24.01	7.01E-15
Sele	23.9	1.11E-06
Stfa2l1	23.63	1.69E-05
Gm10872	23.21	3.37E-05
Zc3h12a	23.01	1.18E-23
Apold1	23	4.07E-17
Mx1	22.98	3.75E-08
Isg15	22.9	2.13E-21
Plaur	22.37	6.99E-26
Ch25h	21.9	1.37E-34
Irak3	21.72	2.86E-07
Ifit2	21.5	3.80E-18

Irgm1	21.32	3.86E-30
Inhbb	21.24	4.12E-24
E230013L22Rik	21.16	5.82E-09
Gm5483	21.08	5.14E-04
Cdkn1a	21.08	2.25E-30
Art3	20.89	1.31E-04
Gm26545	20.74	5.14E-04
Steap4	20.64	8.80E-12
Cd33	20.58	8.44E-20
Gpr31b	20.55	8.68E-06
Akap12	20.48	3.43E-32
Ptgs2	20.41	2.83E-23
Sphk1	20.05	1.50E-22
Smox	19.9	7.49E-08
Cd80	19.86	2.59E-04
Gm16685	19.81	1.31E-04
Socs1	19.4	4.47E-21
Iigp1	19.29	4.05E-31
Lif	19.26	2.19E-06
Gm13889	19.19	3.40E-138
Slfn1	19.01	8.84E-12
Fut2	18.88	6.61E-05
RP23-354D10.2	18.59	8.81E-40
Gbp5	18.58	1.42E-07
Sfn	18.45	1.08E-28
Il18rap	18.35	2.06E-08
Cldn4	18.31	3.02E-20
Ccl11	18.06	2.19E-06
Gm14023	17.97	1.31E-04
Slc39a14	17.85	9.78E-07
Ccrl2	17.7	1.07E-104
1200007C13Rik	17.58	2.99E-09
Il1b	17.54	2.32E-65
Serpina3i	17.33	6.46E-13
Nfil3	17.32	6.12E-11
Gm20645	17.32	4.63E-32
Rnf125	17.24	1.86E-06
Dll1	17.23	2.72E-14
Sema4c	17.18	9.29E-23
Fam26f	17.17	6.63E-05
Dio2	17.15	3.93E-08
Orm1	17.03	1.31E-04
Gm26667	16.83	5.14E-04

Slfn2	16.79	2.47E-47
Rnf149	16.62	9.76E-14
Cmpk2	16.33	1.21E-12
Tnip1	16.32	1.20E-12
Gm28863	16.23	4.28E-10
Hk2	16.07	2.38E-05
Ptafr	15.93	1.26E-05
Basp1	15.86	1.31E-04
Map3k6	15.65	6.61E-05
Mamstr	15.43	7.49E-08
Thbs1	15.16	5.55E-125
Fosl2	15.09	5.76E-28
Malt1	15	7.99E-12
Gbp2	15	1.20E-14
Birc3	15	8.87E-26
Myc	14.89	4.32E-12
Ccr1	14.88	2.78E-14
Serpina3n	14.72	6.71E-24
Pim1	14.67	2.76E-68
Rasd1	14.6	2.07E-08
Slc7a15	14.48	2.06E-08
Bcl3	14.48	1.56E-09
Hcar2	14.45	1.20E-26
Ltf	14.34	2.38E-05
Itpkc	14.34	2.71E-07
Gm5530	14.31	1.89E-13
Wfdc17	14.27	3.34E-05
Pfkfb3	14.21	1.49E-11
Mmp9	13.82	6.67E-06
Tlr2	13.82	3.84E-35
Sod2	13.81	5.53E-35
Gem	13.62	1.20E-14
Abi1	13.57	3.12E-23
Mefv	13.47	1.31E-04
Rab20	13.42	4.49E-05
Fpr1	13.41	1.20E-09
Ifit3	13.34	1.47E-35
Mxd1	13.33	1.00E-15
Vcan	13.32	2.59E-04
Gm15832	13.32	7.54E-08
Cebpd	13.24	1.86E-120
Ifih1	13.22	2.06E-08
Kcne4	13.05	5.07E-08

Lrrc25	12.88	9.80E-07
Dusp2	12.87	9.96E-11
Il1a	12.83	5.20E-07
Ccl17	12.77	2.60E-04
Txnrd1	12.74	1.94E-11
Casp4	12.69	4.49E-05
Gm20400	12.66	2.71E-07
Gm12248	12.63	5.56E-04
Tnfaip2	12.52	3.18E-20
Serpina3h	12.49	1.95E-11
Rbbp8	12.44	3.44E-10
Cd274	12.44	2.19E-16
Marcksl1	12.42	7.63E-40
Cish	12.37	3.60E-19
Smim3	12.26	1.26E-05
Slc15a3	12.2	9.29E-08
Dbn1	12.03	6.66E-06
Nfkbiz	11.94	4.20E-18
Arid5a	11.72	2.23E-10
Csrnp1	11.63	5.59E-04
Srgn	11.61	8.63E-15
Rnf19b	11.57	9.30E-08
Chd9	11.46	4.18E-09
F3	11.43	1.56E-14
Icam1	11.41	1.43E-119
Gm9115	11.3	9.43E-08
Trib1	11.19	2.42E-22
Spry4	11.17	2.49E-17
Sp140	11.16	2.38E-05
Ddit4	11.08	1.25E-14
Parp14	11.05	1.49E-16
Cxcr2	10.9	8.43E-09
P2ry2	10.86	1.85E-06
Dennd4a	10.86	7.79E-09
Daxx	10.75	1.06E-11
Plscr1	10.72	2.52E-09
Prr7	10.66	1.26E-05
Irgm2	10.59	2.52E-09
Themis2	10.56	8.41E-09
Odc1	10.55	4.13E-10
Sgms2	10.38	4.48E-05
Abtb2	10.37	2.97E-04
Sbno2	10.34	3.57E-11

Lmnb1	10.32	2.97E-04
Hif1a	10.28	5.03E-08
Pik3ap1	10.17	6.75E-06
Ackr3	9.97	6.45E-12
Csf1	9.9	4.17E-10
Hdc	9.84	5.85E-14
Rel	9.82	1.26E-05
Egr1	9.75	1.53E-78
Klhl6	9.74	2.38E-05
Sdc4	9.72	1.42E-32
Ampd3	9.62	1.59E-04
Osmr	9.6	4.60E-09
Map3k8	9.55	9.25E-08
Tiparp	9.49	4.62E-09
Maff	9.26	2.52E-09
Kdm6b	9.14	9.36E-19
Zfp46	9.06	5.32E-17
Litaf	9.02	1.59E-04
Glrx	9.02	1.36E-09
Tubb6	8.99	2.00E-06
C5ar1	8.86	1.36E-04
Ntng2	8.84	2.97E-04
Plk3	8.81	2.00E-06
Il10ra	8.77	1.68E-07
Ppp1r15a	8.76	7.75E-32
Fmnl2	8.68	4.48E-05
Tbce	8.59	6.24E-12
9030624G23Rik	8.58	8.44E-05
Arg2	8.54	1.35E-04
I830012O16Rik	8.44	1.37E-09
Gm20689	8.43	1.56E-10
Irf1	8.4	8.89E-25
Plat	8.36	8.44E-05
Serpine1	8.36	8.50E-20
Adamts9	8.35	6.74E-06
Dhx40	8.34	4.11E-05
Nfkb2	8.34	1.36E-09
Tap1	8.33	6.76E-11
Pgs1	8.3	1.36E-09
RP23-27C10.1	8.23	1.59E-04
Plek	8.23	5.47E-07
Ifitm5	8.2	7.56E-05
Neat1	8.2	3.65E-109

Nampt	8.14	1.78E-06
Gm14928	8.09	2.92E-09
Cd53	8.07	6.76E-06
Fas	8	7.48E-05
Oasl2	8	5.72E-06
Mapk6	7.97	9.88E-07
N4bp1	7.96	4.53E-08
Irf7	7.91	1.02E-05
Pdlim7	7.9	3.19E-06
Fcer1g	7.85	3.04E-07
Hck	7.84	2.45E-04
Ccl5	7.8	6.77E-06
Rtp4	7.77	1.43E-08
Tuba1c	7.74	5.50E-07
Parp9	7.67	4.11E-05
Nfkbie	7.66	6.74E-06
Il4ra	7.6	8.18E-15
Dgat1	7.56	8.05E-07
Rnf213	7.5	1.21E-09
Ehd1	7.42	4.56E-15
Ier3	7.38	2.53E-151
Serpina3g	7.33	2.51E-06
Gm12250	7.31	2.19E-09
Gvin1	7.27	4.41E-04
Gadd45b	7.25	2.08E-31
Pmaip1	7.23	3.19E-06
Tnfrsf12a	7.16	1.26E-08
Phlda1	7.01	4.65E-25
Rps3a3	6.98	3.97E-10
Syk	6.96	1.02E-05
Stx11	6.93	3.19E-06
Gadd45g	6.91	1.38E-11
Helz2	6.91	4.71E-20
Bhlhe40	6.89	3.25E-18
Tnfrsf1b	6.87	5.71E-06
Dusp16	6.73	1.01E-04
Junb	6.71	7.42E-82
Bcl2l11	6.66	1.01E-04
Myd88	6.65	5.82E-05
Tpd52	6.64	4.40E-04
Nelfe	6.63	3.10E-04
Retsat	6.6	7.69E-06
Gm17709	6.54	2.45E-04

Nfkb1	6.54	7.68E-06
Slc6a14	6.49	2.45E-04
Rnd3	6.43	2.34E-05
Errfi1	6.42	6.85E-24
Cebpb	6.41	3.34E-38
Furin	6.38	9.19E-13
Rab8b	6.32	1.02E-05
Ogfr	6.32	4.16E-09
Gbp8	6.29	2.45E-04
Btg1	6.29	3.92E-40
Anxa1	6.28	9.54E-09
Tbk1	6.27	1.01E-04
Rabgef1	6.27	4.04E-05
Fth1	6.27	9.35E-56
Ptpn1	6.24	1.83E-06
Slc25a25	6.22	7.88E-04
Slc16a13	6.2	1.34E-05
Rhoc	6.18	5.71E-15
Mapkapk2	6.15	3.66E-08
Vcam1	6.12	5.47E-06
Gadd45a	6.12	1.66E-08
Chmp4b	6.08	1.01E-04
Sntb2	6.06	7.88E-04
Xdh	6.06	4.05E-05
Slc27a4	5.96	7.88E-04
Trafd1	5.94	3.17E-06
Snord17	5.87	1.24E-26
Hbegf	5.86	1.20E-04
Map2k3	5.85	5.71E-05
Robo4	5.84	1.49E-07
Xaf1	5.82	1.77E-04
Gramd1a	5.77	1.77E-04
Birc2	5.77	4.40E-07
Clic4	5.75	4.61E-21
Zc3hav1	5.73	9.38E-06
Fbxl5	5.72	4.40E-04
Gm12854	5.71	3.32E-06
Pvr	5.7	7.88E-04
Gm26982	5.68	1.78E-04
Lipg	5.67	3.10E-04
Mob3c	5.65	1.77E-04
Gm27019	5.65	2.75E-05
Dtx3l	5.61	1.83E-06

Ets2	5.57	4.69E-13
Tgif1	5.56	5.39E-04
Gm6485	5.56	1.75E-13
Zfp36	5.53	1.63E-125
Gm20603	5.49	5.59E-11
Cd63-ps	5.47	1.97E-08
Sema7a	5.45	9.35E-04
Gm6977	5.45	1.64E-121
Etv3	5.41	4.41E-07
Tgm2	5.4	2.31E-10
Gm23444	5.36	3.95E-08
Stk19	5.35	9.35E-04
Cd44	5.34	1.25E-07
Hsp25-ps1	5.28	1.73E-56
Tet2	5.2	3.10E-04
Il17ra	5.17	7.88E-05
Aff1	5.16	6.98E-05
Gm22486	5.1	5.50E-04
Plekho2	5.06	5.95E-04
Ncf1	4.97	5.39E-04
Rnasel	4.97	3.51E-04
Cfb	4.97	1.80E-05
Pim3	4.96	1.19E-11
Stat3	4.94	1.16E-05
Ier2	4.93	1.43E-61
Ralgds	4.9	9.35E-04
Rac2	4.88	2.06E-04
Rnf19a	4.87	2.23E-04
Bach1	4.85	4.65E-05
Cp	4.82	3.02E-05
Snx18	4.81	6.99E-06
Trim25	4.81	1.65E-06
Egln2	4.81	9.37E-08
Skil	4.8	1.65E-06
H2-Q4	4.8	2.41E-18
H3f3b	4.79	2.39E-41
Gm4070	4.76	9.35E-04
RP24-252K15.3	4.75	4.61E-10
F630028O10Rik	4.7	4.66E-05
Rgcc	4.69	5.04E-05
Taf7	4.69	3.19E-05
Gm8995	4.69	3.47E-14
Ankrd12	4.67	5.96E-04

Ccl9	4.64	3.51E-04
Enc1	4.61	2.06E-04
Dennd3	4.61	6.16E-04
Ccnl1	4.6	1.01E-07
Ppp1r18	4.57	4.19E-06
Klf6	4.55	1.65E-29
Igfbp3	4.44	1.59E-11
4933426M11Rik	4.43	1.99E-05
Gm23971	4.43	2.56E-07
Per1	4.37	3.25E-05
Sik1	4.37	3.83E-10
42068	4.35	6.17E-04
Il31ra	4.34	2.47E-04
Rhob	4.23	1.67E-82
Dusp1	4.23	1.74E-78
Prss23	4.18	2.27E-04
Rrs1	4.15	3.28E-05
H2-Q6	4.14	7.71E-06
Riok3	4.13	1.40E-04
Ier5	4.09	1.27E-14
Etf1	4.02	3.75E-04
Irf9	4.02	3.75E-04
Pxdc1	4.02	2.00E-05
Bcl2l1	4	2.28E-04
Gna13	4	1.24E-05
Gja1	3.99	1.83E-06
Glul	3.98	1.90E-13
Edn1	3.97	7.65E-06
Grina	3.95	7.33E-13
Gm23472	3.89	8.59E-05
Lyve1	3.88	2.71E-11
Nabp1	3.86	9.90E-04
Gclc	3.85	1.40E-04
S1pr1	3.84	5.39E-29
Zfp281	3.83	4.38E-07
Znfx1	3.82	2.26E-04
Srxn1	3.81	6.11E-04
Lrg1	3.81	4.28E-28
Mcl1	3.77	8.89E-38
Efhd2	3.76	6.11E-04
Rela	3.76	2.99E-05
Picalm	3.74	1.23E-05
Ppp1r15b	3.72	1.84E-10

Rab7	3.71	2.26E-04
Sav1	3.68	8.50E-05
Prdx5	3.64	1.80E-05
2810474O19Rik	3.62	2.69E-06
Carhsp1	3.58	7.83E-05
Rasip1	3.57	2.52E-07
Rab11fip1	3.55	4.42E-06
Gm26316	3.54	3.06E-05
Adamts1	3.51	3.93E-13
Gm12966	3.51	8.89E-26
Csf2ra	3.5	5.52E-04
Stk17b	3.47	9.37E-04
St3gal1	3.47	1.00E-06
Eif1	3.47	2.89E-28
Tnks1bp1	3.45	4.38E-05
Mir6236	3.45	1.97E-04
Eif5	3.43	1.39E-07
Sspn	3.43	3.75E-13
Id2	3.39	5.26E-12
Rnu3b2	3.37	1.81E-05
Rnu3a	3.32	7.67E-06
Gm10020	3.29	2.09E-11
Gm15801	3.27	8.00E-04
Cflar	3.27	5.09E-04
Bcl6b	3.25	4.64E-04
Marcks	3.25	7.63E-16
Mafk	3.2	4.64E-04
Tacstd2	3.19	1.75E-10
Rnu3b3	3.16	5.48E-05
Cxcl16	3.14	6.24E-05
Rnu3b4	3.12	4.07E-04
Tpm3	3.09	1.61E-04
Arhgap21	3.09	4.17E-18
Ppp1r2-ps2	3.03	4.30E-05
Sqstm1	3.03	1.56E-08
Lox	3.02	7.21E-06
Slfn5	3.01	2.01E-05
Ptp4a1	2.99	1.53E-05
Gm9835	2.99	2.63E-07
Ifitm3	2.98	5.87E-24
Hba-a2	2.96	3.63E-13
Rap1b	2.91	5.03E-04
Rnu3b1	2.91	1.10E-05

Tpm3-rs7	2.9	1.18E-29
Jarid2	2.89	9.53E-05
Cd47	2.88	9.71E-04
H2-K1	2.88	3.96E-18
Spry2	2.87	5.05E-04
Ddx3x	2.83	3.02E-10
Slc38a2	2.8	1.03E-04
Ctla2a	2.78	3.30E-06
Gm24950	2.77	7.86E-06
Myh9	2.72	3.85E-04
Rpl14-ps1	2.72	5.19E-12
Midn	2.71	1.99E-04
Zfp703	2.71	2.21E-05
Gm10232	2.68	5.63E-05
Ppp1r2-ps4	2.67	7.83E-14
Gm23849	2.65	3.01E-07
Hspb1	2.64	4.69E-15
Cdk8	2.64	2.08E-14
Gm10156	2.58	3.75E-05
Gm8822	2.56	8.18E-08
Gm23119	2.55	5.68E-04
Eif3s6-ps2	2.54	5.05E-04
Hbb-bt	2.54	1.08E-16
Eif1-ps1	2.52	1.10E-05
Atf4	2.49	1.61E-12
Yam1	2.48	2.51E-04
Gm15453	2.46	5.36E-06
Gm9791	2.45	4.13E-04
Tapbp	2.41	1.11E-05
1810049J17Rik	2.41	2.23E-09
Esam	2.39	6.83E-04
Gm8203	2.39	1.49E-04
Kctd12	2.36	1.87E-04
Gm7809	2.36	3.40E-07
Xbp1	2.35	3.20E-05
Ifitm2	2.33	9.74E-15
Eif4a1	2.28	8.21E-04
Gm9855	2.28	5.06E-04
Erdr1	2.28	3.58E-04
AY036118	2.27	7.06E-07
Gm9845	2.26	4.64E-05
Cmss1	2.26	4.87E-15
Gm10254	2.25	2.38E-05

Srsf5	2.24	5.50E-07
Gm15487	2.24	2.05E-05
Gm7334	2.23	5.87E-09
Sat1	2.2	2.23E-06
Gm1821	2.18	3.02E-13
Rnu2-10	2.17	1.52E-04
Jund	2.17	2.76E-13
Slc39a1	2.16	8.04E-04
Hba-a1	2.11	5.87E-09
Rps27a-ps2	2.09	4.50E-07
Hbb-bs	2.07	4.82E-19
Gm12895	2.02	5.26E-04
n-R5-8s1	2.02	1.17E-20
Prdx6	1.96	3.38E-05
Gm13394	1.95	9.10E-04
Ubc	1.89	4.60E-19
Gm9762	1.88	3.99E-05
Ctgf	1.85	4.41E-06
H2-D1	1.84	1.49E-15
Gm13370	1.81	5.90E-04
Gm12715	1.81	3.80E-26
Tgoln1	1.79	1.22E-04
Gm12183	1.78	9.36E-10
Gm15441	1.77	1.88E-04
Rps13-ps2	1.76	7.88E-05
Gm8430	1.74	2.54E-08
Hspa5	1.7	3.79E-05
Clec2d	1.7	9.72E-04
Gm26825	1.68	1.29E-20
Eif4a-ps4	1.63	9.93E-09
Actg1	1.63	6.80E-12
Scgb1a1	1.63	1.90E-19
Gm26917	1.61	7.71E-46
Jun	1.6	5.42E-07
Rps23-ps	1.6	3.89E-04
Zfp36l1	1.6	9.94E-04
Gm4202	1.51	1.41E-08
Gm10116	1.5	4.20E-10
Scarna10	1.5	2.22E-04
Rps27rt	1.48	7.24E-09
Tpt1-ps3	1.46	1.14E-08
1700020I14Rik	-4.4	4.20E-04
Actb	-1.02	4.86E-11

Acvrl1	-4.52	1.33E-05
Adrb1	-7.43	1.10E-06
Ager	-2.25	1.51E-10
Akap5	-3.9	1.73E-10
Aplnr	-11.92	1.44E-09
Calcrl	-14.56	2.15E-08
Calr	-3.1	3.59E-06
Cbr2	-3.35	1.49E-34
Ccl21a	-4.25	4.86E-05
Cldn3	-2.47	7.35E-05
Clec14a	-6.05	3.69E-20
Clec1a	-23.52	2.98E-04
Cnn2	-3.2	8.24E-04
Crip2	-3.99	1.36E-04
Cxcl12	-9.67	5.29E-07
Cxcl15	-7.77	1.20E-28
Cxx1a	-6.22	1.79E-07
Cxx1b	-8.5	9.34E-10
Cyp2f2	-4.34	5.27E-08
Dynll1	-8.19	6.41E-06
Dynlt1-ps1	-5.22	8.25E-19
Ear-ps2	-10.23	1.14E-09
Ear2	-4.53	4.29E-11
Egfl7	-10.14	6.36E-08
Eid1	-4.02	4.61E-04
Elovl1	-4.94	3.99E-06
Fzd2	-20.73	5.57E-04
Gm10080	-2.06	2.44E-04
Gm10123	-2.3	1.80E-15
Gm10175	-3.68	5.37E-10
Gm10179	-5.09	3.05E-04
Gm10193	-3.84	1.26E-04
Gm10243	-3.48	3.52E-04
Gm10263	-2.57	5.67E-13
Gm10275	-1.86	9.40E-07
Gm10288	-1.82	4.53E-06
Gm10800	-16.28	1.67E-25
Gm11223	-6.48	1.10E-07
Gm11808	-1.9	1.05E-09
Gm12184	-3.29	8.33E-04
Gm13443	-2.93	1.79E-05
Gm13502	-6.55	1.19E-06
Gm13597	-3.22	2.44E-04

Gm15501	-1.88	1.90E-05
Gm15772	-1.93	4.52E-09
Gm15920	-14.01	1.05E-05
Gm22179	-2.33	5.81E-08
Gm2423	-2.37	1.24E-04
Gm24265	-5.72	6.39E-10
Gm26191	-1.94	6.45E-07
Gm4294	-3.6	6.70E-07
Gm5525	-3.34	3.41E-04
Gm5644	-2.81	7.97E-06
Gm6166	-3.53	2.88E-04
Gm6969	-2.18	6.55E-04
Gm6984	-2.57	1.89E-04
Gm7536	-1.73	1.35E-05
Gm8186	-3.44	4.63E-04
Gm8894	-2.76	3.86E-06
Gm9770	-2.86	9.90E-07
Gm9824	-8.25	7.92E-05
Gm9840	-3.49	7.92E-04
Gm9844_1	-4.73	2.01E-04
H1f0	-4.66	1.56E-08
Hist1h1a	-5.29	3.56E-04
Hist1h1d	-3.05	1.46E-04
Hist1h1e	-3.02	9.34E-15
Hist1h2ac	-7.89	2.26E-04
Hist1h2ai	-5.32	3.34E-05
Hist1h2ak	-4.71	2.83E-04
Hist1h2al	-2.72	1.91E-06
Hist1h2ap	-3.18	2.78E-05
Hist1h2bg	-2.56	3.66E-04
Hist1h2bk	-4.14	1.20E-06
Hist1h2bl	-6.01	8.41E-10
Hist1h2bm	-10.92	3.26E-06
Hist1h3b	-3.87	4.63E-04
Hist1h3d	-6.85	1.11E-09
Hist1h4c	-3.1	3.53E-07
Hist1h4d	-2.59	1.95E-11
Hist1h4h	-4.91	1.63E-21
Hist1h4i	-4.2	5.75E-04
Hist1h4n	-5.62	8.51E-16
Hist2h2ac	-5.41	3.03E-06
Hist2h3c2	-5.4	4.97E-04
Hist2h4	-4.78	1.62E-04

Hpgd	-15.86	2.27E-05
Hspa1a	-42.43	4.49E-12
Hspa1b	-12.06	8.83E-17
Igkc	-2.42	8.31E-07
Inmt	-4.49	3.36E-27
Lbh	-22.94	9.36E-08
Lgi3	-5.9	5.31E-05
Lyz2	-2.62	1.16E-27
Malat1	-1.19	4.60E-04
Mfap4	-2.15	4.15E-04
Mrfap1	-2.31	7.88E-05
Mrpl34	-9.54	3.53E-04
mt-Atp6	-2	2.22E-05
mt-Co1	-1.83	1.62E-10
mt-Co2	-2.04	0
mt-Nd4	-1.67	5.81E-06
Nkx2-1	-6.68	1.66E-07
Nr1d1	-8.88	1.33E-04
Nrarp	-13.36	1.78E-04
Nutf2-ps1	-3.6	4.66E-04
Ppdpf	-5.86	1.38E-04
Retnla	-6.91	1.94E-05
Rgs2	-5.25	1.16E-05
Rmrp	-1.72	3.91E-07
Rn7sk	-1.2	6.78E-04
Rnf181	-4.94	4.41E-05
Rnu11_1	-19.81	4.42E-04
RP23-187F18.1	-4	3.91E-05
Rpl10-ps3	-2.61	3.04E-06
Rpl10a-ps1	-1.83	3.94E-06
Rpl15	-4.43	2.26E-04
Rpl17-ps9	-4.19	5.78E-17
Rpl19-ps11	-1.64	8.91E-04
Rpl23a-ps3	-1.71	2.83E-04
Rpl31-ps8	-2.15	9.18E-10
Rpl34-ps1	-1.63	3.54E-04
Rpl39-ps	-2.33	4.83E-05
Rps13-ps1	-1.85	3.87E-04
Rps15a-ps7	-2.25	3.77E-05
Rps24-ps3	-2.51	1.53E-10
Rps26-ps1	-2.66	2.35E-04
Rps6-ps4	-1.83	2.70E-06
Rsrp1	-3.87	1.66E-07

Scd1	-2.62	2.34E-11
Scd2	-2.5	1.61E-04
Scgb3a2	-4.95	3.59E-08
Scp2-ps2	-3.42	1.92E-09
Sema3g	-8.31	9.36E-04
Serpinh1	-4.38	3.95E-06
Sftpa1	-1.8	1.67E-06
Sftpb	-2.97	1.22E-09
Sftpc	-2.9	1.94E-22
Slc34a2	-4.32	1.06E-10
Slc9a3r2	-7.63	1.82E-05
Sox17	-17.44	4.41E-04
Sox18	-37.73	1.13E-17
Tbx2	-4.98	1.70E-05
Thbd	-2.15	4.46E-07
Tmsb4x	-1.79	1.11E-05
Tppp3	-4.4	7.77E-05
Tuba1a	-2.22	6.76E-04
Tuba1b	-2.97	7.95E-06
Tubb5	-3.06	3.65E-07
